# Comprehensive Linkage and Association Analyses Identify Haplotype, Near to the *TNFSF15* Gene, Significantly Associated with Spondyloarthritis

**DOI:** 10.1371/journal.pgen.1000528

**Published:** 2009-06-19

**Authors:** Elena Zinovieva, Catherine Bourgain, Amir Kadi, Franck Letourneur, Brigitte Izac, Roula Said-Nahal, Nicolas Lebrun, Nicolas Cagnard, Agathe Vigier, Sébastien Jacques, Corinne Miceli-Richard, Henri-Jean Garchon, Simon Heath, Céline Charon, Delphine Bacq, Anne Boland, Diana Zelenika, Gilles Chiocchia, Maxime Breban

**Affiliations:** 1 Institut Cochin, Université Paris Descartes, CNRS (UMR 8104), Paris, France; 2 INSERM U567, Paris, France; 3 INSERM U535, Université Paris Sud – Paul Brousse Hospital, Villejuif, France; 4 Rheumatology Division, Ambroise Paré Hospital (AP-HP), and Versailles Saint Quentin en Yvelines University, Boulogne-Billancourt, France; 5 Bioinformatics Platform, Faculty of Medicine Paris Descartes, Necker Hospital, Paris, France; 6 Rheumatology Division, Kremlin-Bicêtre Hospital (AP-HP), Kremlin-Bicêtre, France; 7 National Genotyping Center (CNG), Evry, France; University of Washington, United States of America

## Abstract

Spondyloarthritis (SpA) is a chronic inflammatory disorder with a strong genetic predisposition dominated by the role of *HLA-B27*. However, the contribution of other genes to the disease susceptibility has been clearly demonstrated. We previously reported significant evidence of linkage of SpA to chromosome 9q31–34. The current study aimed to characterize this locus, named SPA2. First, we performed a fine linkage mapping of SPA2 (24 cM) with 28 microsatellite markers in 149 multiplex families, which allowed us to reduce the area of investigation to an 18 cM (13 Mb) locus delimited by the markers D9S279 and D9S112. Second, we constructed a linkage disequilibrium (LD) map of this region with 1,536 tag single-nucleotide polymorphisms (SNPs) in 136 families (263 patients). The association was assessed using a transmission disequilibrium test. One tag SNP, rs4979459, yielded a significant *P*-value (4.9×10^−5^). Third, we performed an extension association study with rs4979459 and 30 surrounding SNPs in LD with it, in 287 families (668 patients), and in a sample of 139 cases and 163 controls. Strong association was observed in both familial and case/control datasets for several SNPs. In the replication study, carried with 8 SNPs in an independent sample of 232 cases and 149 controls, one SNP, rs6478105, yielded a nominal *P*-value<3×10^−2^. Pooled case/control study (371 cases and 312 controls) as well as combined analysis of extension and replication data showed very significant association (*P*<5×10^−4^) for 6 of the 8 latter markers (rs7849556, rs10817669, rs10759734, rs6478105, rs10982396, and rs10733612). Finally, haplotype association investigations identified a strongly associated haplotype (*P*<8.8×10^−5^) consisting of these 6 SNPs and located in the direct vicinity of the *TNFSF15* gene. In conclusion, we have identified within the SPA2 locus a haplotype strongly associated with predisposition to SpA which is located near to *TNFSF15*, one of the major candidate genes in this region.

## Author Summary

Spondyloarthritis (SpA) is a common variety of articular inflammatory disorder characterized by axial and/or peripheral arthritis, frequently associated with extra-articular manifestations such as psoriasis, uveitis, and inflammatory bowel diseases (ulcerative colitis or Crohn's disease (CD)). SpA is a complex disorder with high heritability. The MHC class I *HLA-B27* allele is a very strong risk factor for its development, but other genetic factors located outside the MHC also play a role in disease susceptibility. By a previous whole-genome linkage investigation, we have demonstrated that a region located on the chromosome 9q31–34 was involved in SpA susceptibility. The present study aimed to further characterize this locus. Using a stepwise linkage and association approach, we identified a haplotype spanning 6 single-nucleotide polymorphisms strongly associated with SpA and located in a genomic region paralogous to the MHC, near to the *TNFSF15* gene. Interestingly, polymorphisms of this gene have previously been shown to be associated with CD. This original finding offers a new research track for the understanding of SpA pathophysiology, which is still poorly understood, as well as new hope for diagnostic and therapeutic innovation.

## Introduction

Spondyloarthritis (SpA) is one of the most frequent varieties of articular inflammatory disorders with an estimated prevalence of 0.3% in the western European adult population [1]. It is characterized by a predominant axial skeleton inflammation, by a frequent occurrence of enthesitis and peripheral arthritis, and also by a high rate of extra-articular features, the most characteristic of which are acute anterior uveitis, psoriasis, and inflammatory bowel diseases (such as ulcerative colitis or Crohn's disease (CD)) [2]. Depending on its clinical features, SpA is classically subdivided into the following subsets: ankylosing spondylitis (AS), which is the prototypical form characterized by predominant axial skeletal involvement and advanced radiographic sacroiliitis, psoriatic arthritis (PsA), arthritis associated with inflammatory bowel disease (AIBD), reactive arthritis (ReA), and undifferentiated SpA (uSpA). Familial aggregation among these conditions has been well established. Notably, we have previously shown, by analyzing a large number of pedigrees with multiple cases of SpA, that all subtypes are likely to be determined by a core set of predisposing factors and may therefore be studied together in genetic studies [3]–[5].

The *HLA-B27* allele is the first genetic factor which was demonstrated to be associated with AS [6],[7] and other SpA [4],[5],[8]. Although about 80% of Caucasian patients are *HLA-B27* positive, as compared to only 6–8% in the general population, the exact mechanism for this association remains poorly understood [9].

Family and twin studies have demonstrated additional non-MHC susceptibility regions elsewhere in the genome [10]. For example, concordance rates for *HLA-B27* positive monozygotic twins are twice as high as the concordance rates for HLA-B27 positive dizygotic twins [10]. Furthermore, the involvement of genetic factors arising from outside the HLA region is suggested by the large non-HLA component of the relative recurrence risk for the SpA estimated in sib-pairs (λ_non-HLA_). Indeed, if the overall relative recurrence risk in sibling (λs) has been estimated to be 40 [11], estimates of the λs component attributable to the HLA region (λ_HLA_), based on previous affected sib-pairs linkage analyses, ranges from 5.2 to 6.25 [12],[13]. Variants in several genes such as the *IL-1* family gene cluster [14],[15], *IL-23R* [16], and *ARTS1/ERAP1* [16], have recently been reported to be associated with AS based on a candidate-gene approach [14],[15] or a non-synonymous single-nucleotide polymorphisms (SNPs) genome-wide association study [16].

Our team has previously reported results of the first genome-wide linkage screen and its extension study performed in SpA [13]. Overall, 893 individuals from 120 multiplex families (families with several patients) comprising 336 affected relative pairs have been genotyped in this study. Non parametric multipoint linkage analysis of the whole dataset yielded evidence for significant linkage to the chromosomal region 9q31–34 (NPLmax = 4.87, *P* = 2×10^−5^). This locus overlapped with one of those identified by the genome-wide linkage screen performed in AS by a group from Oxford [12]. We named this new susceptibility location SPA2, in reference to the MHC locus, which we considered as SPA1 [13]. SPA2 encompasses a 23.95 cM region (17.44 Mb) containing 85 genes and predicted coding sequences as well as 110 pseudogenes. This locus is very appealing with regard to SpA susceptibility. First of all, it is one of three genomic regions paralogous to the MHC, which is the major SpA susceptibility region [17],[18]. Furthermore, it is syntenic to the *Pgis*2 susceptibility locus mapped in a murine model of SpA [19]. Within its borders SPA2 contains both the *TNFSF15* gene found to be associated with CD a condition belonging to the SpA spectrum [20],[21], and the *TRAF1*-*C5* locus associated with rheumatoid arthritis another inflammatory rheumatic disease [22],[23].

The goal of the present study was to identify variants associated with the disease and located in the SPA2 locus. Using a comprehensive four-step linkage and association study in a total of 287 families including 668 affected individuals, followed by an independent case/control analysis (2 samples including a total of 371 cases and 312 controls), we identified a strongly associated six-SNPs haplotype, located at 28.6 kb from the *TNFSF15* gene.

## Results

### Linkage fine mapping

**Figure 1:**
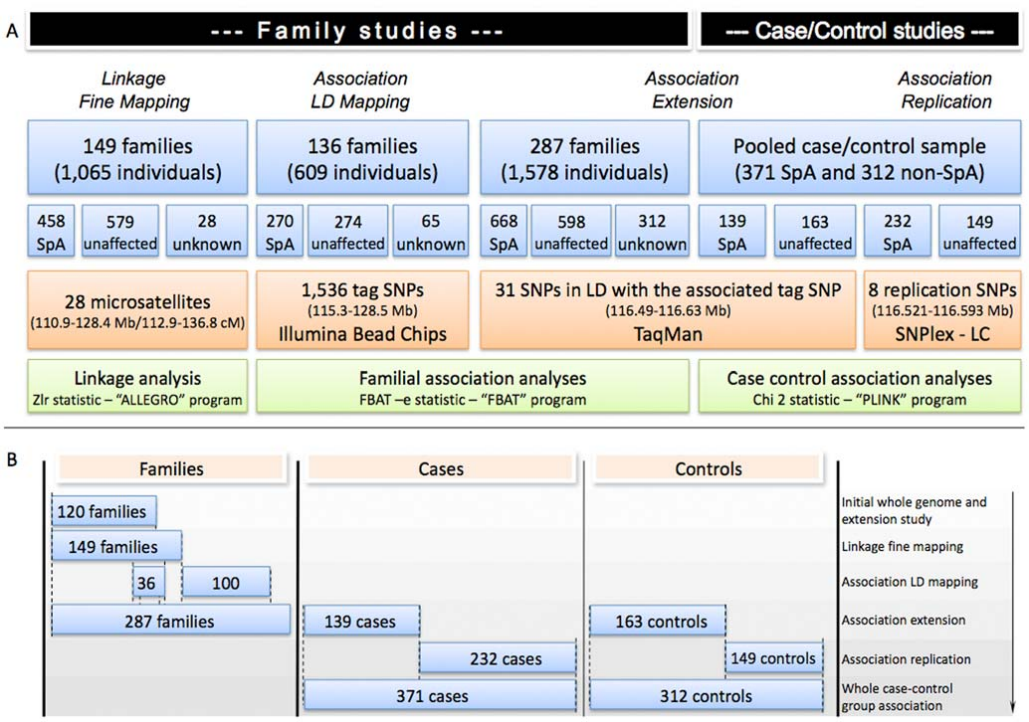
Study design. (A) shows the different stages of the study displayed according to their chronological order from left to right. Frames in blue describe the familial and case/control samples used for each different step. Frames in orange describe the sets of markers, the limits of the intervals covered by these markers on chromosome 9 (in distance from the p-telomere), and the techniques used for single-nucleotide polymorphisms initial genotyping. Frames in green describe the type of genetic analysis performed, the statistic, and the program used in each case. (B) shows compositions of family and case/control samples used in each step of the study. The three first columns show for each step of the study which familial, and case and/or control sample respectively, was used. The far right column displays, for each genotyped sample, the step of the study in which it was involved. The overlay parts of the samples are matched. Ex: Of the 136 families included in the LD mapping association study, 36 were also used in the fine mapping linkage study and in the initial whole genome and extension screen. NB: Both case/control samples were composed of independent individuals not tested elsewhere. Abbreviations: LD: linkage disequilibrium, SpA: spondyloarthritis, SNP: single-nucleotide polymorphism.

The initial step of our study aimed to refine the linkage signal in the 23.95 cM (17.44 Mb) SPA2 locus. To realise this investigation we selected a fine-grained set of 28 microsatellite markers (more than one marker per cM). These markers were genotyped in 149 independent multiplex families (including the 120 families studied in our initial genome-screen) [13] (Figure 1B) consisting of 1,065 individuals including 458 affected with SpA (Figure 1A, Table 1).

**Table 1:** Pairwise distributions among first and second degree relative pairs included in family-based study designs.

Study design	Linkage fine mapping	Linkage disequilibrium mapping	Extension study
Sib-pairs	251	73	266
Half-sibs	6	5	8
Cousins	54	16	35
Parent/child	142	94	211
Grand parent	4	1	8
Avuncular pairs^a^	99	21	78
*Total*	*556*	*210*	*606*

a Niece/aunt, niece/uncle, nephew/aunt, nephew/uncle pairs.

For each family-based study design, the number of each affected relative pairs type included in the study is displayed.

**Figure 2:**
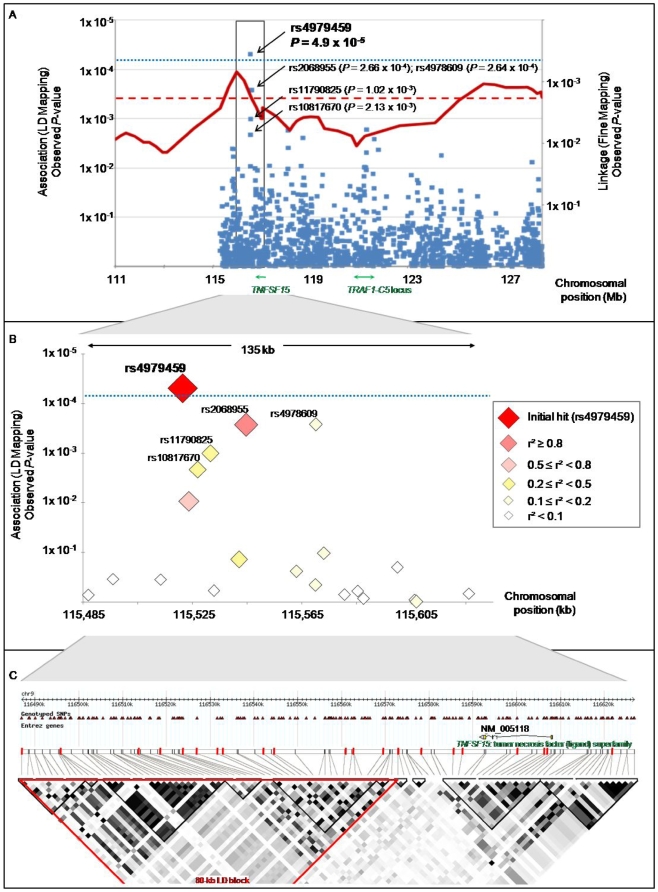
Results of linkage disequilibrium mapping association study. (A) shows results of both fine mapping linkage step (right y axis) and LD mapping step (left y axis). Linkage results are shown in red, with the dashed straight line symbolizing the 0.05 Bonferroni corrected significance threshold. Family-based association results are displayed in blue, with each diamond corresponding to a studied tag SNP. The dotted straight line shows the 0.075 Bonferroni corrected significance threshold. In green are represented the locations of the *TNFSF15* gene, associated with Crohn's disease and of the *TRAF1-C5* locus associated with rheumatoid arthritis. (B) shows a zoomed view of the 135 kb region of interest delineated by frames in (A). Each diamond, corresponding to a studied tag SNP, appears coded by color and size, according to its linkage disequilibrium correlation coefficient (r^2^) with the most significantly associated tag SNP (rs4979459), as displayed in the legend. (C) shows linkage disequilibrium structure across the 135 kb region zoomed in (B), based on r^2^ coefficient calculated with the CEU HapMap database. The intron exon structure of the *TNFSF15* gene lying within this locus is represented at the top of (C). The genotyped tag SNPs shown in (B) are indicated with red bars. Three of these tag SNPs lie within the *TNFSF15* gene.

Non parametric multipoint linkage analysis allowed us to identify two prominent linkage peaks yielding a significant Zlr value>2.91 (nominal *P*<1.79×10^−3^) corresponding to a *P*<0.05 after correction for multiple testing (Table 2, Figure 2A). The highest peak of linkage was found for the marker D9S1824 at 120.1 cM from the p-telomere (Zlr = 3.20; nominal *P* = 6.94×10^−4^). At this stage of the study it was not possible to discriminate between these two peaks, thus we decided to pursue our investigations in the 13.1 Mb region surrounding them between D9S279 and D9S112 (Figure 2A).

**Table 2:** Results of linkage analysis of 28 microsatellite markers spanning the SPA2 locus.

Marker	Genetic location (cM)	Physical location (bp)	Zlr	nominal *P*	Information content
**D9S1677**	112.9	110,977,411–110,977,668	2.38	8.68×10^−3^	0.97
D9S160	113.2	111,418,819–111,418,976	2.47	6.84×10^−3^	0.97
D9S1675	114.4	112,128,955–112,129,173	2.34	9.68×10^−3^	0.99
D9S1854	114.4	112,149,837–112,150,087	2.34	9.69×10^−3^	0.99
D9S1828	114.4	112,372,536–112,372,710	2.34	9.69×10^−3^	0.99
D9S1683	115.1	112,903,157–112,903,331	2.19	1.41×10^−2^	0.99
D9S1880	115.1	113,085,561–113,085,752	2.19	1.41×10^−2^	0.99
D9S279	118.8	115,267,469–115,267,714	2.78	2.68×10^−3^	0.96
D9S1824	120.1	115,931,926–115,932,043	3.20	6.94×10^−4^	0.96
D9S1855	121.3	116,550,769–116,550,989	2.90	1.89×10^−3^	0.99
D9S155	121.4	116,938,283–116,938,406	2.65	3.98×10^−3^	1.00
**D9S1776**	121.4	116,999,255–116,999,373	2.66	3.97×10^−3^	1.00
D9S177	121.5	116,999,255–116,999,373	2.79	2.63×10^−3^	1.00
D9S170	123.0	118,107,200–118,107,315	2.51	6.11×10^−3^	0.99
D9S154	123.0	118,380,667–118,380,844	2.62	4.37×10^−3^	0.99
D9S1802	123.3	118,663,193–118,663,409	2.67	3.80×10^−3^	0.98
D9S1811	123.9	119,261,235–119,261,616	2.67	3.79×10^−3^	0.98
D9S1864	125.4	119,526,784–119,527,053	2.52	5.81×10^−3^	0.98
D9S275	126.6	120,626,197–120,626,394	2.43	7.58×10^−3^	0.97
D9S1872	128.4	120,829,322–120,829,427	2.29	1.11×10^−2^	0.99
D9S195	128.4	121,169,236–121,169,435	2.43	7.65×10^−3^	0.99
**D9S1682**	130.9	124,033,006–124,033,207	2.60	4.67×10^−3^	0.96
D9S1881	134.5	126,019,300–126,019,529	3.07	1.09×10^−3^	0.98
D9S1825	135.1	126,927,953–126,928,083	3.03	1.24×10^−3^	0.99
D9S1829	135.2	127,814,464–127,814,679	3.03	1.23×10^−3^	0.98
D9S1798	135.8	128,212,217–128,212,457	2.96	1.54×10^−3^	0.98
D9S1821	136.2	128,369,402–128,369,573	2.97	1.47×10^−3^	0.98
D9S112	136.8	128,413,136–128,413,266	2.91	1.80×10^−3^	0.97

Analysis was carried out in 149 independent families with multiple cases of spondyloarthritis. Three markers previously studied in a subset of 120 of these families appear in bold [13]. Markers were placed according to ENSEMBL database. Zlr statistics were calculated by multipoint analysis with ALLEGRO program. Markers within frames are those for which positive linkage was detected with *P*<0.05, after correction for multiple testing.

### Linkage disequilibrium (LD) mapping

**Figure 3:**
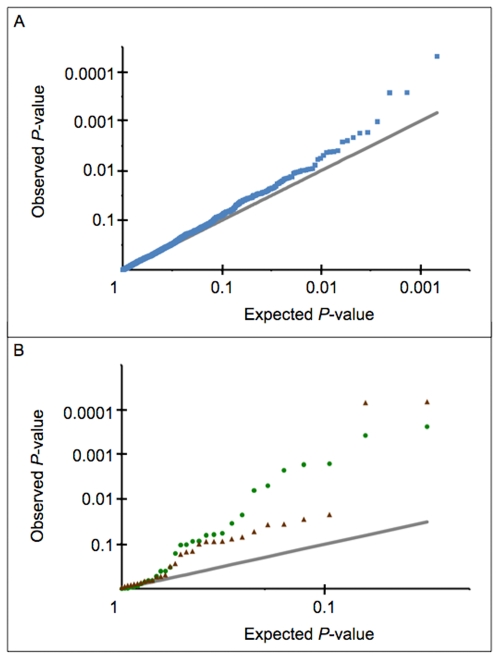
Quantile-quantile (Q-Q) plots comparing the distributions of observed versus expected *P*-values. (A) shows the LD mapping Q-Q plot. The blue squares represent the observed *P*-values. The grey line represents the null hypothesis of no true association. (B) shows the extension study mapping Q-Q plot. The green circles and the red triangles represent the observed *P*-values for the family-based and case/control studies respectively. The grey line represents the null hypothesis of no true association.

In the second part of our study we performed a linkage disequilibrium (LD) mapping of the 13.1 Mb region selected after the linkage fine mapping, using a family-based association test. We employed a tag SNP strategy that consisted of genotyping a set of 1,536 markers, extensively representative of genetic variability at the chromosomal region, in a sample of 136 families (Table 1). The sample was composed of 36 families with the highest linkage values in the selected 13.1 Mb region, and 100 novel families never tested before for either linkage or association (Figure 1). Among the 1,536 tag SNPs genotyped, 1,489 (96.9%) were suitable for family-based association testing. The remaining 47 tag SNPs were discarded from the analysis for genotyping failure or lack of polymorphism across the sample. Association analysis was performed using a transmission disequilibrium test (TDT) adapted for families larger than trios, and suitable for testing of association in the area of known linkage [24]. Considering the number of tag SNPs tested, applying crude Bonferroni correction would set the nominal *P*-value corresponding to a global type I error of 5% at 3.4×10^−5^. However such threshold is overly conservative, since some of the 1,489 tag SNPs presented a weak level of LD and were therefore not totally independent. To date, there is no consensus on the best method to take into account the non independence between SNPs. A method such as that proposed by Nyholt [25], would set the nominal 5% threshold at *P* = 5.56×10^−5^. Alternatively, accepting a corrected global type I error of 7.5% with Bonferroni would set the nominal threshold at *P* = 5×10^−5^. Using such criteria, one single intergenic tag SNP, rs4979459, was found to be significantly associated, with SpA (*P* = 4.9×10^−5^, Figure 2A, Table S1). Suggestive association was also found for several additional markers in LD with rs4979459 (Figure 2B, Table S1). The evidence of association for these SNPs was also supported by the comparison of observed and expected distributions of association *P*-values (Figure 3A). The distribution of observed *P*-values was very suggestively skewed from the null distribution with 23 SNPs having *P*<0.01, versus 14 expected under the null hypothesis. All SNPs demonstrating significant or suggestive association were located within an 80 kb LD block downstream from the *TNFSF15* gene (Figure 2A and 2C). There was no other region in the SPA2 locus presenting significant or even suggestive association with SpA. Notably the *TRAF1-C5* locus previously identified as associated with rheumatoid arthritis did not show any association with SpA in our investigation (Figure 2A).

### Extension family-based and case/control association study

To refine the association signal identified by the LD mapping stage we genotyped the tag SNP rs4979459 and an additional panel of 30 surrounding SNPs in an extended sample of 287 families comprising 668 SpA patients (including the 149 families of the linkage fine mapping, the 100 families added for the LD mapping, and 38 additional families (Table 1, Figure 1B)), as well as in an independent set of 139 cases and 163 controls (Figure 1A and 1B). Association was investigated by the TDT described above for the family sample and by an allelic chi-square test for the case/control set. In keeping with the LD mapping stage, we used the Bonferroni correction for multiple testing. The nominal *P*-values to achieve global type I errors of 5% and 7.5% significance were 1.61×10^−3^ and 2.42×10^−3^ respectively. Of note, the Nyholt correction set the nominal 5% threshold at *P* = 3.93×10^−3^.

In the family-based association study, the 5% Bonferroni corrected significance threshold was reached for 3 SNPs: rs10817669, rs10739427, and rs10759734, and the 7.5% significance threshold for 2 additional SNPs: rs10733612, and rs7849556 (Table 3, Table S2). Several other markers including the tag SNP rs4979459 yielded low *P*-values (Table S2). For all these SNPs the major allele was overtransmitted to affected children.

**Table 3:** Results of family-based and case/control association studies for eight SPA2 single-nucleotide polymorphisms (SNPs).^a^

SNP	Position (pb)	Minor allele	Major allele	Family Extension Results (287 Families)			Case/Control Extension Results (139 Cases/163 Controls)				Case/Control Replication Results (232 cases/149 controls)				Pooled Case/Control Results (371 cases/312 controls)				Combined Results
				Number of informative pedigrees	OT allele^a^	*P*-value FBAT	Minor Allele Frequency		OR	*P* b	Minor Allele Frequency		OR	*P* b	Minor Allele Frequency		OR (95% CI)	*P* b	*P* c
							Cases	Controls			Cases	Controls			Cases	Controls			
rs4979459	116,521,487	G	T	140	T	0.005	0.454	0.500	0.83	0.274	0.429	0.463	0.87	0.354	0.438	0.482	0.84 (0.67–1.04)	0.108	9×10^−04^
rs7849556	116,522,493	C	A	112	A	0.002	0.201	0.266	0.69	0.087	0.197	0.236	0.79	0.233	0.198	0.250	0.74 (0.56–0.99)	0.040	4×10^−04^
rs10817669	116,522,717	G	A	131	A	2×10^−04^	0.271	0.326	0.77	0.150	0.248	0.258	0.95	0.765	0.257	0.300	0.82 (0.64–1.06)	0.132	1×10^−04^
rs10759734	116,536,471	G	A	128	A	0.002	0.195	0.272	0.65	0.028	0.211	0.242	0.84	0.325	0.205	0.257	0.75 (0.58–0.96)	0.023	1×10^−04^
rs6478105	116,557,006	G	A	88	A	0.064	0.063	0.171	0.33	7×10^−05^	0.106	0.160	0.62	0.029	0.090	0.166	0.50 (0.36–0.69)	3×10^−05^	3×10^−05^
rs10982396	116,558,750	G	C	86	C	0.162	0.064	0.173	0.33	7×10^−05^	0.109	0.149	0.71	0.114	0.092	0.161	0.53 (0.38–0.74)	2×10^−04^	3×10^−04^
rs10733612	116,562,871	T	C	123	C	0.002	0.195	0.270	0.66	0.036	0.202	0.240	0.80	0.260	0.199	0.256	0.72 (0.55–0.95)	0.018	1×10^−04^
rs4246905	116,593,070	T	C	130	C	0.058	0.254	0.341	0.66	0.022	0.302	0.312	0.95	0.762	0.284	0.327	0.82 (0.65–1.03)	0.090	0.010

a Refer to Figure 1 for the study design. OT: overtransmitted to affected children allele; OR: odds ratio; CI: confidence interval.

b Asymptotic *P*-values of the chi-square test for allele frequencies comparison between cases and controls.

c Asymptotic *P*-values for combined family and case/control samples computed with the Cochran-Mantel-Haenszel test.

**Figure 4:**
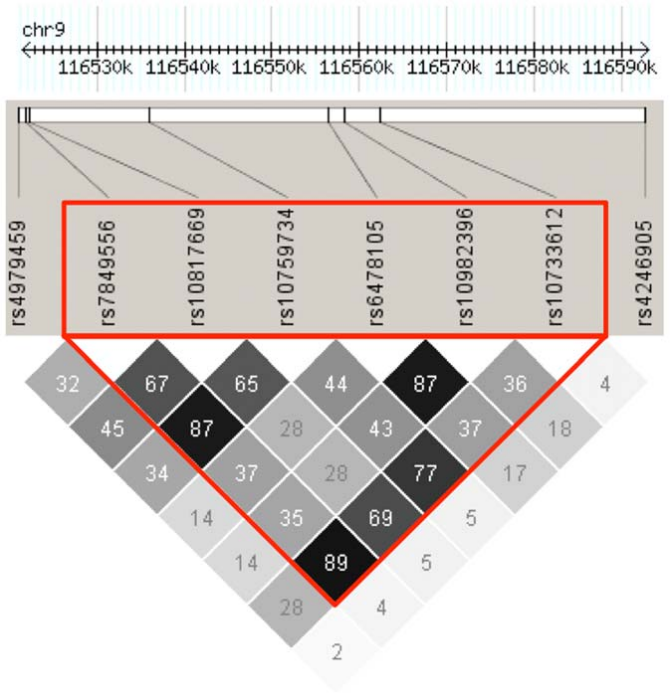
Linkage disequilibrium (LD) plot for the eight single-nucleotide polymorphisms genotyped in the replication study. LD calculations were based on pairwise r^2^ values of our entire case/control dataset. These values are displayed, in percentage, in the grey boxes. The red triangle indicates the LD block, computed by the program HAPLOVIEW, which was further used for haplotypic association with the program PLINK.

In the case/control study two markers, in strong LD with each other, reached a Bonferroni corrected 0.005 significance threshold: rs6478105 and rs10982396 (Table 3, Figure 4). Odds ratios (ORs)<1 were observed for both of them, indicating that the major allele was more frequent in cases than in controls. Other markers yielded non-significant low *P*-values (Table S3).

The evidence of association for these SNPs was also supported by the comparison of the observed and expected distributions of *P*-values for association (Figure 3B). The distribution of observed *P*-values was very suggestively skewed from the null distribution with 7 SNPs having *P*<0.01 in the family-based study and 2 in the case/control study, versus 0 expected under the null hypothesis.

All the markers significantly associated with SpA in all of our association studies belong to the same LD block (Figure 4).

The 5 SNPs located within the *TNFSF15* gene, which were previously found to be associated with CD [20],[21], did not reach a level of significant association in both the family-based design and the case/control study (Table S2 and Table S3).

### Replication case/control association study

To replicate our results in an independent sample, we genotyped a set of eight SNPs consisting of the most strongly associated markers in the foregoing extension study (rs7849556, rs10817669, rs10759734, rs6478105, rs1982396, and rs10733612), the tag SNP (rs4979459) and one SNP located in the neighboring *TNFSF15* gene (rs4246905) in an additional independent set of 232 cases and 149 controls (Figure 1, Table 3). Association was assessed using a chi-square test. After correction for multiple testing, one SNP, rs6478105, reached a suggestive association level (nominal *P* = 0.029), the Bonferroni corrected for multiple testing thresholds being 0.0063 (5%) and 0.0094 (7.5%).

Of note, OR<1 was observed for all eight SNPs. This trend was similar to that observed in the whole extension step of the study, for both family-based and case/control approaches (see above), suggesting that even if the *P*-value for significance was not reached here, the direction of association was consistent between both studies for all markers.

### Pooled case/control analyses

The entire case/control sample, comprising the “extension” and the “replication” part (371 cases and 312 controls) was actually an independent set, as compared to the family sample (Figure 1). Thus, it made sense to perform an association analysis on this “pooled” sample. Results of this analysis are displayed in the Table 3. Significant association level was reached for two of the eight tested SNPs: rs6478105 (*P* = 3×10^−5^; OR = 0.5) and rs10982396 (*P* = 2×10^−4^; OR = 0.53). Furthermore, three other SNPs presented suggestive association with low *P*-values (rs7849556, rs10759734, and rs10733612). Notably, HLA-B27 conditioned exploratory analyses showed exactly the same trend of allelic distribution between cases and controls, suggesting that the detected association signal was independent of the presence of HLA-B27 (Table S4 and Table S5).

### Combined results

We also performed combined analysis of the genotyping issued from both the family extension sample and either the pooled case/control set or the extension case/control set for the same eight SNPs. The tests were performed using the Cochran-Mantel-Haenszel method [26] (Table 3 and Table S6, respectively). These investigations led us to confirm the strong association with SpA of the whole LD block containing these SNPs (Figure 4).

Of note the non-synonymous SNP, rs4246905, which changes a histidine into arginine, located in the fourth exon of *TNFSF15* gene and previously identified as strongly associated with CD [20],[21] reached a suggestive *P*-value of less than 0.01 in both combined analyses. Nonetheless, this was by far the weakest association of the eight tested markers.

### Family-based and case/control haplotypic analyses

The six SNPs presenting the lowest *P*-values after the combined analysis were located in the same strong LD block (Figure 4). It is known that in the presence of multiple tightly linked markers a haplotype test may be more powerful to detect association.

Association results for the haplotypes comprising these six SNPs are displayed in the Table 4, together with their estimated frequencies.

**Table 4:** Results of family-based and case/control haplotype association analyses for the most associated LD block.^a^

Haplotype allele name	SNP alleles^b^						Extension family-based study (287 families 1,578 individuals, 668 SpA patients)				Pooled case/control study (371 SpA cases/312 controls)		
	1	2	3	4	5	6	Family frequency	Number of informative pedigrees	Z	FBAT *P*-value	Frequency		*P*-value
											Cases	Controls	
	- OMNIBUS -						/	127	25.26	4.5×10^−5^	/	/	7.02×10^−4^
H1	A	A	A	A	C	C	0.714	116	4.45	8.81×10^−6^	0.75	0.71	0.095
H2	C	G	G	G	G	T	0.131	67	−1.11	0.267	0.08	0.15	8.75×10^−5^
H3	C	G	G	A	C	T	0.094	67	−3.15	0.002	0.10	0.09	0.613
H4	A	G	A	A	C	C	0.037	20	−1.84	0.066	0.06	0.04	0.110

a SNP: single nucleotide polymorphism.

b The tested haplotype consisted of the 6 following SNPs: rs7849556 (**1**); rs10817669 (**2**); rs10759734 (**3**); rs6478105 (**4**); rs10982396 (**5**); rs10733612 (**6**).

Results are shown for the omnibus haplotype tests and for the four individual haplotypes called H1 to H4, with an allele frequency high enough to allow meaningful statistical tests. These represented 99% of the alleles. For the family-based study, the number of analyzed families, large sample FBAT statistic (Z), and the association *P*-value are shown. A positive Z means that the concerned allele was found as overtransmitted to affected offspring; a negative Z conversely indicates an undertransmission. When the omnibus haplotype association is concerned the “Z” symbolizes the chi-square statistic. For this type of test the number of degrees-of-freedom (df) was 4. For case/control analyses the frequency of each haplotype-allele in cases and in controls, as well as the association *P*-value are displayed. The number of df for the omnibus association was 3.

The family-based haplotype association analysis identified the most frequent allele H1 as overtransmitted to affected children with a high significance level (Table 4; *P* = 8.81×10^−6^), the other three alleles being undertransmitted. The significance threshold corrected for the multiplicity of tested haplotypes and extrapolated with Bonferroni method was set to 0.013.

The case/control haplotype association analysis conversely showed that the rare haplotype H2 was very significantly more frequent in controls than in cases (Table 4; *P* = 8.75×10^−5^), with all the other haplotypes being more frequent in cases than in controls. Case/control HLA-B27 conditioned analyses showed that the frequency of H2 haplotype was significantly decreased in patients, independently of the presence of HLA-B27 (Table S4 and Table S5).

A significant omnibus haplotype association was detected in family-based sample (*P* = 4.5×10^−5^ at 4 degrees-of-freedom (df)), as well as in the case/control one (*P* = 7.0×10^−4^ at 3 df).

## Discussion

We report for the first time an association between several SNPs determining a haplotype near the *TNFSF15* gene (9q32) and SpA. This was achieved by a comprehensive study with several linkage and association steps.

The basis of our investigations aimed to narrow down from our previous whole genome linkage screen the susceptibility region for SpA on chromosome 9q31–34 called SPA2 [13]. Since initial linkage in this locus was spread over a long distance of 23.95 cM, our first attempt was to refine it using a fine-grained set of 28 microsatellite markers in an extended set of families. The results revealed two areas of statistically significant linkage, with the highest linkage peak located on the marker D9S1824 at 120.1 cM (115.9 Mb) from the p-telomere, only 1.3 cM apart from D9S1776, which corresponded to the linkage peak in our former screen. The second statistically significant area was located near a suggestive linkage peak reported in AS (D9S1682, *P* = 6×10^−4^) [12], supporting the validity of linkage between this region and SpA. Since non parametric linkage analyses usually do not characterize linkage localization with high precision [27], we decided to continue our investigations on the 13.1 Mb region between markers D9S279 and D9S112 comprising both significantly linked areas.

To identify whether one or several loci of the refined SPA2 region were associated with SpA we performed a family-based dense LD mapping of this 13.1 Mb area, using a tag SNP strategy. Our sample set was enriched in families with strong evidence of linkage, in order to minimize the risk of false negative results. After correction for multiple testing, significant association was observed for one single tag SNP, rs4979459 (*P* = 4×10^−5^).

Several arguments support the assumption that this is a true positive finding. Firstly, suggestive association was also found for several additional markers in LD with rs4979459, indicating that the identified significant association was unlikely to be explained by systematic genotyping error. Secondly, the use of a family-based design rules out the possible confounding effect of population stratification. Finally, rs4979459 was located in the direct vicinity of our highest linkage peak, between the microsatellite markers D9S279 and D9S1855.

None of the genes having a counterpart in the MHC, i.e. those that are theoretically good candidates for disease susceptibility, were shown to be associated with the disease in this study. Notably, the *TRAF1-C5* locus which lies at 122.7 Mb from the p-telomere and was recently described as associated with rheumatoid arthritis [22],[23], did not show any association with SpA in our study.

To refine the association pointed out by our LD map, we performed an extension study with 31 SNPs. Characteristically, the initial strong association observed with rs4979459, was not as strong in the extension stage, suggesting that rs4979459 is not the causal variant. Its effect was probably overestimated in the LD mapping step, since initial positive reports tend to overestimate found effects, while subsequent studies regress to the true value [28]. Moreover, SpA is a complex disease with known genetic heterogeneity, and therefore enriching the LD mapping family set with strongly linked families is also likely to have contributed to this overestimation.

After extension and replication steps of our study, strong association was seen in the family sample as well as in the entire case/control set with SNPs located in the strong 40.3 kb LD block showed in the Figure 4. The markers that were found to be associated by these two approaches were not exactly the same. Several reasons could readily explain such an apparent discrepancy. First of all, the alleles of rs6478105 and rs10982396, which were found to be associated with SpA in the pooled case/control sample, were highly frequent (>0.89). Therefore there were a relatively modest number of informative families for these markers in the family-based study, since at least one parent has to be heterozygous to render the family suitable for association analysis. On the other hand, the power of our case/control study was much lower to demonstrate association with rs10817669 (the SNP significantly associated in the family-based study (OR of 0.82)), than with rs6478105 and rs10982396 (OR of 0.50, and 0.53, respectively).

Nevertheless, even if the significance level was not reached in every study for every marker (probably attributable to a lack of power), we can see that in three totally independent samples trends for all SNPs were exactly the same: the frequent allele of each marker was overtransmitted to affected children and noticeably more present in cases rather than in controls. Moreover the results of combined analysis of family and case/control samples also strongly support the association with all six SNPs, composing the 40.3 kb LD block. Finally, our haplotype investigations also confirmed this trend and showed that haplotypes composed of markers of this block were very significantly associated with the disease, for both family-based and case/control investigations.

The high risk haplotype (H1) identified in our study was too frequent to explain the strong linkage signal detected in SPA2 [29], and was likely only a surrogate for the causal variant(s). As the LD block containing this haplotype is located 28.6 kb from the *TNFSF15* gene, one of the best candidate genes in the region, in particular because of its implication in CD [20],[21], it made sense to test this gene directly. However, our LD mapping stage data did not implicate the *TNFSF15* “strictly genic” region (introns and exons). Indeed, no tag SNP association was identified in this region and none of the five SNPs described as associated with CD were associated with SpA in our analyses. A full re-sequencing of all exons, 1st and 3rd introns, and additional intronic boundaries of the *TNFSF15* gene in several patients and controls did not reveal unmatched variations either (data not shown).

We were also well aware of the limits presented by our extension/replication approach, which was focused only on the “LD mapping” of an association peak. Thus, we tested by a classic candidate-gene approach several genes located within the region surrounding the linkage peaks. We first performed variants screening in a group of independent SpA patients from families presenting a high linkage signal within the studied region, and unrelated controls. The most suggestive polymorphisms were subsequently genotyped. The association with the disease was therefore either assessed with TDT in a sample of independent trios or with a chi-square test in a large case/control sample. In this way, we tested the implication of Tenascine-Cytoactine gene (*TNC*), coding for an extracellular matrix glycoprotein and presenting a paralogous counterpart in the MHC class III locus. No association was found between polymorphisms in this gene and SpA [30]. We also performed a more systematic candidate-gene approach, testing among others Tumor necrosis factor ligand superfamily member 8 - CD30 ligand (*TNFSF8*), Zinc finger protein 618 (*ZNF618*), Kinesin-like protein (*KIF12*), Alpha-1-acid glycoprotein 1 Precursor (*ORM1*), Alpha-1-acid glycoprotein 2 Precursor (*ORM2*) and alpha-1-microglobulin/bikunin precursor (*AMBP*) genes. The implication in the disease of polymorphisms tested within these genes was excluded by this approach, corroborating the results of our LD mapping, presented in this article (Zinovieva E *et al.* manuscript in preparation).

Despite the fact that SNPs within the *TNFSF15* coding region were excluded by our combined tag SNP and candidate-gene approach, it is still possible that polymorphisms identified by the H1 haplotype play a role in the regulation of this gene. *TNFSF15* belongs to the TNF superfamily of genes, otherwise implicated in SpA [31] and is specifically implicated in gut inflammation, a frequent SpA manifestation [32],[33]. Its product, TNFSF15, also called TLA1, is implicated in the modulation of T-helper 17 lymphocytes activation [32], the number of which has been shown to be increased in patients with SpA [34]. Finally a recent study reported that polymorphisms within *TNFSF15* to be associated with CD are playing a role in the transcriptional regulation of the gene [35]. This report compounds with our hypothesis, since our findings could be consistent with an indirect role of *TNFSF15* in SpA. However, whether the causal variant(s) tagged by the six SNPs haplotype is(are) related to TNFSF15 function and/or regulation or to any other gene in the SPA2 region will require further investigations.

## Materials and Methods

### Ethics statement

This study was approved by the institutional ethics committee of Cochin Hospital (Paris, France) and of Ambroise Paré Hospital (Boulogne-Billancourt, France), and written informed consent was obtained from each participant.

### Subjects

Caucasian families consisting of one or several cases of SpA and additional parents were recruited throughout France by the “Groupe Français d'Etude Génétique des Spondylarthropathies” (GFEGS). In case/control panels, independent cases were recruited through the Rheumatology clinic of Ambroise Paré Hospital (Boulogne-Billancourt), or through the national self-help patients' organization: “Association Française des Spondylarthritiques”. Independent controls were obtained from the “Centre d'Etude du Polymorphisme Humain”, or were recruited as healthy spouses of cases with either no children or no affected children.

The phenotypic description of patients from familial and case/control samples is shown in Table 5. The diagnosis of SpA was made according to the classification criteria of Amor et al [36] and/or the European Spondylarthropathy Study Group (ESSG) [37]. Within the group of SpA, AS was diagnosed according to the modified New York criteria [38]. Regarding extra-articular manifestations, the diagnosis of psoriasis required the presence of typical lesions and/or a clinical diagnosis established by a dermatologist. The diagnosis of anterior uveitis required examination by an ophthalmologist. Inflammatory bowel disease diagnosis (including CD and ulcerative colitis) was based on endoscopic and histological examination of the gut. ReA was diagnosed according to the criteria published by Willkens [39]. Finally, uSpA was diagnosed when SpA criteria were fulfilled, without any of the foregoing diagnosis.

**Table 5:** Clinical characteristics of patients with spondyloarthritis included in the whole study.^a^

Characteristics	Familial patients (n = 711)	Singleton cases (n = 371)
Age in year, mean±SD	44±0.56	46±0.70
Age at onset, in year, mean±SD	24±0.36	26±0.60
Sex ratio, men∶women	383∶328	177∶194
*HLA-B27* positivity^b^	93%	77%
*Axial manifestations*		
Back/buttock pain	97%	97%
Sacroiliitis (radiography)^c^	58%	50%
*Peripheral manifestations*		
Arthritis	43%	35%
Enthesistis	66%	80%
*Extra-articular manifestations*		
Uveitis	29%	23%
Psoriasis	25%	29%
Inflammatory bowel disease	7%	7%
*Spondyloarthritis subtype*		
AS	58.30%	48.03%
uSpA	29.66%	34.05%
PsA	8.96%	16.13%
AIBD	2.64%	3.23%
ReA	0.44%	/

a The registered manifestations correspond to those present at time of examination, or retrieved from past-medical history. Inflammatory bowel disease: Crohn's disease or ulcerative colitis; AS: ankylosing spondylitis, uSpA: undifferentiated spondyloarthritis; PsA: psoriatic arthritis; AIBD: inflammatory bowel disease-associated arthritis; ReA: reactive arthritis.

b The number of patients evaluated in each group was 710 and 327 respectively.

c Refers to radiographic sacroiliitis≥grade II bilateral or grade III unilateral. The number of patients evaluated in each group was 643 and 282 respectively.

### DNA isolation and HLA-B typing

Genomic DNA was extracted from peripheral blood using standard methods. HLA-B typing was routinely performed by a polymerase chain reaction (PCR) - based sequence-specific method [40]. For individuals already typed as positive for *HLA-B27*, retyping was not routinely performed.

### Genotyping

#### Linkage fine mapping

The fine mapping markers panel consisted of 28 microsatellites with maximal level of heterozygosity, regularly spaced over the SPA2 region, between D9S1677 and D9S112 (average marker spacing 0.89 cM and mean heterozygosity of 0.76). Markers were selected from the University of California Santa Cruz (UCSC) public database. They included 3 of the 5 microsatellites previously studied in our former screen [13], and 25 additional markers (Table 2, Figure 1). Typing of microsatellites was performed at the French National Genotyping Center (CNG, Evry, France) on a set of 149 multiplex families (Figure 1, Table 1). Multiplex PCRs were performed using fluorescently-labeled primers (FAM, HEX and NED) (Applied Biosystems, Foster City, CA). Amplimers generated by PCRs were loaded on a MegaBACE1000 (GE Healthcare, Chalfont St. Giles, UK) and allele calling of the fragments was performed with the GeneticProfiler software (Amersham Biosciences - GE Healthcare).

#### LD mapping

A customized chip containing 1,536 tag SNPs was designed using resources provided by the HapMap project [41]. Tag SNP selection aimed at covering almost all the common variations (r^2^>0.8; more than 1 tag SNP/10 kb) of the 13.23 Mb target region, which was selected following the linkage fine mapping stage. High-throughput genotyping was performed on a customized Illumina BeadChips using the GoldenGate assay at the CNG. A detailed protocol for this assay is described by Illumina on their web site. The GoldenGate reaction is based on allele specific extension and universal PCRs at 1,536 targets [42]. After amplification the GoldenGate assay products were hybridized on a Sentrix 96Array matrix [43], a fiber-optic gene array [44],[45], and washed prior to being analyzed by fluorescence. The call rate obtained was ≥0.99. Patients DNA genotypes in 136 families by the mean of this methodology were investigated (Figure 1, Table 1).

#### Extension association study

After the tag SNP screen described above was achieved, 25 SNPs were further selected through the HapMap and dbSNP NCBI databases according to the following criteria:

- The selected markers had a minor allele frequency (MAF) ≥0.1 in Caucasian populations, and were located nearby the tag SNP previously identified to be associated with SpA (up to a maximum of 30 kb upstream and of 106 kb downstream). These markers were also as far as possible in strong LD (D' = 1; strong r^2^) with the associated tag SNP. We genotyped 5 more additional SNPs within the *TNFSF15* gene (rs4246905, rs6478108, rs7030574, rs6478109, and rs7848647), being the closest gene located in the vicinity of the peak of association. Of note, these 5 SNPs were chosen because they have been previously identified as associated with CD [20],[21]. In all, 31 SNPs were genotyped in this extension part of the study, including the tag SNP previously identified as associated with SpA. Genotyping was carried out at the CNG using TaqMan (assay-by-design) according to the manufacturer's recommendations with probes and Mastermix from Applied Biosystems (Courtaboeuf, France). End point fluorescence was detected using an ABI7900HT reader (Applied Biosystems). Genotypes were assigned with the SDS 2.1 software. Investigations were carried out on a set made of 287 families (Table 1) as well as on an independent sample of 139 cases and 163 controls (Figure 1).

#### Replication study

Finally, a replication study focused on 8 SNPs located in the reduced 71.6 kb region was performed on a new case/control sample composed of 232 SpA patients and 149 controls. Genotyping was put through a production-scale 48-plex (SNPlex) assay (Applied Biosystems, Courtaboeuf, France) [46],[47]. Automatic allele assignment was achieved with the GeneMapper software v4.0 (Applied Biosystems), with the rules-clustering algorithm.

#### Verification of SNPs genotyping

In the different stages of the study, several individuals have been genotyped by two or three different technologies (Illumina, Taqman, or SNPlex) for several SNPs. The level of genotyping concordance was set to 95%. For SNPs that did not reach this threshold (rs4979459, rs10759734, rs6478105, rs10982396, and rs4246905) an alternative method of genotyping was used in order to resolve the correct genotype. In this way, 436 individuals were genotyped using melting curve analyses (LightCycler System, Roche, Meylan, France). Both PCR primers and hybridization probes were synthesized by Tib MolBiol (Berlin, Germany). All observed discrepancies were solved.

### Statistical analysis

For familial studies, Mendelian inheritance inconsistencies were identified with the PEDCHECK program [48]. PLINK program [49] was used to assess the deviation from Hardy-Weinberg equilibrium in unrelated subjects. Family pairwise distributions among first and second degree relative pairs were accounted for with the PEDSTATS program [50].

For the fine mapping linkage study, allele frequencies were estimated using MENDEL software [51]. Evidence for linkage was assessed using Zlr statistic [52] based on S_pairs_ with the exponential model and multipoint identity-by-descent computation using ALLEGRO program [53]. *P*-values were computed on the basis of large-sample theory; the distribution of Zlr statistic approximates a standard normal random variable under the null hypothesis [52],[53]. Whole fine map significance was extrapolated using Bonferroni correction for 28 tests.

Family-based allelic single-locus association analyses (1,536 tag SNP in the 136 families of the LD mapping and 31 SNPs in the 287 families of the extension study (Figure 1)) were carried out using FBAT [54]. FBAT is a flexible program appropriate for analyses of family data larger than trios, allowing association tests that are robust to population cofounds in the case where parental data are missing and/or other offspring are included in the analysis [55]. We specified the option to calculate the variance empirically (“-e” option) in order to provide valid tests of association in the presence of linkage [24]. The global significance threshold for each set of SNPs was assessed using Bonferroni correction.

It has been shown that in some situations (such as when r^2^ factor between the risk variant and a particular multi-SNP haplotype is very strong) haplotypes may provide more information for association than corresponding single-locus tests [56]. HBAT is an elaboration of FBAT that allows family-based association tests of haplotypes, even when the phasing is ambiguous. Family-based haplotype-specific association in the presence of known linkage was assessed with the “hbat -e” option of FBAT program, for a set of pre-selected tightly linked markers. This option allows one to perform two types of haplotype tests. In the first type, each haplotype allele is tested for association against all the others using a one degree-of-freedom (df) test. Significance of these tests must be extrapolated with a multiple testing correction; here we used the Bonferroni method. The second type of haplotype tests is a global multiallelic test with several df. In this case, there is no need to correct for multiple testing.

Case/control association studies were carried out using the standard chi-square test comparing allelic frequencies between cases and controls and giving asymptotic *P*-values, implemented in PLINK package [49]. Allele frequencies, ORs, and their 95% confidence intervals were also estimated using this software. When needed, the adjustment for multiple testing was performed using the Bonferroni correction.

The quantile-quantile (Q-Q) plots (Figure 3) were constructed by ranking the sets of association *P*-values from the largest to the smallest and plotting them against their expected values. Under the null hypothesis the expected *P*-value for the *i*th SNP is *i*/*n*, where *n* is the total number of tested markers.

Haplotype-specific association was assessed in case/control samples for a set of pre-selected tightly linked SNPs (the same as for the family investigation described above) with the “hap-assoc” option of PLINK. This procedure takes into account the uncertainty of haplotype phase and performs both one df chi-square haplotype-specific tests, which significance must be extrapolated with a multiple testing correction, here Bonferroni correction, and an omnibus association statistic considering all the haplotypes.

The explorative combined analysis of the whole association data from the extension and replication studies (1 family sample and 2 case/control samples; Figure 1) was performed with the “dfam” option of PLINK. This particular test implements a sib-TDT [57] for nuclear families, to include sibships without parents as well as unrelated individuals and assesses the association via a clustered-analysis using the Cochran-Mantel-Haenszel test [26]. It does not take into account the presence of linkage in the region.

LD plots were constructed using HAPLOVIEW program [58].

### Web resources

The URLs for data presented herein are as follows:

* Association Française de Spondylarthritiques (AFS): http://www.spondylarthrite.org/

* Centre d'Etude du Polymorphisme Humain (CEPH): http://www.cephb.fr/

* University of California Santa Cruz (UCSC) public database: http://genome.ucsc.edu/

* French National Genotyping Center (CNG): http://www.cng.fr/

* Detailed protocol for the Illumina BeadChips GoldenGate assay: http://icom.illumina.com/General/pdf/LinkageIV/GOLDENGATE_ASSAY_FINAL.pdf

* Ensembl database: http://www.ensembl.org/index.html

* NCBI dbSNP database: http://www.ncbi.nlm.nih.gov/projects/SNP/

* HapMap database: http://www.hapmap.org/

* PEDCHECK program: http://watson.hgen.pitt.edu/register/docs/pedcheck.html

* MENDEL program: http://www.genetics.ucla.edu/software/mendel

* ALLEGRO program: http://www.decode.com/software/

* FBAT program: http://www.biostat.harvard.edu/~fbat/fbat.htm

* PEDSTATS program: http://www.sph.umich.edu/csg/abecasis/PedStats/index.html

* PLINK program: http://pngu.mgh.harvard.edu/~purcell/plink/

* HAPLOVIEW program: http://www.broad.mit.edu/mpg/haploview/

## Supporting Information

Table S1 Complete results of the family-based association linkage disequilibrium mapping study (Illumina BeadChip genotyping - 136 families).
(1.74 MB DOC)

Table S2 Complete results of the family-based association extension study (TaqMan genotyping - 287 families).
(0.08 MB DOC)

Table S3 Complete results of case/control association extension study (TaqMan genotyping - 139 SpA cases/163 healthy controls).
(0.07 MB DOC)

Table S4 Single SNP (1), and haplotype-based (2) association results in HLA-B27 positive case/control sample (261 SpA cases/27 healthy controls).
(0.05 MB DOC)

Table S5 Single SNP (1), and haplotype-based (2) association results in HLA-B27 negative case/control sample (73 SpA cases/255 healthy controls).
(0.05 MB DOC)

Table S6 Results of combined family-based and case/control association-extension studies for eight SPA2 single-nucleotide polymorphisms (SNPs).
(0.04 MB DOC)
