# Supplementary material for: Comprehensive Linkage and Association Analyses Identify Haplotype, Near to the TNFSF15 Gene, Significantly Associated with Spondyloarthritis
Source: PLoS Genet. 2009 Jun 19;5(6):e1000528. doi: 10.1371/journal.pgen.1000528 (PMC2689651; doi:10.1371/journal.pgen.1000528)
Supplement: Table S1 — Complete results of the family-based association linkage disequilibrium mapping study (Illumina BeadChip genotyping - 136 families). (1.74 MB DOC) [file pgen.1000528.s001.doc]

**Table S1**. Complete results of the family-based association linkage disequilibrium mapping study (Illumina BeadChip genotyping -136 families).

| Marker name | OT  allele frequency^a^ | Number of informative pedigrees | Z^b^ | Nominal  *P*-value^c^ | Position (bp) |
| --- | --- | --- | --- | --- | --- |
| rs10981794 | 0.85 | 44 | 0.71 | 4.80x10^-01^ | 115,280,066 |
| rs6478011 | 0.90 | 31 | 1.24 | 2.16x10^-01^ | 115,284,803 |
| rs7851937 | 0.79 | 54 | 1.02 | 3.08x10^-01^ | 115,344,125 |
| rs4979253 | 0.42 | 73 | 1.18 | 2.39x10^-01^ | 115,354,739 |
| rs883025 | 0.84 | 41 | 1.88 | 6.06x10^-02^ | 115,365,320 |
| rs3747813 | 0.94 | 17 | 0.78 | 4.33x10^-01^ | 115,367,281 |
| rs4979257 | 0.81 | 52 | 0.59 | 5.58x10^-01^ | 115,374,598 |
| rs7875727 | 0.20 | 52 | 2.23 | 2.61x10^-02^ | 115,378,030 |
| rs4327927 | 0.69 | 63 | 0.08 | 9.38x10^-01^ | 115,378,963 |
| rs518636 | 0.31 | 70 | 0.20 | 8.42x10^-01^ | 115,402,967 |
| rs585689 | 0.65 | 70 | 0.10 | 9.23x10^-01^ | 115,406,009 |
| rs2774638 | 0.48 | 76 | 1.46 | 1.44x10^-01^ | 115,410,599 |
| rs505668 | 0.35 | 68 | 1.24 | 2.14x10^-01^ | 115,422,202 |
| rs538711 | 0.25 | 59 | 0.68 | 4.97x10^-01^ | 115,427,286 |
| rs2127744 | 0.26 | 55 | 1.17 | 2.43x10^-01^ | 115,430,890 |
| rs2127745 | 0.89 | 40 | 0.04 | 9.72x10^-01^ | 115,430,983 |
| rs589248 | 0.14 | 37 | 0.44 | 6.63x10^-01^ | 115,437,218 |
| rs2812401 | 0.72 | 64 | 0.88 | 3.77x10^-01^ | 115,445,389 |
| rs67561 | 0.70 | 57 | 0.80 | 4.22x10^-01^ | 115,448,459 |
| rs681218 | 0.76 | 59 | 0.68 | 4.94x10^-01^ | 115,451,237 |
| rs10817497 | 0.91 | 28 | 0.27 | 7.85x10^-01^ | 115,453,572 |
| rs10981863 | 0.50 | 65 | 0.22 | 8.29x10^-01^ | 115,456,126 |
| rs2622266 | 0.82 | 47 | 0.55 | 5.81x10^-01^ | 115,460,067 |
| rs7854061 | 0.67 | 64 | 0.65 | 5.17x10^-01^ | 115,465,681 |
| rs784935 | 0.85 | 40 | 0.18 | 8.58x10^-01^ | 115,474,167 |
| rs784932 | 0.61 | 56 | 0.03 | 9.77x10^-01^ | 115,475,545 |
| rs784933 | 0.46 | 68 | 1.42 | 1.56x10^-01^ | 115,475,907 |
| rs3860173 | 0.26 | 47 | 0.85 | 3.94x10^-01^ | 115,476,698 |
| rs700123 | 0.96 | 10 | 0.82 | 4.14x10^-01^ | 115,478,920 |
| rs7852392 | 0.14 | 35 | 0.60 | 5.50x10^-01^ | 115,479,570 |
| rs4979274 | 0.89 | 35 | 0.47 | 6.42x10^-01^ | 115,484,090 |
| rs3904317 | 0.84 | 44 | 0.62 | 5.36x10^-01^ | 115,486,702 |
| rs586348 | 0.50 | 67 | 0.02 | 9.81x10^-01^ | 115,487,544 |
| rs7855534 | 0.34 | 60 | 0.78 | 4.36x10^-01^ | 115,492,822 |
| rs10122001 | 0.30 | 54 | 0.94 | 3.46x10^-01^ | 115,494,258 |
| rs10513208 | 0.06 | 18 | 0.00 | 1.00x10+00 | 115,498,232 |
| rs2170226 | 0.18 | 47 | 0.33 | 7.40x10^-01^ | 115,503,981 |
| rs1556263 | 0.47 | 66 | 1.51 | 1.31x10^-01^ | 115,504,428 |
| rs10123403 | 0.44 | 67 | 1.27 | 2.05x10^-01^ | 115,508,506 |
| rs987498 | 0.04 | 17 | 0.00 | 1.00x10+00 | 115,527,278 |
| rs10981904 | 0.39 | 58 | 0.96 | 3.39x10^-01^ | 115,531,750 |
| rs7859191 | 0.26 | 59 | 0.62 | 5.36x10^-01^ | 115,534,180 |
| rs10817516 | 0.87 | 36 | 0.17 | 8.62x10^-01^ | 115,538,105 |
| rs10817517 | 0.30 | 61 | 0.67 | 5.01x10^-01^ | 115,538,848 |
| rs1536328 | 0.58 | 64 | 0.81 | 4.16x10^-01^ | 115,554,697 |
| rs10981914 | 0.88 | 35 | 1.91 | 5.56x10^-02^ | 115,561,251 |
| rs7868736 | 0.28 | 67 | 1.59 | 1.11x10^-01^ | 115,568,004 |
| rs1994002 | 0.51 | 58 | 0.28 | 7.77x10^-01^ | 115,570,414 |
| rs1409382 | 0.52 | 61 | 0.49 | 6.28x10^-01^ | 115,578,652 |
| rs1022949 | 0.89 | 34 | 1.06 | 2.90x10^-01^ | 115,585,579 |
| rs4332200 | 0.33 | 66 | 0.84 | 4.00x10^-01^ | 115,592,809 |
| rs913078 | 0.91 | 29 | 0.46 | 6.44x10^-01^ | 115,596,320 |
| rs7037774 | 0.80 | 46 | 0.65 | 5.13x10^-01^ | 115,600,456 |
| rs10981955 | 0.95 | 13 | 1.15 | 2.51x10^-01^ | 115,631,293 |
| rs10759661 | 0.53 | 57 | 0.34 | 7.32x10^-01^ | 115,660,754 |
| rs10981981 | 0.64 | 62 | 0.20 | 8.39x10^-01^ | 115,667,378 |
| rs1330165 | 0.93 | 22 | 0.15 | 8.81x10^-01^ | 115,669,590 |
| rs4978557 | 0.44 | 69 | 0.54 | 5.89x10^-01^ | 115,675,311 |
| rs7861276 | 0.76 | 49 | 1.22 | 2.21x10^-01^ | 115,695,059 |
| rs7870363 | 0.63 | 65 | 1.36 | 1.73x10^-01^ | 115,705,393 |
| rs882980 | 0.49 | 54 | 1.40 | 1.61x10^-01^ | 115,723,466 |
| rs10513223 | 0.61 | 61 | 1.56 | 1.19x10^-01^ | 115,736,821 |
| rs2418260 | 0.47 | 71 | 2.03 | 4.20x10^-02^ | 115,750,707 |
| rs1571578 | 0.08 | 26 | 0.60 | 5.46x10^-01^ | 115,754,911 |
| rs1544102 | 0.45 | 65 | 0.63 | 5.31x10^-01^ | 115,763,309 |
| rs814697 | 0.59 | 71 | 0.61 | 5.45x10^-01^ | 115,766,587 |
| rs10759667 | 0.87 | 44 | 1.03 | 3.03x10^-01^ | 115,768,266 |
| rs814698 | 0.25 | 53 | 1.52 | 1.30x10^-01^ | 115,769,664 |
| rs1544065 | 0.16 | 35 | 0.33 | 7.44x10^-01^ | 115,773,004 |
| rs7021972 | 0.39 | 72 | 0.52 | 6.00x10^-01^ | 115,774,822 |
| rs10982023 | 0.08 | 24 | 0.00 | 1.00x10+00 | 115,776,612 |
| rs9332459 | 0.64 | 62 | 2.36 | 1.84x10^-02^ | 115,780,313 |
| rs814701 | 0.66 | 63 | 2.87 | 4.10x10^-03^ | 115,786,937 |
| rs1886129 | 0.68 | 70 | 1.33 | 1.84x10^-01^ | 115,790,826 |
| rs1330171 | 0.76 | 63 | 1.85 | 6.39x10^-02^ | 115,791,045 |
| rs4979316 | 0.33 | 61 | 0.06 | 9.56x10^-01^ | 115,802,355 |
| rs4979321 | 0.63 | 63 | 0.28 | 7.81x10^-01^ | 115,809,336 |
| rs1853546 | 0.68 | 62 | 0.54 | 5.88x10^-01^ | 115,818,465 |
| rs7023951 | 0.74 | 56 | 1.71 | 8.79x10^-02^ | 115,820,813 |
| rs4979327 | 0.71 | 67 | 2.25 | 2.42x10^-02^ | 115,821,137 |
| rs7041397 | 0.45 | 70 | 2.75 | 5.92x10^-03^ | 115,824,795 |
| rs752035 | 0.34 | 69 | 2.40 | 1.65x10^-02^ | 115,833,899 |
| rs1965753 | 0.61 | 67 | 1.39 | 1.65x10^-01^ | 115,839,747 |
| rs2078305 | 0.71 | 66 | 2.31 | 2.08x10^-02^ | 115,839,913 |
| rs7869264 | 0.10 | 35 | 1.68 | 9.26x10^-02^ | 115,842,360 |
| rs2418264 | 0.35 | 70 | 2.87 | 4.17x10^-03^ | 115,847,669 |
| rs12683020 | 0.82 | 46 | 0.60 | 5.51x10^-01^ | 115,854,482 |
| rs3810928 | 0.42 | 66 | 0.30 | 7.66x10^-01^ | 115,855,598 |
| rs2269454 | 0.74 | 54 | 1.74 | 8.22x10^-02^ | 115,862,471 |
| rs10759680 | 0.77 | 49 | 2.05 | 4.00x10^-02^ | 115,865,566 |
| rs12377342 | 0.27 | 54 | 0.86 | 3.90x10^-01^ | 115,870,140 |
| rs2251680 | 0.50 | 53 | 0.14 | 8.91x10^-01^ | 115,880,025 |
| rs2116403 | 0.65 | 55 | 0.75 | 4.54x10^-01^ | 115,882,620 |
| rs891725 | 0.87 | 43 | 2.18 | 2.94x10^-02^ | 115,887,022 |
| rs891724 | 0.81 | 46 | 1.74 | 8.27x10^-02^ | 115,887,186 |
| rs728070 | 0.84 | 45 | 0.03 | 9.74x10^-01^ | 115,889,383 |
| rs7036734 | 0.53 | 50 | 0.25 | 8.04x10^-01^ | 115,897,477 |
| rs3810929 | 0.33 | 59 | 0.37 | 7.09x10^-01^ | 115,899,590 |
| rs1560539 | 0.47 | 62 | 0.24 | 8.10x10^-01^ | 115,901,904 |
| rs7029198 | 0.63 | 58 | 0.76 | 4.48x10^-01^ | 115,906,875 |
| rs2163576 | 0.70 | 64 | 0.49 | 6.23x10^-01^ | 115,911,582 |
| rs1570589 | 0.77 | 54 | 0.71 | 4.76x10^-01^ | 115,914,176 |
| rs4978567 | 0.58 | 69 | 0.16 | 8.70x10^-01^ | 115,919,826 |
| rs7856967 | 0.03 | 9 | *** | *** | 115,927,226 |
| rs2217541 | 0.42 | 63 | 1.49 | 1.37x10^-01^ | 115,927,888 |
| rs10817572 | 0.77 | 54 | 0.42 | 6.74x10^-01^ | 115,931,522 |
| rs891721 | 0.66 | 53 | 0.50 | 6.17x10^-01^ | 115,936,787 |
| rs891720 | 0.26 | 54 | 1.09 | 2.74x10^-01^ | 115,937,034 |
| rs891719 | 0.04 | 10 | 0.00 | 1.00x10+00 | 115,937,270 |
| rs2761687 | 0.73 | 55 | 0.87 | 3.85x10^-01^ | 115,939,079 |
| rs2808769 | 0.84 | 46 | 0.29 | 7.74x10^-01^ | 115,939,880 |
| rs737174 | 0.27 | 58 | 0.16 | 8.76x10^-01^ | 115,943,231 |
| rs2043195 | 0.65 | 65 | 0.73 | 4.68x10^-01^ | 115,944,355 |
| rs919641 | 0.82 | 53 | 1.08 | 2.79x10^-01^ | 115,953,538 |
| rs2808772 | 0.56 | 68 | 0.10 | 9.23x10^-01^ | 115,953,615 |
| rs1469599 | 0.92 | 30 | 1.56 | 1.19x10^-01^ | 115,956,725 |
| rs2567725 | 0.56 | 71 | 0.61 | 5.41x10^-01^ | 115,961,521 |
| rs1431821 | 0.59 | 64 | 0.64 | 5.24x10^-01^ | 115,964,602 |
| rs737142 | 0.75 | 53 | 1.50 | 1.34x10^-01^ | 115,969,148 |
| rs891722 | 0.72 | 55 | 1.40 | 1.60x10^-01^ | 115,969,297 |
| rs1560538 | 0.70 | 56 | 1.34 | 1.80x10^-01^ | 115,972,174 |
| rs2808779 | 0.72 | 53 | 0.32 | 7.52x10^-01^ | 115,974,322 |
| rs2060132 | 0.67 | 62 | 0.16 | 8.72x10^-01^ | 115,978,732 |
| rs1431818 | 0.15 | 41 | 0.97 | 3.30x10^-01^ | 115,983,183 |
| rs891718 | 0.82 | 46 | 1.97 | 4.86x10^-02^ | 115,989,270 |
| rs4360357 | 0.21 | 57 | 1.48 | 1.38x10^-01^ | 115,994,263 |
| rs4979352 | 0.08 | 31 | 2.55 | 1.07x10^-02^ | 115,997,779 |
| rs2808789 | 0.17 | 50 | 0.41 | 6.80x10^-01^ | 115,999,533 |
| rs2567714 | 0.74 | 56 | 0.09 | 9.27x10^-01^ | 116,003,882 |
| rs2043197 | 0.25 | 58 | 0.38 | 7.05x10^-01^ | 116,006,701 |
| rs2060133 | 0.84 | 46 | 1.08 | 2.79x10^-01^ | 116,011,443 |
| rs7867146 | 0.25 | 59 | 0.53 | 5.99x10^-01^ | 116,015,130 |
| rs745490 | 0.92 | 26 | 0.04 | 9.70x10^-01^ | 116,017,070 |
| rs7870474 | 0.86 | 29 | 1.57 | 1.16x10^-01^ | 116,018,993 |
| rs10982110 | 0.31 | 56 | 0.20 | 8.38x10^-01^ | 116,019,873 |
| rs7019849 | 0.23 | 55 | 0.89 | 3.75x10^-01^ | 116,023,891 |
| rs7873541 | 0.33 | 61 | 0.63 | 5.29x10^-01^ | 116,026,289 |
| rs4294255 | 0.31 | 58 | 0.90 | 3.69x10^-01^ | 116,026,651 |
| rs871878 | 0.22 | 55 | 0.55 | 5.82x10^-01^ | 116,029,196 |
| rs1542737 | 0.24 | 49 | 0.18 | 8.58x10^-01^ | 116,036,691 |
| rs7850171 | 0.44 | 65 | 0.30 | 7.66x10^-01^ | 116,045,445 |
| rs902497 | 0.32 | 69 | 1.83 | 6.78x10^-02^ | 116,051,975 |
| rs2808800 | 0.35 | 69 | 1.64 | 1.02x10^-01^ | 116,053,140 |
| rs7028232 | 0.28 | 65 | 1.34 | 1.80x10^-01^ | 116,057,614 |
| rs923601 | 0.60 | 73 | 0.70 | 4.85x10^-01^ | 116,059,622 |
| rs923602 | 0.76 | 59 | 0.45 | 6.52x10^-01^ | 116,062,430 |
| rs2808768 | 0.53 | 67 | 0.92 | 3.59x10^-01^ | 116,063,963 |
| rs876440 | 0.72 | 62 | 0.21 | 8.32x10^-01^ | 116,068,327 |
| rs2636859 | 0.58 | 71 | 0.78 | 4.35x10^-01^ | 116,069,243 |
| rs5003737 | 0.52 | 68 | 0.48 | 6.29x10^-01^ | 116,072,010 |
| rs1490740 | 0.17 | 33 | 0.48 | 6.34x10^-01^ | 116,072,082 |
| rs1468008 | 0.41 | 64 | 0.61 | 5.41x10^-01^ | 116,073,651 |
| rs1490742 | 0.59 | 64 | 0.21 | 8.36x10^-01^ | 116,074,570 |
| rs1249721 | 0.64 | 72 | 0.08 | 9.38x10^-01^ | 116,076,581 |
| rs1766059 | 0.32 | 46 | 0.72 | 4.73x10^-01^ | 116,078,932 |
| rs1249744 | 0.30 | 62 | 0.42 | 6.77x10^-01^ | 116,083,173 |
| rs2636879 | 0.40 | 70 | 0.51 | 6.07x10^-01^ | 116,084,359 |
| rs1687410 | 0.82 | 49 | 0.06 | 9.53x10^-01^ | 116,090,841 |
| rs1249738 | 0.68 | 58 | 1.12 | 2.61x10^-01^ | 116,093,036 |
| rs1626295 | 0.51 | 67 | 1.72 | 8.60x10^-02^ | 116,102,498 |
| rs1000744 | 0.38 | 69 | 0.39 | 6.99x10^-01^ | 116,112,477 |
| rs7851482 | 0.41 | 55 | 0.10 | 9.23x10^-01^ | 116,118,107 |
| rs2787333 | 0.73 | 48 | 0.15 | 8.77x10^-01^ | 116,118,458 |
| rs2787334 | 0.56 | 55 | 0.22 | 8.22x10^-01^ | 116,118,659 |
| rs2636881 | 0.73 | 51 | 1.15 | 2.49x10^-01^ | 116,120,209 |
| rs1044531 | 0.07 | 30 | 0.91 | 3.65x10^-01^ | 116,138,471 |
| rs1687392 | 0.42 | 67 | 1.38 | 1.68x10^-01^ | 116,139,777 |
| rs1490744 | 0.53 | 67 | 0.52 | 6.02x10^-01^ | 116,151,413 |
| rs1249725 | 0.58 | 63 | 0.55 | 5.84x10^-01^ | 116,152,635 |
| rs1249726 | 0.55 | 65 | 0.71 | 4.81x10^-01^ | 116,153,607 |
| rs7866925 | 0.27 | 60 | 0.64 | 5.22x10^-01^ | 116,160,501 |
| rs2787348 | 0.20 | 51 | 0.47 | 6.37x10^-01^ | 116,161,661 |
| rs10513243 | 0.02 | 9 | *** | *** | 116,186,256 |
| rs2485744 | 0.86 | 45 | 2.44 | 1.45x10^-02^ | 116,195,753 |
| rs10817600 | 0.50 | 70 | 1.05 | 2.96x10^-01^ | 116,198,318 |
| rs10759693 | 0.54 | 72 | 1.04 | 2.99x10^-01^ | 116,202,074 |
| rs2274160 | 0.78 | 50 | 0.09 | 9.29x10^-01^ | 116,210,131 |
| rs4979387 | 0.78 | 48 | 0.12 | 9.05x10^-01^ | 116,226,498 |
| rs718841 | 0.27 | 62 | 0.81 | 4.16x10^-01^ | 116,237,856 |
| rs10982218 | 0.96 | 12 | 0.06 | 9.49x10^-01^ | 116,239,961 |
| rs4979392 | 0.47 | 64 | 0.61 | 5.43x10^-01^ | 116,244,184 |
| rs1324633 | 0.26 | 56 | 0.49 | 6.27x10^-01^ | 116,251,908 |
| rs942520 | 0.48 | 66 | 0.17 | 8.64x10^-01^ | 116,254,730 |
| rs4979397 | 0.78 | 46 | 0.43 | 6.67x10^-01^ | 116,259,709 |
| rs1324630 | 0.80 | 43 | 0.37 | 7.13x10^-01^ | 116,263,183 |
| rs4979407 | 0.14 | 38 | 0.00 | 1.00x10+00 | 116,267,936 |
| rs1000709 | 0.50 | 61 | 0.44 | 6.62x10^-01^ | 116,277,575 |
| rs725340 | 0.94 | 20 | 0.43 | 6.68x10^-01^ | 116,289,695 |
| rs1535960 | 0.54 | 65 | 0.71 | 4.81x10^-01^ | 116,291,173 |
| rs4979415 | 0.94 | 14 | 0.49 | 6.22x10^-01^ | 116,301,420 |
| rs4979418 | 0.48 | 61 | 0.69 | 4.92x10^-01^ | 116,303,904 |
| rs2296262 | 0.62 | 65 | 0.75 | 4.53x10^-01^ | 116,305,227 |
| rs1535963 | 0.90 | 27 | 0.54 | 5.90x10^-01^ | 116,312,038 |
| rs1408525 | 0.53 | 61 | 1.49 | 1.35x10^-01^ | 116,314,815 |
| rs10513248 | 0.62 | 71 | 1.28 | 2.00x10^-01^ | 116,318,665 |
| rs717138 | 0.06 | 17 | 0.34 | 7.32x10^-01^ | 116,319,151 |
| rs10817634 | 0.12 | 34 | 0.43 | 6.70x10^-01^ | 116,326,224 |
| rs4978596 | 0.95 | 16 | 0.30 | 7.63x10^-01^ | 116,334,438 |
| rs10513249 | 0.83 | 48 | 1.50 | 1.34x10^-01^ | 116,343,732 |
| rs10982289 | 0.81 | 44 | 0.52 | 6.07x10^-01^ | 116,354,595 |
| rs10114562 | 0.45 | 68 | 0.98 | 3.28x10^-01^ | 116,355,701 |
| rs730705 | 0.26 | 58 | 0.08 | 9.37x10^-01^ | 116,360,268 |
| rs4979427 | 0.30 | 60 | 0.45 | 6.51x10^-01^ | 116,363,247 |
| rs10817640 | 0.36 | 63 | 0.10 | 9.21x10^-01^ | 116,365,904 |
| rs12238437 | 0.91 | 33 | 0.98 | 3.25x10^-01^ | 116,388,287 |
| rs10982317 | 0.81 | 55 | 1.07 | 2.86x10^-01^ | 116,389,474 |
| rs10124511 | 0.71 | 58 | 0.33 | 7.39x10^-01^ | 116,393,285 |
| rs2274595 | 0.07 | 18 | 0.48 | 6.31x10^-01^ | 116,394,764 |
| rs7868842 | 0.34 | 61 | 0.16 | 8.73x10^-01^ | 116,400,550 |
| rs13297637 | 0.14 | 42 | 0.37 | 7.11x10^-01^ | 116,406,322 |
| rs10817646 | 0.25 | 54 | 0.36 | 7.21x10^-01^ | 116,406,882 |
| rs10115585 | 0.82 | 45 | 0.84 | 4.00x10^-01^ | 116,410,017 |
| rs4979444 | 0.47 | 67 | 0.90 | 3.68x10^-01^ | 116,415,730 |
| rs2418309 | 0.63 | 67 | 0.38 | 7.03x10^-01^ | 116,417,664 |
| rs7027369 | 0.26 | 58 | 0.87 | 3.87x10^-01^ | 116,423,776 |
| rs7036626 | 0.61 | 63 | 0.62 | 5.36x10^-01^ | 116,425,813 |
| rs1536737 | 0.13 | 40 | 0.99 | 3.23x10^-01^ | 116,429,299 |
| rs4246902 | 0.29 | 54 | 0.13 | 8.96x10^-01^ | 116,433,726 |
| rs2900590 | 0.23 | 54 | 0.03 | 9.78x10^-01^ | 116,441,140 |
| rs10759729 | 0.81 | 46 | 0.91 | 3.61x10^-01^ | 116,448,816 |
| rs1887784 | 0.75 | 48 | 0.59 | 5.53x10^-01^ | 116,461,032 |
| rs7043739 | 0.70 | 53 | 0.16 | 8.69x10^-01^ | 116,462,896 |
| rs2282003 | 0.57 | 57 | 0.31 | 7.57x10^-01^ | 116,469,048 |
| rs1359992 | 0.34 | 62 | 0.62 | 5.36x10^-01^ | 116,470,432 |
| rs2296948 | 0.38 | 64 | 0.73 | 4.65x10^-01^ | 116,473,506 |
| rs7869653 | 0.62 | 48 | 0.56 | 5.76x10^-01^ | 116,475,127 |
| rs9695517 | 0.42 | 59 | 0.42 | 6.76x10^-01^ | 116,479,676 |
| rs1075372 | 0.39 | 61 | 0.15 | 8.79x10^-01^ | 116,484,211 |
| rs12685734 | 0.75 | 58 | 0.36 | 7.19x10^-01^ | 116,486,963 |
| rs4979454 | 0.60 | 51 | 0.95 | 3.41x10^-01^ | 116,495,996 |
| rs2183019 | 0.34 | 57 | 0.94 | 3.47x10^-01^ | 116,513,448 |
| rs4979459 | 0.53 | 71 | 4.06 | 4.90x10^-05^ | 116,521,487 |
| rs7026324 | 0.37 | 65 | 2.60 | 9.30x10^-03^ | 116,523,764 |
| rs10817670 | 0.74 | 63 | 3.07 | 2.13x10^-03^ | 116,526,975 |
| rs11790825 | 0.18 | 44 | 3.29 | 1.02x10^-03^ | 116,531,680 |
| rs10982385 | 0.54 | 69 | 0.55 | 5.84x10^-01^ | 116,532,838 |
| rs2185935 | 0.27 | 66 | 1.48 | 1.38x10^-01^ | 116,542,063 |
| rs2068955 | 0.48 | 63 | 3.65 | 2.66x10^-04^ | 116,544,619 |
| rs10759736 | 0.85 | 46 | 1.18 | 2.37x10^-01^ | 116,563,013 |
| rs10513258 | 0.73 | 56 | 0.77 | 4.44x10^-01^ | 116,569,954 |
| rs4978609 | 0.90 | 37 | 3.65 | 2.64x10^-04^ | 116,570,140 |
| rs7048659 | 0.55 | 63 | 1.64 | 1.02x10^-01^ | 116,573,110 |
| rs7856856 | 0.72 | 62 | 0.38 | 7.03x10^-01^ | 116,580,731 |
| rs6478106 | 0.71 | 59 | 0.53 | 5.94x10^-01^ | 116,585,487 |
| rs10114470 | 0.67 | 61 | 0.20 | 8.43x10^-01^ | 116,587,593 |
| rs7847158 | 0.72 | 62 | 1.29 | 1.98x10^-01^ | 116,600,086 |
| rs4263839 | 0.33 | 59 | 0.14 | 8.91x10^-01^ | 116,606,261 |
| rs7862325 | 0.50 | 66 | 0.03 | 9.79x10^-01^ | 116,606,958 |
| rs1407306 | 0.84 | 38 | 0.43 | 6.70x10^-01^ | 116,626,230 |
| rs12337233 | 0.84 | 38 | 0.21 | 8.31x10^-01^ | 116,627,234 |
| rs4262377 | 0.14 | 39 | 0.00 | 1.00x10+00 | 116,629,395 |
| rs2006996 | 0.97 | 10 | 1.41 | 1.57x10^-01^ | 116,632,459 |
| rs722126 | 0.31 | 56 | 1.03 | 3.04x10^-01^ | 116,632,599 |
| rs7866342 | 0.32 | 61 | 0.92 | 3.55x10^-01^ | 116,667,390 |
| rs4979467 | 0.47 | 61 | 0.62 | 5.36x10^-01^ | 116,669,864 |
| rs7874896 | 0.70 | 63 | 1.83 | 6.73x10^-02^ | 116,676,700 |
| rs7863183 | 0.44 | 64 | 0.58 | 5.64x10^-01^ | 116,682,239 |
| rs7028891 | 0.49 | 66 | 0.45 | 6.54x10^-01^ | 116,684,836 |
| rs911605 | 0.34 | 63 | 0.52 | 6.03x10^-01^ | 116,694,811 |
| rs1322055 | 0.78 | 49 | 0.87 | 3.84x10^-01^ | 116,709,406 |
| rs6478118 | 0.96 | 11 | 0.64 | 5.25x10^-01^ | 116,712,967 |
| rs3789879 | 0.57 | 65 | 2.57 | 1.03x10^-02^ | 116,718,057 |
| rs1322058 | 0.61 | 66 | 2.37 | 1.80x10^-02^ | 116,724,368 |
| rs3181354 | 0.73 | 58 | 1.74 | 8.26x10^-02^ | 116,732,994 |
| rs3181346 | 0.03 | 9 | *** | *** | 116,734,343 |
| rs726658 | 0.73 | 58 | 2.10 | 3.57x10^-02^ | 116,736,087 |
| rs726657 | 0.60 | 66 | 2.21 | 2.73x10^-02^ | 116,736,157 |
| rs726656 | 0.64 | 62 | 2.18 | 2.96x10^-02^ | 116,736,269 |
| rs10759743 | 0.82 | 42 | 1.87 | 6.21x10^-02^ | 116,741,573 |
| rs979584 | 0.23 | 50 | 1.16 | 2.45x10^-01^ | 116,759,067 |
| rs1265917 | 0.47 | 64 | 1.68 | 9.35x10^-02^ | 116,762,015 |
| rs1265918 | 0.93 | 26 | 1.44 | 1.49x10^-01^ | 116,762,511 |
| rs10121268 | 0.37 | 71 | 2.21 | 2.69x10^-02^ | 116,766,135 |
| rs10123153 | 0.51 | 72 | 2.15 | 3.12x10^-02^ | 116,768,716 |
| rs2094793 | 0.13 | 39 | 1.47 | 1.41x10^-01^ | 116,775,145 |
| rs10982476 | 0.23 | 58 | 1.34 | 1.82x10^-01^ | 116,781,125 |
| rs1330366 | 0.25 | 57 | 2.53 | 1.13x10^-02^ | 116,809,350 |
| rs10817700 | 0.62 | 55 | 1.14 | 2.53x10^-01^ | 116,815,624 |
| rs7035322 | 0.74 | 53 | 0.96 | 3.36x10^-01^ | 116,826,994 |
| rs13321 | 0.30 | 56 | 0.49 | 6.28x10^-01^ | 116,832,404 |
| rs3789875 | 0.72 | 55 | 0.67 | 5.06x10^-01^ | 116,835,109 |
| rs1330361 | 0.86 | 43 | 0.57 | 5.70x10^-01^ | 116,837,215 |
| rs2274751 | 0.86 | 32 | 0.70 | 4.87x10^-01^ | 116,840,238 |
| rs2104772 | 0.49 | 66 | 1.50 | 1.33x10^-01^ | 116,848,606 |
| rs1547692 | 0.63 | 56 | 1.38 | 1.67x10^-01^ | 116,852,166 |
| rs1330365 | 0.24 | 60 | 1.45 | 1.47x10^-01^ | 116,857,504 |
| rs1537011 | 0.87 | 43 | 0.69 | 4.93x10^-01^ | 116,860,370 |
| rs1330368 | 0.47 | 68 | 1.37 | 1.70x10^-01^ | 116,860,847 |
| rs2236409 | 0.07 | 26 | 0.00 | 1.00x10+00 | 116,861,567 |
| rs7847271 | 0.89 | 30 | 0.19 | 8.53x10^-01^ | 116,870,633 |
| rs3789870 | 0.75 | 51 | 1.05 | 2.94x10^-01^ | 116,875,097 |
| rs1330351 | 0.51 | 65 | 0.18 | 8.56x10^-01^ | 116,880,743 |
| rs1757095 | 0.09 | 25 | 0.32 | 7.52x10^-01^ | 116,888,215 |
| rs2482077 | 0.45 | 68 | 0.06 | 9.50x10^-01^ | 116,891,493 |
| rs1271449 | 0.31 | 59 | 0.31 | 7.58x10^-01^ | 116,892,343 |
| rs2026154 | 0.85 | 44 | 0.46 | 6.47x10^-01^ | 116,901,361 |
| rs1250026 | 0.43 | 64 | 0.03 | 9.79x10^-01^ | 116,902,327 |
| rs2480932 | 0.58 | 64 | 0.10 | 9.20x10^-01^ | 116,904,498 |
| rs3748166 | 0.31 | 60 | 2.06 | 3.91x10^-02^ | 116,919,970 |
| rs945257 | 0.67 | 57 | 2.12 | 3.44x10^-02^ | 116,925,676 |
| rs945256 | 0.68 | 61 | 2.06 | 3.98x10^-02^ | 116,925,845 |
| rs1360290 | 0.25 | 65 | 1.83 | 6.77x10^-02^ | 116,934,360 |
| rs1537722 | 0.67 | 59 | 0.16 | 8.72x10^-01^ | 116,936,170 |
| rs1537720 | 0.64 | 61 | 1.42 | 1.54x10^-01^ | 116,937,364 |
| rs2012775 | 0.31 | 65 | 1.47 | 1.43x10^-01^ | 116,943,669 |
| rs1335334 | 0.87 | 38 | 0.46 | 6.46x10^-01^ | 116,949,614 |
| rs10982585 | 0.88 | 37 | 0.47 | 6.38x10^-01^ | 116,963,649 |
| rs1537725 | 0.06 | 22 | 0.14 | 8.93x10^-01^ | 116,971,519 |
| rs4979508 | 0.22 | 47 | 0.12 | 9.04x10^-01^ | 116,972,637 |
| rs9886855 | 0.87 | 37 | 1.34 | 1.80x10^-01^ | 116,987,865 |
| rs1414145 | 0.34 | 63 | 1.56 | 1.19x10^-01^ | 116,994,910 |
| rs1110169 | 0.72 | 58 | 1.69 | 9.08x10^-02^ | 116,999,569 |
| rs1414148 | 0.76 | 45 | 0.06 | 9.51x10^-01^ | 117,003,433 |
| rs1889651 | 0.42 | 62 | 2.21 | 2.71x10^-02^ | 117,010,389 |
| rs1335359 | 0.84 | 46 | 0.61 | 5.42x10^-01^ | 117,013,859 |
| rs1335360 | 0.76 | 46 | 0.35 | 7.30x10^-01^ | 117,019,574 |
| rs10513266 | 0.70 | 62 | 0.67 | 5.03x10^-01^ | 117,024,589 |
| rs10513267 | 0.70 | 58 | 0.15 | 8.82x10^-01^ | 117,028,187 |
| rs955387 | 0.13 | 35 | 0.28 | 7.78x10^-01^ | 117,031,013 |
| rs2992140 | 0.84 | 46 | 2.03 | 4.22x10^-02^ | 117,034,386 |
| rs2989505 | 0.14 | 41 | 1.58 | 1.15x10^-01^ | 117,036,045 |
| rs2989508 | 0.33 | 62 | 0.12 | 9.02x10^-01^ | 117,040,772 |
| rs883402 | 0.87 | 39 | 1.84 | 6.51x10^-02^ | 117,053,110 |
| rs4526430 | 0.59 | 58 | 0.72 | 4.69x10^-01^ | 117,054,853 |
| rs1414153 | 0.20 | 51 | 1.42 | 1.55x10^-01^ | 117,082,174 |
| rs1335338 | 0.62 | 65 | 0.09 | 9.25x10^-01^ | 117,091,803 |
| rs2418327 | 0.88 | 42 | 0.10 | 9.21x10^-01^ | 117,092,162 |
| rs1029341 | 0.10 | 33 | 0.71 | 4.80x10^-01^ | 117,095,676 |
| rs1002272 | 0.53 | 66 | 0.30 | 7.67x10^-01^ | 117,100,824 |
| rs7024659 | 0.63 | 62 | 0.83 | 4.06x10^-01^ | 117,102,514 |
| rs1157836 | 0.43 | 65 | 1.56 | 1.19x10^-01^ | 117,108,543 |
| rs13296996 | 0.15 | 34 | 0.25 | 8.03x10^-01^ | 117,117,827 |
| rs7047151 | 0.35 | 53 | 0.47 | 6.40x10^-01^ | 117,128,773 |
| rs2285317 | 0.34 | 53 | 0.60 | 5.48x10^-01^ | 117,133,313 |
| rs10817748 | 0.17 | 43 | 1.71 | 8.72x10^-02^ | 117,138,159 |
| rs1981499 | 0.34 | 59 | 0.20 | 8.40x10^-01^ | 117,147,045 |
| rs13300054 | 0.52 | 62 | 0.10 | 9.22x10^-01^ | 117,153,448 |
| rs1029346 | 0.41 | 56 | 0.52 | 6.03x10^-01^ | 117,161,245 |
| rs974691 | 0.39 | 64 | 1.95 | 5.18x10^-02^ | 117,165,183 |
| rs972449 | 0.22 | 57 | 1.28 | 2.02x10^-01^ | 117,166,086 |
| rs4344149 | 0.11 | 33 | 0.21 | 8.38x10^-01^ | 117,166,618 |
| rs7859920 | 0.49 | 59 | 1.45 | 1.47x10^-01^ | 117,188,511 |
| rs10124685 | 0.88 | 32 | 0.55 | 5.84x10^-01^ | 117,192,372 |
| rs3893062 | 0.32 | 61 | 1.01 | 3.14x10^-01^ | 117,195,933 |
| rs2269700 | 0.61 | 54 | 1.53 | 1.25x10^-01^ | 117,203,384 |
| rs7853504 | 0.22 | 59 | 0.76 | 4.46x10^-01^ | 117,212,185 |
| rs976734 | 0.88 | 28 | 0.45 | 6.55x10^-01^ | 117,213,688 |
| rs4979524 | 0.82 | 54 | 0.82 | 4.11x10^-01^ | 117,217,438 |
| rs959486 | 0.12 | 35 | 0.59 | 5.56x10^-01^ | 117,217,878 |
| rs2020280 | 0.34 | 59 | 0.99 | 3.23x10^-01^ | 117,241,508 |
| rs2157663 | 0.89 | 34 | 0.50 | 6.21x10^-01^ | 117,253,323 |
| rs1632835 | 0.47 | 61 | 0.65 | 5.15x10^-01^ | 117,257,649 |
| rs10759783 | 0.59 | 70 | 1.19 | 2.33x10^-01^ | 117,273,668 |
| rs9284057 | 0.74 | 59 | 1.22 | 2.24x10^-01^ | 117,281,768 |
| rs4978641 | 0.12 | 29 | 0.13 | 8.96x10^-01^ | 117,284,107 |
| rs4979542 | 0.61 | 70 | 1.04 | 2.97x10^-01^ | 117,285,785 |
| rs6478171 | 0.71 | 67 | 1.32 | 1.86x10^-01^ | 117,289,916 |
| rs1989767 | 0.51 | 66 | 2.02 | 4.38x10^-02^ | 117,299,399 |
| rs2188049 | 0.41 | 70 | 2.61 | 9.05x10^-03^ | 117,305,595 |
| rs9409167 | 0.77 | 50 | 0.03 | 9.77x10^-01^ | 117,305,933 |
| rs2213920 | 0.82 | 56 | 1.72 | 8.58x10^-02^ | 117,311,405 |
| rs10982776 | 0.81 | 63 | 2.18 | 2.90x10^-02^ | 117,319,731 |
| rs1989770 | 0.90 | 41 | 1.98 | 4.78x10^-02^ | 117,324,377 |
| rs2213921 | 0.74 | 58 | 1.52 | 1.29x10^-01^ | 117,334,807 |
| rs916557 | 0.91 | 32 | 1.01 | 3.15x10^-01^ | 117,335,755 |
| rs2188053 | 0.69 | 51 | 1.32 | 1.85x10^-01^ | 117,339,571 |
| rs4978643 | 0.63 | 60 | 1.31 | 1.89x10^-01^ | 117,343,285 |
| rs10759800 | 0.60 | 71 | 2.15 | 3.15x10^-02^ | 117,369,795 |
| rs2106052 | 0.58 | 60 | 1.41 | 1.59x10^-01^ | 117,379,811 |
| rs875069 | 0.84 | 49 | 2.19 | 2.89x10^-02^ | 117,396,947 |
| rs12115665 | 0.91 | 37 | 2.14 | 3.26x10^-02^ | 117,408,582 |
| rs7860973 | 0.54 | 73 | 2.08 | 3.71x10^-02^ | 117,415,851 |
| rs4593632 | 0.43 | 68 | 2.45 | 1.43x10^-02^ | 117,420,399 |
| rs4979570 | 0.60 | 69 | 1.52 | 1.28x10^-01^ | 117,432,029 |
| rs2418400 | 0.83 | 42 | 0.03 | 9.73x10^-01^ | 117,454,440 |
| rs7040831 | 0.53 | 65 | 0.56 | 5.74x10^-01^ | 117,466,836 |
| rs4979573 | 0.66 | 60 | 0.78 | 4.36x10^-01^ | 117,470,899 |
| rs10982866 | 0.71 | 61 | 0.87 | 3.87x10^-01^ | 117,476,374 |
| rs966155 | 0.13 | 44 | 0.55 | 5.83x10^-01^ | 117,485,448 |
| rs10817823 | 0.77 | 59 | 0.88 | 3.81x10^-01^ | 117,497,717 |
| rs7469580 | 0.63 | 62 | 0.60 | 5.49x10^-01^ | 117,524,515 |
| rs2188075 | 0.16 | 41 | 1.08 | 2.81x10^-01^ | 117,545,801 |
| rs7856085 | 0.25 | 47 | 0.90 | 3.67x10^-01^ | 117,552,118 |
| rs1125441 | 0.91 | 24 | 0.09 | 9.27x10^-01^ | 117,554,265 |
| rs10429616 | 0.55 | 60 | 0.02 | 9.83x10^-01^ | 117,558,554 |
| rs689506 | 0.89 | 33 | 0.16 | 8.71x10^-01^ | 117,566,269 |
| rs9409171 | 0.35 | 58 | 0.22 | 8.29x10^-01^ | 117,586,800 |
| rs7043919 | 0.88 | 36 | 0.30 | 7.68x10^-01^ | 117,590,627 |
| rs7865455 | 0.48 | 67 | 0.24 | 8.14x10^-01^ | 117,592,816 |
| rs2157673 | 0.33 | 59 | 0.31 | 7.59x10^-01^ | 117,598,930 |
| rs10982926 | 0.90 | 24 | 1.21 | 2.28x10^-01^ | 117,608,644 |
| rs4979583 | 0.61 | 67 | 1.97 | 4.84x10^-02^ | 117,611,249 |
| rs4978654 | 0.76 | 47 | 0.97 | 3.32x10^-01^ | 117,623,932 |
| rs6478207 | 0.69 | 59 | 1.86 | 6.26x10^-02^ | 117,632,612 |
| rs10817839 | 0.75 | 53 | 1.41 | 1.57x10^-01^ | 117,637,658 |
| rs766102 | 0.30 | 50 | 0.12 | 9.05x10^-01^ | 117,648,471 |
| rs739343 | 0.63 | 54 | 0.84 | 4.00x10^-01^ | 117,652,001 |
| rs995804 | 0.51 | 56 | 0.94 | 3.49x10^-01^ | 117,684,631 |
| rs7029447 | 0.93 | 20 | 1.62 | 1.06x10^-01^ | 117,685,824 |
| rs9409174 | 0.90 | 32 | 2.10 | 3.54x10^-02^ | 117,687,453 |
| rs10982987 | 0.65 | 54 | 0.28 | 7.76x10^-01^ | 117,693,781 |
| rs9409175 | 0.41 | 63 | 1.80 | 7.24x10^-02^ | 117,696,707 |
| rs2900615 | 0.89 | 29 | 0.83 | 4.08x10^-01^ | 117,698,878 |
| rs7043151 | 0.96 | 13 | 0.28 | 7.82x10^-01^ | 117,726,133 |
| rs6478214 | 0.13 | 27 | 0.23 | 8.16x10^-01^ | 117,733,470 |
| rs1450197 | 0.32 | 56 | 1.10 | 2.71x10^-01^ | 117,746,213 |
| rs9632895 | 0.04 | 16 | 1.28 | 2.01x10^-01^ | 117,758,018 |
| rs4979603 | 0.04 | 13 | 1.61 | 1.08x10^-01^ | 117,777,859 |
| rs987546 | 0.10 | 34 | 1.44 | 1.49x10^-01^ | 117,779,527 |
| rs12551620 | 0.45 | 60 | 0.80 | 4.21x10^-01^ | 117,781,035 |
| rs204203 | 0.29 | 57 | 0.73 | 4.68x10^-01^ | 117,784,936 |
| rs204190 | 0.13 | 32 | 0.19 | 8.47x10^-01^ | 117,804,627 |
| rs204178 | 0.33 | 56 | 0.43 | 6.70x10^-01^ | 117,825,541 |
| rs204169 | 0.65 | 60 | 0.08 | 9.39x10^-01^ | 117,830,138 |
| rs204168 | 0.30 | 58 | 0.82 | 4.13x10^-01^ | 117,830,757 |
| rs10739458 | 0.18 | 50 | 0.78 | 4.38x10^-01^ | 117,851,561 |
| rs2213941 | 0.87 | 38 | 0.07 | 9.45x10^-01^ | 117,885,225 |
| rs204505 | 0.73 | 60 | 0.43 | 6.67x10^-01^ | 117,927,770 |
| rs204514 | 0.75 | 64 | 0.12 | 9.02x10^-01^ | 117,938,539 |
| rs386088 | 0.32 | 55 | 0.63 | 5.30x10^-01^ | 117,956,014 |
| rs1411821 | 0.25 | 57 | 0.62 | 5.38x10^-01^ | 117,974,868 |
| rs7026018 | 0.43 | 66 | 2.03 | 4.28x10^-02^ | 117,982,467 |
| rs3789280 | 0.67 | 61 | 2.48 | 1.32x10^-02^ | 117,993,193 |
| rs374560 | 0.52 | 67 | 0.83 | 4.10x10^-01^ | 118,020,365 |
| rs390623 | 0.87 | 36 | 0.17 | 8.63x10^-01^ | 118,028,734 |
| rs10983088 | 0.24 | 54 | 1.32 | 1.86x10^-01^ | 118,051,212 |
| rs1331141 | 0.80 | 52 | 2.56 | 1.05x10^-02^ | 118,066,405 |
| rs7854428 | 0.39 | 65 | 3.13 | 1.75x10^-03^ | 118,070,597 |
| rs4837294 | 0.18 | 44 | 0.37 | 7.11x10^-01^ | 118,074,296 |
| rs4837525 | 0.63 | 67 | 2.89 | 3.91x10^-03^ | 118,078,379 |
| rs7874262 | 0.15 | 33 | 0.59 | 5.52x10^-01^ | 118,080,839 |
| rs10513272 | 0.22 | 41 | 1.42 | 1.57x10^-01^ | 118,103,868 |
| rs7024490 | 0.89 | 29 | 0.72 | 4.73x10^-01^ | 118,112,450 |
| rs1570518 | 0.71 | 55 | 1.07 | 2.86x10^-01^ | 118,118,897 |
| rs3761843 | 0.35 | 55 | 0.57 | 5.71x10^-01^ | 118,136,010 |
| rs2418444 | 0.17 | 37 | 0.60 | 5.47x10^-01^ | 118,145,488 |
| rs7020782 | 0.71 | 53 | 0.78 | 4.38x10^-01^ | 118,146,702 |
| rs10120448 | 0.26 | 47 | 0.39 | 6.94x10^-01^ | 118,150,261 |
| rs1323438 | 0.27 | 52 | 1.47 | 1.43x10^-01^ | 118,155,352 |
| rs7853634 | 0.16 | 43 | 0.16 | 8.72x10^-01^ | 118,161,874 |
| rs3789299 | 0.47 | 63 | 0.31 | 7.61x10^-01^ | 118,166,791 |
| rs10983127 | 0.84 | 38 | 0.49 | 6.26x10^-01^ | 118,185,021 |
| rs4837374 | 0.76 | 60 | 0.99 | 3.24x10^-01^ | 118,194,437 |
| rs978201 | 0.37 | 60 | 0.08 | 9.40x10^-01^ | 118,196,851 |
| rs1858015 | 0.35 | 52 | 0.69 | 4.93x10^-01^ | 118,207,041 |
| rs4837515 | 0.33 | 55 | 2.29 | 2.18x10^-02^ | 118,222,313 |
| rs3747830 | 0.24 | 51 | 1.48 | 1.38x10^-01^ | 118,225,808 |
| rs1858016 | 0.04 | 14 | 0.00 | 1.00x10+00 | 118,229,193 |
| rs1396042 | 0.92 | 27 | 0.94 | 3.48x10^-01^ | 118,238,145 |
| rs4837545 | 0.66 | 60 | 1.88 | 6.06x10^-02^ | 118,269,067 |
| rs10817896 | 0.74 | 56 | 1.05 | 2.95x10^-01^ | 118,272,476 |
| rs10983184 | 0.63 | 68 | 1.68 | 9.23x10^-02^ | 118,273,879 |
| rs10759843 | 0.21 | 53 | 0.33 | 7.43x10^-01^ | 118,281,176 |
| rs2416560 | 0.62 | 69 | 2.14 | 3.21x10^-02^ | 118,292,028 |
| rs767432 | 0.71 | 61 | 1.35 | 1.76x10^-01^ | 118,298,008 |
| rs1321922 | 0.79 | 57 | 0.58 | 5.63x10^-01^ | 118,302,670 |
| rs4836732 | 0.48 | 73 | 0.17 | 8.67x10^-01^ | 118,306,516 |
| rs10983208 | 0.86 | 41 | 1.83 | 6.79x10^-02^ | 118,318,055 |
| rs888398 | 0.25 | 56 | 0.94 | 3.49x10^-01^ | 118,344,177 |
| rs1035272 | 0.11 | 36 | 0.92 | 3.58x10^-01^ | 118,347,923 |
| rs4837620 | 0.16 | 49 | 0.20 | 8.40x10^-01^ | 118,360,766 |
| rs4837659 | 0.67 | 67 | 0.42 | 6.73x10^-01^ | 118,405,113 |
| rs2416564 | 0.59 | 73 | 0.22 | 8.29x10^-01^ | 118,410,500 |
| rs4837683 | 0.57 | 68 | 0.18 | 8.57x10^-01^ | 118,424,756 |
| rs1742822 | 0.08 | 30 | 0.44 | 6.59x10^-01^ | 118,459,582 |
| rs1742830 | 0.49 | 67 | 1.04 | 2.97x10^-01^ | 118,463,533 |
| rs2165555 | 0.93 | 26 | 2.11 | 3.48x10^-02^ | 118,465,636 |
| rs803938 | 0.10 | 36 | 0.04 | 9.68x10^-01^ | 118,493,890 |
| rs803932 | 0.34 | 70 | 1.22 | 2.24x10^-01^ | 118,497,841 |
| rs4141711 | 0.14 | 47 | 0.12 | 9.02x10^-01^ | 118,511,702 |
| rs719535 | 0.78 | 54 | 0.80 | 4.26x10^-01^ | 118,516,091 |
| rs2165554 | 0.64 | 69 | 1.61 | 1.08x10^-01^ | 118,519,595 |
| rs1441747 | 0.45 | 69 | 1.76 | 7.88x10^-02^ | 118,527,632 |
| rs2274414 | 0.08 | 22 | 0.00 | 1.00x10+00 | 118,535,396 |
| rs10983334 | 0.26 | 58 | 0.58 | 5.64x10^-01^ | 118,543,563 |
| rs2416570 | 0.42 | 62 | 0.20 | 8.42x10^-01^ | 118,548,616 |
| rs1372330 | 0.87 | 36 | 1.31 | 1.90x10^-01^ | 118,559,409 |
| rs10759854 | 0.35 | 59 | 0.61 | 5.41x10^-01^ | 118,562,266 |
| rs4836849 | 0.60 | 68 | 0.26 | 7.94x10^-01^ | 118,566,196 |
| rs1441751 | 0.96 | 11 | 1.19 | 2.35x10^-01^ | 118,576,480 |
| rs1822199 | 0.92 | 31 | 0.43 | 6.70x10^-01^ | 118,580,455 |
| rs1372338 | 0.48 | 62 | 1.53 | 1.26x10^-01^ | 118,580,682 |
| rs4837864 | 0.11 | 30 | 0.00 | 1.00x10+00 | 118,584,254 |
| rs7030327 | 0.56 | 59 | 0.15 | 8.82x10^-01^ | 118,585,842 |
| rs10817934 | 0.30 | 66 | 1.33 | 1.83x10^-01^ | 118,589,872 |
| rs8181035 | 0.75 | 58 | 0.95 | 3.41x10^-01^ | 118,600,905 |
| rs10983355 | 0.27 | 56 | 2.61 | 9.20x10^-03^ | 118,606,354 |
| rs7853590 | 0.77 | 53 | 0.05 | 9.59x10^-01^ | 118,607,529 |
| rs1155556 | 0.50 | 73 | 1.84 | 6.57x10^-02^ | 118,612,155 |
| rs1465918 | 0.88 | 32 | 1.99 | 4.63x10^-02^ | 118,612,697 |
| rs1326810 | 0.73 | 63 | 1.54 | 1.23x10^-01^ | 118,625,880 |
| rs943309 | 0.18 | 44 | 0.34 | 7.33x10^-01^ | 118,639,347 |
| rs1326813 | 0.69 | 57 | 1.30 | 1.95x10^-01^ | 118,648,835 |
| rs1536427 | 0.85 | 36 | 2.10 | 3.57x10^-02^ | 118,655,561 |
| rs1536428 | 0.67 | 46 | 1.85 | 6.46x10^-02^ | 118,655,863 |
| rs1359702 | 0.76 | 51 | 1.82 | 6.82x10^-02^ | 118,658,651 |
| rs10491574 | 0.88 | 33 | 0.25 | 7.99x10^-01^ | 118,662,259 |
| rs943306 | 0.61 | 61 | 1.55 | 1.22x10^-01^ | 118,664,496 |
| rs2026587 | 0.88 | 33 | 1.15 | 2.51x10^-01^ | 118,666,110 |
| rs4837955 | 0.47 | 65 | 1.79 | 7.38x10^-02^ | 118,666,303 |
| rs4837957 | 0.10 | 31 | 0.14 | 8.89x10^-01^ | 118,669,388 |
| rs4837961 | 0.12 | 36 | 0.52 | 6.04x10^-01^ | 118,672,438 |
| rs4837968 | 0.42 | 60 | 2.29 | 2.22x10^-02^ | 118,681,106 |
| rs10817952 | 0.56 | 60 | 3.03 | 2.45x10^-03^ | 118,681,878 |
| rs2418445 | 0.51 | 64 | 1.95 | 5.11x10^-02^ | 118,688,265 |
| rs3843826 | 0.11 | 27 | 1.36 | 1.74x10^-01^ | 118,699,510 |
| rs4838008 | 0.42 | 62 | 0.59 | 5.58x10^-01^ | 118,709,197 |
| rs944323 | 0.44 | 56 | 1.23 | 2.18x10^-01^ | 118,714,543 |
| rs7018707 | 0.27 | 61 | 1.56 | 1.18x10^-01^ | 118,716,804 |
| rs11788987 | 0.04 | 17 | 2.41 | 1.58x10^-02^ | 118,717,375 |
| rs4838020 | 0.87 | 27 | 1.17 | 2.41x10^-01^ | 118,725,053 |
| rs4838023 | 0.44 | 60 | 1.57 | 1.17x10^-01^ | 118,728,990 |
| rs2150197 | 0.49 | 63 | 0.72 | 4.75x10^-01^ | 118,742,439 |
| rs10116120 | 0.27 | 55 | 2.28 | 2.25x10^-02^ | 118,747,593 |
| rs12552544 | 0.23 | 54 | 1.49 | 1.35x10^-01^ | 118,750,009 |
| rs3849144 | 0.78 | 48 | 1.33 | 1.83x10^-01^ | 118,752,154 |
| rs7044421 | 0.86 | 39 | 1.12 | 2.64x10^-01^ | 118,757,920 |
| rs2150199 | 0.30 | 56 | 1.64 | 1.00x10^-01^ | 118,765,546 |
| rs10817970 | 0.43 | 60 | 0.41 | 6.86x10^-01^ | 118,780,289 |
| rs1928994 | 0.20 | 48 | 0.03 | 9.80x10^-01^ | 118,782,795 |
| rs7341922 | 0.26 | 51 | 2.11 | 3.51x10^-02^ | 118,788,380 |
| rs7848841 | 0.94 | 18 | 0.87 | 3.86x10^-01^ | 118,792,975 |
| rs4144636 | 0.77 | 47 | 1.34 | 1.81x10^-01^ | 118,802,528 |
| rs7042036 | 0.65 | 49 | 1.90 | 5.74x10^-02^ | 118,806,790 |
| rs3761845 | 0.56 | 68 | 0.27 | 7.84x10^-01^ | 118,810,301 |
| rs10739481 | 0.71 | 56 | 1.21 | 2.25x10^-01^ | 118,817,665 |
| rs1999597 | 0.75 | 51 | 0.94 | 3.48x10^-01^ | 118,822,896 |
| rs867850 | 0.28 | 56 | 0.11 | 9.12x10^-01^ | 118,823,807 |
| rs1334094 | 0.21 | 47 | 1.78 | 7.54x10^-02^ | 118,824,049 |
| rs11792541 | 0.47 | 57 | 0.44 | 6.61x10^-01^ | 118,829,328 |
| rs7857377 | 0.43 | 69 | 0.07 | 9.43x10^-01^ | 118,832,071 |
| rs1334079 | 0.74 | 47 | 1.33 | 1.85x10^-01^ | 118,837,389 |
| rs1931302 | 0.26 | 50 | 0.73 | 4.64x10^-01^ | 118,842,262 |
| rs10817982 | 0.32 | 58 | 0.75 | 4.51x10^-01^ | 118,847,134 |
| rs10513281 | 0.81 | 43 | 0.72 | 4.70x10^-01^ | 118,852,535 |
| rs7853166 | 0.49 | 66 | 0.08 | 9.37x10^-01^ | 118,860,825 |
| rs4087490 | 0.09 | 30 | 1.17 | 2.41x10^-01^ | 118,863,689 |
| rs7848256 | 0.84 | 36 | 1.72 | 8.63x10^-02^ | 118,866,423 |
| rs10983506 | 0.91 | 23 | 0.93 | 3.54x10^-01^ | 118,875,611 |
| rs10759896 | 0.09 | 29 | 0.16 | 8.76x10^-01^ | 118,880,165 |
| rs1889318 | 0.45 | 61 | 1.35 | 1.78x10^-01^ | 118,882,872 |
| rs1105892 | 0.55 | 56 | 1.46 | 1.45x10^-01^ | 118,883,034 |
| rs1577133 | 0.71 | 63 | 1.05 | 2.92x10^-01^ | 118,883,890 |
| rs1334086 | 0.49 | 59 | 0.72 | 4.70x10^-01^ | 118,890,634 |
| rs944985 | 0.87 | 35 | 0.38 | 7.04x10^-01^ | 118,890,900 |
| rs10983517 | 0.73 | 53 | 0.51 | 6.09x10^-01^ | 118,897,937 |
| rs944980 | 0.45 | 60 | 1.40 | 1.62x10^-01^ | 118,910,034 |
| rs10817996 | 0.90 | 29 | 1.22 | 2.22x10^-01^ | 118,913,629 |
| rs3923774 | 0.79 | 45 | 1.19 | 2.35x10^-01^ | 118,920,814 |
| rs1413413 | 0.82 | 41 | 1.33 | 1.84x10^-01^ | 118,938,881 |
| rs4090221 | 0.27 | 65 | 0.60 | 5.46x10^-01^ | 118,941,199 |
| rs1572153 | 0.53 | 66 | 0.51 | 6.12x10^-01^ | 118,959,350 |
| rs6478282 | 0.72 | 52 | 0.16 | 8.69x10^-01^ | 118,964,916 |
| rs1413409 | 0.32 | 64 | 0.08 | 9.33x10^-01^ | 118,979,210 |
| rs977937 | 0.45 | 71 | 0.34 | 7.31x10^-01^ | 118,982,470 |
| rs4838267 | 0.42 | 57 | 0.36 | 7.16x10^-01^ | 118,989,653 |
| rs958804 | 0.68 | 50 | 0.58 | 5.63x10^-01^ | 118,998,674 |
| rs915281 | 0.54 | 60 | 1.88 | 6.03x10^-02^ | 119,007,784 |
| rs2184543 | 0.39 | 62 | 1.91 | 5.57x10^-02^ | 119,026,808 |
| rs1023294 | 0.38 | 61 | 2.03 | 4.19x10^-02^ | 119,027,727 |
| rs1160245 | 0.50 | 63 | 1.25 | 2.13x10^-01^ | 119,032,741 |
| rs1160246 | 0.43 | 64 | 1.75 | 8.01x10^-02^ | 119,032,915 |
| rs3901974 | 0.29 | 52 | 1.55 | 1.21x10^-01^ | 119,048,518 |
| rs1335420 | 0.86 | 38 | 1.88 | 6.06x10^-02^ | 119,051,936 |
| rs3900788 | 0.29 | 60 | 1.51 | 1.30x10^-01^ | 119,068,769 |
| rs4837062 | 0.72 | 54 | 0.19 | 8.51x10^-01^ | 119,071,186 |
| rs6478300 | 0.76 | 51 | 0.99 | 3.22x10^-01^ | 119,075,590 |
| rs10513297 | 0.50 | 64 | 0.43 | 6.68x10^-01^ | 119,090,284 |
| rs7862566 | 0.14 | 36 | 0.16 | 8.74x10^-01^ | 119,093,842 |
| rs2416594 | 0.43 | 67 | 0.96 | 3.37x10^-01^ | 119,100,195 |
| rs1572307 | 0.20 | 50 | 0.00 | 1.00x10+00 | 119,111,770 |
| rs1335411 | 0.42 | 70 | 0.24 | 8.14x10^-01^ | 119,119,743 |
| rs7021801 | 0.50 | 69 | 0.17 | 8.63x10^-01^ | 119,125,525 |
| rs1339922 | 0.51 | 70 | 0.47 | 6.39x10^-01^ | 119,137,997 |
| rs10513299 | 0.06 | 24 | 1.31 | 1.92x10^-01^ | 119,144,798 |
| rs10983629 | 0.91 | 26 | 0.72 | 4.71x10^-01^ | 119,146,741 |
| rs4240415 | 0.13 | 37 | 0.02 | 9.81x10^-01^ | 119,149,330 |
| rs7862524 | 0.65 | 52 | 0.52 | 6.00x10^-01^ | 119,151,562 |
| rs10818029 | 0.52 | 59 | 0.43 | 6.66x10^-01^ | 119,154,049 |
| rs10818030 | 0.26 | 62 | 0.87 | 3.83x10^-01^ | 119,166,291 |
| rs10513300 | 0.94 | 19 | 0.49 | 6.22x10^-01^ | 119,170,027 |
| rs2900139 | 0.28 | 59 | 0.87 | 3.85x10^-01^ | 119,177,552 |
| rs1339930 | 0.34 | 58 | 0.78 | 4.35x10^-01^ | 119,181,567 |
| rs10739491 | 0.57 | 69 | 0.82 | 4.12x10^-01^ | 119,188,537 |
| rs4837163 | 0.80 | 47 | 0.37 | 7.10x10^-01^ | 119,194,146 |
| rs1416942 | 0.37 | 75 | 0.70 | 4.81x10^-01^ | 119,194,670 |
| rs10513301 | 0.23 | 56 | 0.05 | 9.57x10^-01^ | 119,201,598 |
| rs4240422 | 0.45 | 61 | 0.05 | 9.59x10^-01^ | 119,203,287 |
| rs7023773 | 0.61 | 55 | 1.46 | 1.45x10^-01^ | 119,219,407 |
| rs9299252 | 0.53 | 59 | 1.52 | 1.29x10^-01^ | 119,224,082 |
| rs955302 | 0.30 | 52 | 1.23 | 2.17x10^-01^ | 119,225,813 |
| rs1415377 | 0.87 | 35 | 0.81 | 4.18x10^-01^ | 119,231,794 |
| rs1337212 | 0.15 | 47 | 0.29 | 7.74x10^-01^ | 119,239,171 |
| rs1337213 | 0.66 | 57 | 0.61 | 5.41x10^-01^ | 119,243,964 |
| rs4836608 | 0.55 | 60 | 1.33 | 1.82x10^-01^ | 119,256,451 |
| rs1339923 | 0.42 | 69 | 2.08 | 3.77x10^-02^ | 119,257,509 |
| rs1415381 | 0.75 | 58 | 1.10 | 2.73x10^-01^ | 119,277,220 |
| rs1337206 | 0.29 | 66 | 1.54 | 1.23x10^-01^ | 119,288,572 |
| rs952567 | 0.85 | 51 | 0.47 | 6.36x10^-01^ | 119,291,119 |
| rs1337215 | 0.62 | 53 | 0.63 | 5.30x10^-01^ | 119,294,732 |
| rs1252012 | 0.50 | 61 | 0.95 | 3.44x10^-01^ | 119,300,860 |
| rs549493 | 0.08 | 27 | 0.00 | 1.00x10+00 | 119,316,917 |
| rs12342331 | 0.15 | 42 | 0.00 | 1.00x10+00 | 119,318,352 |
| rs10491855 | 0.56 | 61 | 0.44 | 6.62x10^-01^ | 119,335,541 |
| rs495062 | 0.77 | 51 | 0.26 | 7.92x10^-01^ | 119,339,629 |
| rs540178 | 0.74 | 55 | 0.55 | 5.84x10^-01^ | 119,349,544 |
| rs1329064 | 0.17 | 46 | 0.65 | 5.16x10^-01^ | 119,353,313 |
| rs571823 | 0.59 | 65 | 0.87 | 3.84x10^-01^ | 119,360,477 |
| rs558789 | 0.23 | 51 | 0.76 | 4.48x10^-01^ | 119,374,399 |
| rs541306 | 0.54 | 65 | 0.17 | 8.67x10^-01^ | 119,378,838 |
| rs498180 | 0.32 | 57 | 0.00 | 1.00x10+00 | 119,396,944 |
| rs1013985 | 0.44 | 61 | 0.15 | 8.80x10^-01^ | 119,414,729 |
| rs7038925 | 0.19 | 59 | 0.93 | 3.53x10^-01^ | 119,423,543 |
| rs12685267 | 0.15 | 46 | 0.59 | 5.55x10^-01^ | 119,424,637 |
| rs10818053 | 0.91 | 28 | 0.84 | 4.04x10^-01^ | 119,424,888 |
| rs1887904 | 0.67 | 61 | 0.50 | 6.16x10^-01^ | 119,430,103 |
| rs4570241 | 0.80 | 48 | 0.81 | 4.19x10^-01^ | 119,436,799 |
| rs7032255 | 0.35 | 55 | 1.08 | 2.81x10^-01^ | 119,439,908 |
| rs4837462 | 0.53 | 59 | 0.36 | 7.18x10^-01^ | 119,450,040 |
| rs1887905 | 0.50 | 56 | 0.53 | 5.99x10^-01^ | 119,456,941 |
| rs745507 | 0.93 | 20 | 0.72 | 4.74x10^-01^ | 119,458,580 |
| rs1410851 | 0.18 | 41 | 0.99 | 3.23x10^-01^ | 119,464,417 |
| rs10448253 | 0.92 | 23 | 2.27 | 2.33x10^-02^ | 119,469,292 |
| rs1329061 | 0.63 | 45 | 1.08 | 2.78x10^-01^ | 119,476,938 |
| rs1329057 | 0.79 | 42 | 0.49 | 6.24x10^-01^ | 119,481,767 |
| rs10818069 | 0.92 | 24 | 2.46 | 1.38x10^-02^ | 119,493,792 |
| rs10759930 | 0.36 | 68 | 1.34 | 1.80x10^-01^ | 119,501,442 |
| rs2737191 | 0.74 | 60 | 0.68 | 4.98x10^-01^ | 119,502,536 |
| rs11536889 | 0.14 | 43 | 1.44 | 1.51x10^-01^ | 119,517,952 |
| rs1927906 | 0.89 | 30 | 1.58 | 1.15x10^-01^ | 119,519,936 |
| rs1554973 | 0.73 | 54 | 0.57 | 5.67x10^-01^ | 119,520,633 |
| rs913930 | 0.66 | 61 | 1.22 | 2.22x10^-01^ | 119,523,830 |
| rs1927905 | 0.06 | 23 | 0.35 | 7.29x10^-01^ | 119,525,129 |
| rs7045953 | 0.17 | 50 | 0.45 | 6.56x10^-01^ | 119,525,616 |
| rs10759934 | 0.48 | 68 | 0.44 | 6.63x10^-01^ | 119,528,817 |
| rs10465133 | 0.56 | 68 | 0.33 | 7.44x10^-01^ | 119,537,779 |
| rs7866830 | 0.23 | 52 | 0.58 | 5.65x10^-01^ | 119,542,086 |
| rs943840 | 0.44 | 67 | 0.28 | 7.80x10^-01^ | 119,552,361 |
| rs4837502 | 0.24 | 49 | 0.21 | 8.36x10^-01^ | 119,557,665 |
| rs7027711 | 0.83 | 37 | 0.60 | 5.48x10^-01^ | 119,563,999 |
| rs7357627 | 0.57 | 66 | 1.41 | 1.59x10^-01^ | 119,575,166 |
| rs7046020 | 0.69 | 54 | 0.64 | 5.24x10^-01^ | 119,576,672 |
| rs2149361 | 0.82 | 44 | 0.29 | 7.71x10^-01^ | 119,600,380 |
| rs1927923 | 0.16 | 36 | 0.49 | 6.22x10^-01^ | 119,602,212 |
| rs2149358 | 0.79 | 45 | 0.70 | 4.85x10^-01^ | 119,611,129 |
| rs10759948 | 0.68 | 64 | 0.12 | 9.07x10^-01^ | 119,616,455 |
| rs7854254 | 0.46 | 68 | 0.24 | 8.14x10^-01^ | 119,624,938 |
| rs661066 | 0.10 | 30 | 0.78 | 4.39x10^-01^ | 119,645,597 |
| rs493709 | 0.09 | 25 | 1.13 | 2.57x10^-01^ | 119,648,239 |
| rs5007493 | 0.74 | 52 | 0.07 | 9.44x10^-01^ | 119,663,462 |
| rs10818097 | 0.70 | 55 | 0.06 | 9.53x10^-01^ | 119,666,420 |
| rs908198 | 0.24 | 53 | 0.78 | 4.38x10^-01^ | 119,676,622 |
| rs10818099 | 0.59 | 74 | 0.98 | 3.30x10^-01^ | 119,691,906 |
| rs623089 | 0.46 | 75 | 0.63 | 5.30x10^-01^ | 119,723,274 |
| rs1514775 | 0.21 | 38 | 1.13 | 2.60x10^-01^ | 119,734,912 |
| rs551589 | 0.66 | 59 | 1.21 | 2.25x10^-01^ | 119,738,298 |
| rs2809389 | 0.04 | 16 | 0.98 | 3.27x10^-01^ | 119,769,582 |
| rs2780241 | 0.32 | 53 | 0.40 | 6.91x10^-01^ | 119,778,480 |
| rs955117 | 0.57 | 65 | 1.42 | 1.57x10^-01^ | 119,778,886 |
| rs7853395 | 0.15 | 39 | 1.10 | 2.71x10^-01^ | 119,781,464 |
| rs1930704 | 0.22 | 44 | 1.02 | 3.09x10^-01^ | 119,786,519 |
| rs2722851 | 0.23 | 46 | 0.71 | 4.75x10^-01^ | 119,817,472 |
| rs2806073 | 0.18 | 38 | 1.33 | 1.84x10^-01^ | 119,818,748 |
| rs2809418 | 0.28 | 56 | 0.90 | 3.66x10^-01^ | 119,825,432 |
| rs2722841 | 0.51 | 63 | 1.23 | 2.17x10^-01^ | 119,825,857 |
| rs5009086 | 0.52 | 61 | 1.58 | 1.14x10^-01^ | 119,827,733 |
| rs2900142 | 0.56 | 59 | 1.31 | 1.91x10^-01^ | 119,871,703 |
| rs2252957 | 0.30 | 59 | 0.66 | 5.13x10^-01^ | 119,882,611 |
| rs2806106 | 0.14 | 37 | 0.07 | 9.42x10^-01^ | 119,893,921 |
| rs1327808 | 0.17 | 41 | 0.75 | 4.53x10^-01^ | 119,936,211 |
| rs955259 | 0.03 | 8 | *** | *** | 119,944,806 |
| rs12238247 | 0.74 | 55 | 0.06 | 9.56x10^-01^ | 119,964,488 |
| rs2998008 | 0.19 | 48 | 1.90 | 5.79x10^-02^ | 119,969,430 |
| rs2806063 | 0.52 | 62 | 0.02 | 9.81x10^-01^ | 119,988,085 |
| rs12552190 | 0.54 | 64 | 0.45 | 6.53x10^-01^ | 120,002,058 |
| rs1327797 | 0.26 | 59 | 0.24 | 8.13x10^-01^ | 120,007,406 |
| rs4836712 | 0.86 | 39 | 0.22 | 8.23x10^-01^ | 120,014,211 |
| rs10983917 | 0.40 | 58 | 0.58 | 5.62x10^-01^ | 120,015,407 |
| rs927953 | 0.30 | 46 | 0.40 | 6.89x10^-01^ | 120,028,015 |
| rs1410201 | 0.82 | 45 | 1.02 | 3.08x10^-01^ | 120,032,590 |
| rs957568 | 0.42 | 64 | 1.08 | 2.81x10^-01^ | 120,054,713 |
| rs10818131 | 0.55 | 62 | 0.08 | 9.40x10^-01^ | 120,068,146 |
| rs876347 | 0.27 | 53 | 0.35 | 7.28x10^-01^ | 120,070,850 |
| rs1927320 | 0.87 | 39 | 1.11 | 2.69x10^-01^ | 120,077,818 |
| rs10818132 | 0.15 | 44 | 1.01 | 3.13x10^-01^ | 120,097,449 |
| rs1327816 | 0.77 | 53 | 1.50 | 1.34x10^-01^ | 120,099,214 |
| rs1418504 | 0.04 | 9 | *** | *** | 120,108,297 |
| rs12346589 | 0.30 | 56 | 0.71 | 4.75x10^-01^ | 120,117,443 |
| rs4604517 | 0.90 | 30 | 0.64 | 5.22x10^-01^ | 120,124,270 |
| rs10513330 | 0.71 | 53 | 0.28 | 7.83x10^-01^ | 120,124,638 |
| rs7866799 | 0.58 | 61 | 0.56 | 5.79x10^-01^ | 120,134,794 |
| rs1342482 | 0.19 | 46 | 0.12 | 9.06x10^-01^ | 120,141,335 |
| rs12351957 | 0.78 | 53 | 0.54 | 5.92x10^-01^ | 120,151,914 |
| rs1418500 | 0.48 | 70 | 0.10 | 9.23x10^-01^ | 120,162,167 |
| rs2066387 | 0.64 | 61 | 0.08 | 9.36x10^-01^ | 120,178,850 |
| rs7855921 | 0.69 | 63 | 0.07 | 9.42x10^-01^ | 120,188,881 |
| rs1342501 | 0.90 | 34 | 0.10 | 9.23x10^-01^ | 120,196,645 |
| rs1418510 | 0.10 | 27 | 0.29 | 7.70x10^-01^ | 120,202,384 |
| rs4837559 | 0.80 | 54 | 0.38 | 7.07x10^-01^ | 120,214,270 |
| rs1935621 | 0.59 | 69 | 0.46 | 6.49x10^-01^ | 120,217,663 |
| rs10818152 | 0.46 | 59 | 0.45 | 6.57x10^-01^ | 120,227,075 |
| rs12377720 | 0.16 | 47 | 0.34 | 7.34x10^-01^ | 120,236,223 |
| rs1342481 | 0.65 | 65 | 0.34 | 7.32x10^-01^ | 120,239,806 |
| rs7022505 | 0.91 | 31 | 0.43 | 6.67x10^-01^ | 120,252,970 |
| rs1418501 | 0.55 | 69 | 0.42 | 6.76x10^-01^ | 120,258,540 |
| rs10818158 | 0.76 | 50 | 1.03 | 3.02x10^-01^ | 120,277,179 |
| rs7030074 | 0.55 | 65 | 1.04 | 2.99x10^-01^ | 120,280,906 |
| rs7858444 | 0.83 | 42 | 1.25 | 2.10x10^-01^ | 120,284,690 |
| rs10818161 | 0.92 | 27 | 0.05 | 9.64x10^-01^ | 120,293,663 |
| rs3935562 | 0.56 | 62 | 0.69 | 4.93x10^-01^ | 120,315,542 |
| rs4132476 | 0.84 | 41 | 1.66 | 9.74x10^-02^ | 120,318,112 |
| rs4412426 | 0.62 | 59 | 0.35 | 7.24x10^-01^ | 120,339,931 |
| rs10818184 | 0.21 | 47 | 0.38 | 7.05x10^-01^ | 120,341,541 |
| rs1360810 | 0.16 | 42 | 0.37 | 7.12x10^-01^ | 120,360,115 |
| rs1335258 | 0.67 | 60 | 0.58 | 5.63x10^-01^ | 120,367,144 |
| rs1572299 | 0.46 | 65 | 0.65 | 5.18x10^-01^ | 120,386,238 |
| rs10818193 | 0.76 | 54 | 0.85 | 3.94x10^-01^ | 120,389,513 |
| rs1414097 | 0.36 | 64 | 0.16 | 8.72x10^-01^ | 120,399,513 |
| rs10984116 | 0.10 | 31 | 1.46 | 1.46x10^-01^ | 120,404,373 |
| rs10739518 | 0.26 | 52 | 1.81 | 7.04x10^-02^ | 120,408,741 |
| rs1335245 | 0.64 | 62 | 0.42 | 6.76x10^-01^ | 120,410,665 |
| rs7859977 | 0.54 | 64 | 0.28 | 7.79x10^-01^ | 120,418,206 |
| rs1335248 | 0.71 | 63 | 1.46 | 1.46x10^-01^ | 120,420,218 |
| rs2050418 | 0.43 | 60 | 0.64 | 5.24x10^-01^ | 120,425,372 |
| rs1360809 | 0.96 | 14 | 1.03 | 3.03x10^-01^ | 120,439,050 |
| rs7860560 | 0.71 | 64 | 1.55 | 1.22x10^-01^ | 120,456,062 |
| rs2292244 | 0.27 | 58 | 1.40 | 1.61x10^-01^ | 120,473,419 |
| rs12351498 | 0.97 | 13 | 0.32 | 7.52x10^-01^ | 120,475,287 |
| rs10118723 | 0.22 | 53 | 0.12 | 9.03x10^-01^ | 120,479,346 |
| rs2416682 | 0.48 | 64 | 2.11 | 3.51x10^-02^ | 120,491,190 |
| rs3750473 | 0.21 | 50 | 0.70 | 4.87x10^-01^ | 120,508,103 |
| rs1355031 | 0.31 | 63 | 0.16 | 8.70x10^-01^ | 120,510,023 |
| rs10513342 | 0.72 | 57 | 0.68 | 4.96x10^-01^ | 120,522,154 |
| rs965853 | 0.49 | 55 | 0.14 | 8.92x10^-01^ | 120,541,072 |
| rs906588 | 0.27 | 55 | 0.55 | 5.84x10^-01^ | 120,543,022 |
| rs7849252 | 0.28 | 56 | 1.33 | 1.85x10^-01^ | 120,556,698 |
| rs2102422 | 0.51 | 54 | 0.37 | 7.14x10^-01^ | 120,570,778 |
| rs2900156 | 0.81 | 42 | 0.42 | 6.77x10^-01^ | 120,585,043 |
| rs1326625 | 0.88 | 34 | 0.29 | 7.70x10^-01^ | 120,586,209 |
| rs1571093 | 0.60 | 55 | 1.32 | 1.86x10^-01^ | 120,586,808 |
| rs1409550 | 0.18 | 44 | 1.03 | 3.05x10^-01^ | 120,599,811 |
| rs7025578 | 0.77 | 53 | 2.27 | 2.35x10^-02^ | 120,617,302 |
| rs7048316 | 0.80 | 44 | 1.69 | 9.11x10^-02^ | 120,732,024 |
| rs12237612 | 0.11 | 29 | 0.28 | 7.77x10^-01^ | 120,738,535 |
| rs10984338 | 0.10 | 27 | 2.10 | 3.57x10^-02^ | 120,758,739 |
| rs2807727 | 0.68 | 56 | 0.35 | 7.26x10^-01^ | 120,773,175 |
| rs2818311 | 0.83 | 39 | 0.91 | 3.61x10^-01^ | 120,778,558 |
| rs2818294 | 0.03 | 8 | *** | *** | 120,799,388 |
| rs2818334 | 0.10 | 30 | 0.47 | 6.39x10^-01^ | 120,802,762 |
| rs2807722 | 0.28 | 44 | 0.08 | 9.39x10^-01^ | 120,809,824 |
| rs4836741 | 0.56 | 54 | 0.56 | 5.75x10^-01^ | 120,822,817 |
| rs1017111 | 0.47 | 66 | 1.50 | 1.34x10^-01^ | 120,829,759 |
| rs10818272 | 0.71 | 58 | 0.79 | 4.27x10^-01^ | 120,845,060 |
| rs1158809 | 0.15 | 41 | 2.06 | 3.97x10^-02^ | 120,849,439 |
| rs12554041 | 0.78 | 48 | 0.97 | 3.34x10^-01^ | 120,871,551 |
| rs10491530 | 0.34 | 61 | 0.05 | 9.64x10^-01^ | 120,878,240 |
| rs737640 | 0.67 | 58 | 0.91 | 3.64x10^-01^ | 120,880,212 |
| rs7867690 | 0.10 | 27 | 0.82 | 4.11x10^-01^ | 120,882,087 |
| rs7018768 | 0.46 | 62 | 0.12 | 9.01x10^-01^ | 120,884,147 |
| rs726468 | 0.61 | 67 | 0.59 | 5.56x10^-01^ | 120,888,832 |
| rs230146 | 0.80 | 52 | 0.17 | 8.66x10^-01^ | 120,916,787 |
| rs7468009 | 0.82 | 53 | 1.60 | 1.10x10^-01^ | 120,944,973 |
| rs230109 | 0.51 | 72 | 0.76 | 4.45x10^-01^ | 120,961,408 |
| rs2286791 | 0.79 | 59 | 2.21 | 2.70x10^-02^ | 120,964,310 |
| rs1043379 | 0.92 | 20 | 0.59 | 5.53x10^-01^ | 120,970,128 |
| rs230150 | 0.16 | 38 | 1.36 | 1.74x10^-01^ | 120,986,331 |
| rs230089 | 0.18 | 42 | 0.83 | 4.09x10^-01^ | 120,995,897 |
| rs914652 | 0.21 | 53 | 0.32 | 7.47x10^-01^ | 121,005,192 |
| rs1332455 | 0.63 | 62 | 0.55 | 5.86x10^-01^ | 121,010,240 |
| rs2183963 | 0.42 | 61 | 0.62 | 5.36x10^-01^ | 121,013,839 |
| rs7045999 | 0.86 | 45 | 1.40 | 1.61x10^-01^ | 121,016,745 |
| rs10984446 | 0.21 | 47 | 0.34 | 7.34x10^-01^ | 121,024,128 |
| rs990029 | 0.46 | 70 | 2.00 | 4.57x10^-02^ | 121,032,507 |
| rs12378647 | 0.69 | 53 | 2.08 | 3.72x10^-02^ | 121,041,535 |
| rs1571903 | 0.49 | 67 | 2.20 | 2.80x10^-02^ | 121,047,881 |
| rs539054 | 0.77 | 56 | 0.14 | 8.92x10^-01^ | 121,053,848 |
| rs765276 | 0.67 | 57 | 0.06 | 9.56x10^-01^ | 121,074,674 |
| rs487240 | 0.55 | 53 | 0.32 | 7.52x10^-01^ | 121,075,956 |
| rs914651 | 0.23 | 49 | 0.43 | 6.70x10^-01^ | 121,076,814 |
| rs792932 | 0.58 | 53 | 0.22 | 8.24x10^-01^ | 121,084,282 |
| rs1221147 | 0.87 | 38 | 0.45 | 6.57x10^-01^ | 121,086,370 |
| rs543843 | 0.69 | 57 | 1.15 | 2.50x10^-01^ | 121,094,969 |
| rs10760033 | 0.58 | 56 | 0.82 | 4.14x10^-01^ | 121,109,473 |
| rs1324619 | 0.08 | 28 | 0.22 | 8.23x10^-01^ | 121,112,502 |
| rs10118663 | 0.91 | 30 | 0.50 | 6.17x10^-01^ | 121,121,639 |
| rs1359359 | 0.69 | 51 | 0.18 | 8.59x10^-01^ | 121,125,759 |
| rs7039577 | 0.54 | 66 | 0.66 | 5.08x10^-01^ | 121,157,935 |
| rs4540472 | 0.56 | 59 | 1.78 | 7.47x10^-02^ | 121,160,376 |
| rs942515 | 0.75 | 46 | 1.53 | 1.25x10^-01^ | 121,164,610 |
| rs1324628 | 0.58 | 56 | 0.25 | 8.04x10^-01^ | 121,172,145 |
| rs7020462 | 0.87 | 34 | 0.14 | 8.86x10^-01^ | 121,172,926 |
| rs2025501 | 0.59 | 69 | 0.45 | 6.53x10^-01^ | 121,180,607 |
| rs2210379 | 0.70 | 60 | 2.27 | 2.30x10^-02^ | 121,186,224 |
| rs1331616 | 0.21 | 51 | 2.43 | 1.49x10^-02^ | 121,187,482 |
| rs1331618 | 0.72 | 49 | 2.58 | 9.82x10^-03^ | 121,199,088 |
| rs1331619 | 0.51 | 61 | 0.58 | 5.65x10^-01^ | 121,199,229 |
| rs956172 | 0.19 | 50 | 2.17 | 3.01x10^-02^ | 121,203,928 |
| rs955236 | 0.53 | 58 | 0.51 | 6.13x10^-01^ | 121,208,694 |
| rs1331621 | 0.38 | 60 | 0.10 | 9.22x10^-01^ | 121,214,183 |
| rs10984523 | 0.16 | 40 | 2.59 | 9.62x10^-03^ | 121,219,528 |
| rs10123958 | 0.84 | 39 | 2.10 | 3.62x10^-02^ | 121,222,402 |
| rs9299259 | 0.94 | 24 | 0.39 | 6.95x10^-01^ | 121,228,986 |
| rs2416699 | 0.85 | 41 | 1.28 | 2.00x10^-01^ | 121,236,561 |
| rs1331623 | 0.31 | 59 | 1.07 | 2.85x10^-01^ | 121,239,024 |
| rs1331624 | 0.34 | 59 | 1.02 | 3.08x10^-01^ | 121,244,625 |
| rs1929522 | 0.42 | 52 | 0.57 | 5.72x10^-01^ | 121,251,596 |
| rs10984538 | 0.43 | 57 | 0.16 | 8.70x10^-01^ | 121,257,751 |
| rs12345473 | 0.51 | 55 | 0.63 | 5.29x10^-01^ | 121,259,296 |
| rs1331591 | 0.15 | 40 | 2.58 | 9.89x10^-03^ | 121,266,247 |
| rs7865215 | 0.79 | 45 | 2.54 | 1.10x10^-02^ | 121,282,518 |
| rs7867634 | 0.41 | 56 | 0.33 | 7.39x10^-01^ | 121,286,420 |
| rs1412055 | 0.13 | 43 | 0.41 | 6.85x10^-01^ | 121,289,786 |
| rs6478412 | 0.77 | 48 | 2.81 | 4.91x10^-03^ | 121,293,488 |
| rs10491524 | 0.69 | 50 | 3.14 | 1.68x10^-03^ | 121,297,075 |
| rs10984568 | 0.44 | 60 | 1.75 | 8.01x10^-02^ | 121,300,570 |
| rs10491522 | 0.39 | 64 | 0.37 | 7.08x10^-01^ | 121,304,534 |
| rs10818328 | 0.61 | 66 | 0.34 | 7.36x10^-01^ | 121,319,242 |
| rs1412059 | 0.59 | 67 | 0.48 | 6.31x10^-01^ | 121,324,387 |
| rs1412060 | 0.48 | 65 | 0.07 | 9.46x10^-01^ | 121,329,170 |
| rs1331607 | 0.49 | 65 | 0.07 | 9.45x10^-01^ | 121,339,111 |
| rs1331610 | 0.51 | 66 | 0.05 | 9.63x10^-01^ | 121,341,845 |
| rs1014411 | 0.25 | 48 | 0.66 | 5.12x10^-01^ | 121,359,741 |
| rs10818334 | 0.52 | 63 | 0.74 | 4.57x10^-01^ | 121,373,682 |
| rs4412427 | 0.80 | 49 | 0.79 | 4.32x10^-01^ | 121,381,738 |
| rs10818348 | 0.51 | 59 | 0.60 | 5.52x10^-01^ | 121,442,580 |
| rs10818363 | 0.71 | 58 | 0.42 | 6.75x10^-01^ | 121,475,252 |
| rs1860670 | 0.37 | 66 | 0.65 | 5.16x10^-01^ | 121,477,542 |
| rs6415829 | 0.52 | 54 | 0.05 | 9.63x10^-01^ | 121,482,326 |
| rs960322 | 0.89 | 28 | 0.40 | 6.90x10^-01^ | 121,485,172 |
| rs10984642 | 0.86 | 39 | 0.24 | 8.09x10^-01^ | 121,490,161 |
| rs10818365 | 0.48 | 55 | 0.18 | 8.55x10^-01^ | 121,494,236 |
| rs4477101 | 0.21 | 56 | 0.49 | 6.22x10^-01^ | 121,501,729 |
| rs4330759 | 0.48 | 72 | 0.59 | 5.54x10^-01^ | 121,508,206 |
| rs10818367 | 0.71 | 61 | 0.23 | 8.15x10^-01^ | 121,509,355 |
| rs7855156 | 0.77 | 51 | 1.24 | 2.15x10^-01^ | 121,537,140 |
| rs7030915 | 0.41 | 62 | 0.12 | 9.06x10^-01^ | 121,546,773 |
| rs12552428 | 0.15 | 43 | 2.48 | 1.32x10^-02^ | 121,562,752 |
| rs10513351 | 0.28 | 53 | 0.32 | 7.53x10^-01^ | 121,566,595 |
| rs2781116 | 0.63 | 56 | 0.10 | 9.19x10^-01^ | 121,572,891 |
| rs2809898 | 0.50 | 70 | 0.11 | 9.16x10^-01^ | 121,573,531 |
| rs2781113 | 0.45 | 66 | 0.50 | 6.18x10^-01^ | 121,577,328 |
| rs2809899 | 0.63 | 63 | 0.02 | 9.85x10^-01^ | 121,578,027 |
| rs2781107 | 0.41 | 65 | 0.98 | 3.29x10^-01^ | 121,586,063 |
| rs2781105 | 0.34 | 58 | 0.30 | 7.62x10^-01^ | 121,589,904 |
| rs2781102 | 0.45 | 66 | 1.77 | 7.69x10^-02^ | 121,598,205 |
| rs1333903 | 0.42 | 64 | 1.06 | 2.88x10^-01^ | 121,600,083 |
| rs1413324 | 0.27 | 57 | 0.56 | 5.77x10^-01^ | 121,600,981 |
| rs2151641 | 0.20 | 47 | 2.23 | 2.60x10^-02^ | 121,606,180 |
| rs2184221 | 0.46 | 66 | 0.77 | 4.40x10^-01^ | 121,606,307 |
| rs1889274 | 0.28 | 62 | 3.01 | 2.61x10^-03^ | 121,611,149 |
| rs1413316 | 0.32 | 65 | 2.04 | 4.18x10^-02^ | 121,617,610 |
| rs1333914 | 0.61 | 64 | 1.58 | 1.15x10^-01^ | 121,623,055 |
| rs944956 | 0.26 | 57 | 1.24 | 2.15x10^-01^ | 121,636,414 |
| rs10121662 | 0.79 | 49 | 0.85 | 3.98x10^-01^ | 121,642,899 |
| rs944955 | 0.29 | 55 | 0.77 | 4.43x10^-01^ | 121,643,984 |
| rs2225659 | 0.48 | 68 | 0.18 | 8.58x10^-01^ | 121,648,513 |
| rs7857834 | 0.42 | 59 | 0.61 | 5.44x10^-01^ | 121,660,921 |
| rs10984725 | 0.69 | 55 | 0.52 | 6.07x10^-01^ | 121,663,634 |
| rs9299268 | 0.64 | 63 | 1.12 | 2.61x10^-01^ | 121,664,496 |
| rs1360690 | 0.27 | 52 | 0.84 | 4.02x10^-01^ | 121,664,578 |
| rs10984729 | 0.11 | 37 | 0.30 | 7.65x10^-01^ | 121,665,047 |
| rs2151642 | 0.42 | 67 | 0.61 | 5.40x10^-01^ | 121,697,808 |
| rs1537510 | 0.67 | 62 | 0.71 | 4.80x10^-01^ | 121,716,359 |
| rs6478444 | 0.64 | 49 | 0.39 | 6.96x10^-01^ | 121,721,401 |
| rs1333924 | 0.91 | 25 | 0.71 | 4.80x10^-01^ | 121,735,187 |
| rs949900 | 0.23 | 45 | 0.32 | 7.47x10^-01^ | 121,741,056 |
| rs2416729 | 0.31 | 63 | 1.74 | 8.24x10^-02^ | 121,773,054 |
| rs2416733 | 0.62 | 66 | 2.86 | 4.29x10^-03^ | 121,784,551 |
| rs4837690 | 0.40 | 66 | 0.39 | 6.96x10^-01^ | 121,788,996 |
| rs2416734 | 0.30 | 51 | 1.61 | 1.07x10^-01^ | 121,793,325 |
| rs2416735 | 0.31 | 61 | 1.21 | 2.27x10^-01^ | 121,803,045 |
| rs10984789 | 0.28 | 57 | 0.15 | 8.77x10^-01^ | 121,805,925 |
| rs2416737 | 0.49 | 67 | 0.53 | 6.00x10^-01^ | 121,809,358 |
| rs2416738 | 0.64 | 67 | 0.12 | 9.03x10^-01^ | 121,813,543 |
| rs10818418 | 0.81 | 44 | 0.64 | 5.25x10^-01^ | 121,820,770 |
| rs4477103 | 0.21 | 42 | 1.03 | 3.05x10^-01^ | 121,824,147 |
| rs4837694 | 0.13 | 38 | 0.17 | 8.68x10^-01^ | 121,827,253 |
| rs2900168 | 0.34 | 57 | 0.42 | 6.72x10^-01^ | 121,840,185 |
| rs945248 | 0.39 | 57 | 1.12 | 2.63x10^-01^ | 121,843,550 |
| rs10984814 | 0.18 | 43 | 0.68 | 4.96x10^-01^ | 121,844,278 |
| rs4836797 | 0.59 | 62 | 1.33 | 1.85x10^-01^ | 121,850,668 |
| rs4837698 | 0.19 | 43 | 0.55 | 5.83x10^-01^ | 121,873,289 |
| rs10818425 | 0.16 | 39 | 0.64 | 5.25x10^-01^ | 121,874,755 |
| rs945249 | 0.18 | 41 | 1.37 | 1.70x10^-01^ | 121,882,081 |
| rs4837702 | 0.42 | 59 | 0.52 | 6.05x10^-01^ | 121,888,230 |
| rs12235613 | 0.37 | 58 | 0.10 | 9.17x10^-01^ | 121,892,670 |
| rs7040395 | 0.65 | 63 | 0.34 | 7.37x10^-01^ | 121,903,114 |
| rs10984840 | 0.29 | 68 | 0.92 | 3.57x10^-01^ | 121,908,609 |
| rs4837707 | 0.51 | 57 | 1.25 | 2.11x10^-01^ | 121,921,152 |
| rs10760078 | 0.73 | 57 | 0.70 | 4.82x10^-01^ | 121,934,621 |
| rs4837714 | 0.72 | 56 | 1.31 | 1.90x10^-01^ | 121,949,211 |
| rs4837719 | 0.73 | 54 | 1.07 | 2.84x10^-01^ | 121,958,087 |
| rs4837729 | 0.25 | 49 | 0.03 | 9.79x10^-01^ | 121,994,372 |
| rs4836809 | 0.36 | 54 | 1.09 | 2.76x10^-01^ | 122,012,438 |
| rs2058485 | 0.27 | 47 | 0.64 | 5.23x10^-01^ | 122,036,241 |
| rs1013324 | 0.15 | 44 | 1.84 | 6.56x10^-02^ | 122,051,509 |
| rs759125 | 0.92 | 27 | 0.30 | 7.68x10^-01^ | 122,081,817 |
| rs11792165 | 0.60 | 58 | 2.07 | 3.83x10^-02^ | 122,113,780 |
| rs2159994 | 0.85 | 40 | 0.18 | 8.61x10^-01^ | 122,114,956 |
| rs1112863 | 0.45 | 60 | 0.13 | 8.95x10^-01^ | 122,162,008 |
| rs928406 | 0.36 | 57 | 0.26 | 7.97x10^-01^ | 122,168,066 |
| rs7848913 | 0.37 | 59 | 1.21 | 2.27x10^-01^ | 122,172,263 |
| rs4836817 | 0.29 | 61 | 0.65 | 5.19x10^-01^ | 122,175,193 |
| rs914591 | 0.05 | 15 | 1.34 | 1.81x10^-01^ | 122,189,756 |
| rs2297453 | 0.23 | 48 | 0.09 | 9.30x10^-01^ | 122,205,981 |
| rs2297454 | 0.71 | 49 | 0.11 | 9.12x10^-01^ | 122,211,576 |
| rs2900169 | 0.67 | 57 | 0.11 | 9.15x10^-01^ | 122,225,545 |
| rs10818456 | 0.95 | 18 | 1.40 | 1.60x10^-01^ | 122,249,297 |
| rs7869290 | 0.38 | 68 | 0.35 | 7.25x10^-01^ | 122,265,747 |
| rs10818464 | 0.70 | 62 | 1.23 | 2.20x10^-01^ | 122,353,818 |
| rs10616 | 0.31 | 54 | 0.91 | 3.61x10^-01^ | 122,403,354 |
| rs1359328 | 0.22 | 43 | 1.05 | 2.93x10^-01^ | 122,410,643 |
| rs1359329 | 0.14 | 41 | 1.38 | 1.69x10^-01^ | 122,410,806 |
| rs7861679 | 0.32 | 54 | 0.22 | 8.28x10^-01^ | 122,415,410 |
| rs1324475 | 0.49 | 65 | 1.24 | 2.15x10^-01^ | 122,423,730 |
| rs10760112 | 0.30 | 56 | 0.65 | 5.15x10^-01^ | 122,507,391 |
| rs10985016 | 0.84 | 44 | 0.48 | 6.29x10^-01^ | 122,538,322 |
| rs10739575 | 0.19 | 51 | 1.35 | 1.76x10^-01^ | 122,645,922 |
| rs10985054 | 0.84 | 46 | 0.89 | 3.75x10^-01^ | 122,650,568 |
| rs1837 | 0.27 | 51 | 0.89 | 3.73x10^-01^ | 122,658,050 |
| rs914842 | 0.23 | 49 | 1.14 | 2.54x10^-01^ | 122,658,792 |
| rs10985068 | 0.88 | 38 | 0.74 | 4.58x10^-01^ | 122,669,545 |
| rs1056567 | 0.32 | 58 | 0.97 | 3.30x10^-01^ | 122,671,866 |
| rs2270231 | 0.31 | 47 | 0.81 | 4.19x10^-01^ | 122,690,803 |
| rs2109897 | 0.09 | 24 | 1.44 | 1.51x10^-01^ | 122,764,329 |
| rs4837805 | 0.39 | 53 | 0.49 | 6.23x10^-01^ | 122,786,076 |
| rs7872790 | 0.15 | 43 | 1.16 | 2.44x10^-01^ | 122,787,974 |
| rs2159776 | 0.46 | 59 | 1.35 | 1.76x10^-01^ | 122,795,981 |
| rs7040033 | 0.53 | 64 | 1.39 | 1.64x10^-01^ | 122,798,865 |
| rs992670 | 0.47 | 48 | 0.64 | 5.24x10^-01^ | 122,821,591 |
| rs7033790 | 0.23 | 45 | 0.48 | 6.35x10^-01^ | 122,828,213 |
| rs3815467 | 0.15 | 43 | 1.31 | 1.92x10^-01^ | 122,832,727 |
| rs1468673 | 0.38 | 54 | 0.96 | 3.39x10^-01^ | 122,849,711 |
| rs10985148 | 0.85 | 43 | 0.58 | 5.59x10^-01^ | 122,888,658 |
| rs7022941 | 0.54 | 62 | 1.32 | 1.87x10^-01^ | 122,907,291 |
| rs4836840 | 0.38 | 62 | 1.12 | 2.61x10^-01^ | 122,954,413 |
| rs2146836 | 0.30 | 54 | 1.19 | 2.35x10^-01^ | 122,970,117 |
| rs2302498 | 0.38 | 58 | 1.40 | 1.61x10^-01^ | 122,976,150 |
| rs2296077 | 0.39 | 57 | 1.67 | 9.46x10^-02^ | 122,984,764 |
| rs942152 | 0.40 | 58 | 1.52 | 1.29x10^-01^ | 122,991,506 |
| rs9409230 | 0.05 | 18 | 2.06 | 3.94x10^-02^ | 123,007,581 |
| rs10739592 | 0.50 | 62 | 1.36 | 1.75x10^-01^ | 123,011,433 |
| rs2078141 | 0.54 | 68 | 1.41 | 1.59x10^-01^ | 123,013,845 |
| rs4837817 | 0.16 | 41 | 0.68 | 4.97x10^-01^ | 123,034,984 |
| rs10818517 | 0.51 | 68 | 1.40 | 1.61x10^-01^ | 123,044,420 |
| rs4837833 | 0.33 | 60 | 0.53 | 5.96x10^-01^ | 123,098,868 |
| rs3761856 | 0.53 | 73 | 1.31 | 1.89x10^-01^ | 123,100,125 |
| rs306784 | 0.36 | 58 | 1.27 | 2.05x10^-01^ | 123,112,473 |
| rs306761 | 0.33 | 64 | 1.73 | 8.34x10^-02^ | 123,115,365 |
| rs306772 | 0.18 | 43 | 2.46 | 1.40x10^-02^ | 123,132,176 |
| rs767769 | 0.56 | 59 | 1.74 | 8.14x10^-02^ | 123,138,157 |
| rs306793 | 0.16 | 44 | 0.17 | 8.63x10^-01^ | 123,148,843 |
| rs306798 | 0.69 | 61 | 0.02 | 9.81x10^-01^ | 123,167,868 |
| rs16852 | 0.36 | 61 | 0.05 | 9.61x10^-01^ | 123,169,730 |
| rs306794 | 0.04 | 10 | 1.44 | 1.50x10^-01^ | 123,192,760 |
| rs2196311 | 0.16 | 41 | 0.55 | 5.86x10^-01^ | 123,210,311 |
| rs7390182 | 0.60 | 61 | 1.50 | 1.33x10^-01^ | 123,211,890 |
| rs1369443 | 0.42 | 64 | 0.27 | 7.89x10^-01^ | 123,237,713 |
| rs4837853 | 0.47 | 66 | 0.40 | 6.91x10^-01^ | 123,253,783 |
| rs10118890 | 0.69 | 57 | 0.03 | 9.78x10^-01^ | 123,288,330 |
| rs10818563 | 0.73 | 61 | 0.27 | 7.91x10^-01^ | 123,323,885 |
| rs1964930 | 0.48 | 58 | 0.42 | 6.76x10^-01^ | 123,346,396 |
| rs657239 | 0.19 | 49 | 0.64 | 5.23x10^-01^ | 123,369,962 |
| rs656328 | 0.74 | 62 | 1.35 | 1.78x10^-01^ | 123,370,159 |
| rs476807 | 0.36 | 68 | 2.37 | 1.77x10^-02^ | 123,377,526 |
| rs585260 | 0.36 | 66 | 2.15 | 3.16x10^-02^ | 123,378,686 |
| rs914388 | 0.51 | 69 | 0.56 | 5.73x10^-01^ | 123,383,127 |
| rs10513376 | 0.30 | 56 | 0.55 | 5.85x10^-01^ | 123,394,647 |
| rs12552375 | 0.71 | 62 | 0.16 | 8.71x10^-01^ | 123,396,764 |
| rs2777318 | 0.17 | 40 | 0.70 | 4.86x10^-01^ | 123,408,079 |
| rs2789875 | 0.91 | 21 | 2.00 | 4.59x10^-02^ | 123,424,468 |
| rs514823 | 0.20 | 55 | 2.32 | 2.03x10^-02^ | 123,429,574 |
| rs1106006 | 0.26 | 55 | 0.93 | 3.52x10^-01^ | 123,434,318 |
| rs871214 | 0.65 | 63 | 0.56 | 5.77x10^-01^ | 123,434,396 |
| rs555996 | 0.28 | 58 | 0.83 | 4.06x10^-01^ | 123,436,132 |
| rs1003016 | 0.38 | 55 | 1.29 | 1.96x10^-01^ | 123,443,141 |
| rs10818577 | 0.24 | 54 | 1.37 | 1.70x10^-01^ | 123,453,012 |
| rs1571805 | 0.79 | 47 | 0.61 | 5.41x10^-01^ | 123,462,579 |
| rs1571801 | 0.78 | 50 | 0.60 | 5.48x10^-01^ | 123,467,194 |
| rs7875955 | 0.37 | 60 | 0.95 | 3.43x10^-01^ | 123,478,553 |
| rs870556 | 0.37 | 64 | 1.26 | 2.09x10^-01^ | 123,483,930 |
| rs10985354 | 0.49 | 57 | 0.89 | 3.73x10^-01^ | 123,491,526 |
| rs7875345 | 0.26 | 47 | 0.69 | 4.89x10^-01^ | 123,518,897 |
| rs7020508 | 0.60 | 65 | 1.16 | 2.48x10^-01^ | 123,582,937 |
| rs10985376 | 0.50 | 61 | 0.30 | 7.65x10^-01^ | 123,590,058 |
| rs10985387 | 0.58 | 57 | 0.23 | 8.16x10^-01^ | 123,599,611 |
| rs10818593 | 0.22 | 45 | 1.07 | 2.84x10^-01^ | 123,600,674 |
| rs10760187 | 0.50 | 61 | 0.03 | 9.77x10^-01^ | 123,605,641 |
| rs3923223 | 0.71 | 55 | 0.20 | 8.39x10^-01^ | 123,615,888 |
| rs4074968 | 0.71 | 48 | 1.38 | 1.67x10^-01^ | 123,621,582 |
| rs4837892 | 0.68 | 55 | 0.79 | 4.31x10^-01^ | 123,628,125 |
| rs10818600 | 0.68 | 58 | 0.42 | 6.73x10^-01^ | 123,629,630 |
| rs10760193 | 0.57 | 67 | 0.03 | 9.77x10^-01^ | 123,645,717 |
| rs4836865 | 0.30 | 56 | 0.80 | 4.25x10^-01^ | 123,660,542 |
| rs10739600 | 0.32 | 52 | 0.30 | 7.65x10^-01^ | 123,663,694 |
| rs10513383 | 0.88 | 25 | 1.31 | 1.89x10^-01^ | 123,667,748 |
| rs4503172 | 0.38 | 51 | 0.75 | 4.53x10^-01^ | 123,680,662 |
| rs7033700 | 0.45 | 55 | 0.44 | 6.59x10^-01^ | 123,682,916 |
| rs7871736 | 0.49 | 63 | 0.07 | 9.42x10^-01^ | 123,690,432 |
| rs4304393 | 0.50 | 56 | 0.45 | 6.53x10^-01^ | 123,705,191 |
| rs4128247 | 0.92 | 22 | 0.90 | 3.69x10^-01^ | 123,709,594 |
| rs2003251 | 0.84 | 39 | 0.12 | 9.04x10^-01^ | 123,711,999 |
| rs9408931 | 0.69 | 60 | 0.56 | 5.73x10^-01^ | 123,715,498 |
| rs2416837 | 0.92 | 24 | 0.61 | 5.45x10^-01^ | 123,720,188 |
| rs7022103 | 0.84 | 42 | 0.27 | 7.86x10^-01^ | 123,732,934 |
| rs13295532 | 0.81 | 48 | 1.20 | 2.30x10^-01^ | 123,737,698 |
| rs10985458 | 0.74 | 57 | 1.42 | 1.55x10^-01^ | 123,740,986 |
| rs2416847 | 0.75 | 54 | 0.90 | 3.66x10^-01^ | 123,753,373 |
| rs2416843 | 0.71 | 61 | 0.93 | 3.55x10^-01^ | 123,763,781 |
| rs4534187 | 0.46 | 53 | 2.02 | 4.34x10^-02^ | 123,775,902 |
| rs13298566 | 0.66 | 53 | 1.80 | 7.19x10^-02^ | 123,781,065 |
| rs12378199 | 0.67 | 56 | 1.61 | 1.07x10^-01^ | 123,793,653 |
| rs10985478 | 0.78 | 46 | 0.95 | 3.41x10^-01^ | 123,799,252 |
| rs12341511 | 0.64 | 56 | 1.61 | 1.08x10^-01^ | 123,803,816 |
| rs10818620 | 0.72 | 52 | 1.14 | 2.55x10^-01^ | 123,843,081 |
| rs7045950 | 0.76 | 48 | 1.11 | 2.66x10^-01^ | 123,891,204 |
| rs7860331 | 0.65 | 59 | 1.80 | 7.22x10^-02^ | 123,892,107 |
| rs10985526 | 0.64 | 62 | 0.74 | 4.60x10^-01^ | 123,902,841 |
| rs12551572 | 0.68 | 61 | 1.01 | 3.13x10^-01^ | 123,904,206 |
| rs10985530 | 0.74 | 57 | 0.31 | 7.60x10^-01^ | 123,909,168 |
| rs944214 | 0.66 | 63 | 1.39 | 1.65x10^-01^ | 123,911,832 |
| rs10760209 | 0.74 | 55 | 0.73 | 4.69x10^-01^ | 123,915,640 |
| rs10125524 | 0.32 | 62 | 0.17 | 8.68x10^-01^ | 123,935,748 |
| rs1888215 | 0.76 | 52 | 0.49 | 6.22x10^-01^ | 123,938,472 |
| rs6822 | 0.16 | 36 | 0.25 | 8.05x10^-01^ | 123,946,277 |
| rs3793616 | 0.43 | 68 | 1.87 | 6.15x10^-02^ | 123,950,403 |
| rs4679 | 0.42 | 66 | 1.72 | 8.52x10^-02^ | 123,954,434 |
| rs950019 | 0.50 | 59 | 1.24 | 2.17x10^-01^ | 123,965,426 |
| rs1411444 | 0.51 | 60 | 0.96 | 3.37x10^-01^ | 123,969,032 |
| rs10985557 | 0.45 | 59 | 0.43 | 6.71x10^-01^ | 123,974,977 |
| rs1010804 | 0.44 | 57 | 0.29 | 7.76x10^-01^ | 123,981,675 |
| rs10760211 | 0.48 | 62 | 0.54 | 5.92x10^-01^ | 123,985,425 |
| rs2039197 | 0.75 | 49 | 1.23 | 2.19x10^-01^ | 123,988,867 |
| rs913933 | 0.39 | 64 | 0.73 | 4.65x10^-01^ | 123,989,359 |
| rs2039196 | 0.16 | 38 | 2.24 | 2.50x10^-02^ | 123,989,422 |
| rs913932 | 0.54 | 55 | 0.23 | 8.17x10^-01^ | 123,989,565 |
| rs1888214 | 0.49 | 67 | 0.86 | 3.91x10^-01^ | 123,992,840 |
| rs870272 | 0.38 | 58 | 0.38 | 7.01x10^-01^ | 123,993,381 |
| rs1571587 | 0.33 | 54 | 0.52 | 6.00x10^-01^ | 123,997,362 |
| rs3750486 | 0.09 | 37 | 0.00 | 1.00x10+00 | 124,006,082 |
| rs3793617 | 0.23 | 53 | 0.46 | 6.45x10^-01^ | 124,006,270 |
| rs10818651 | 0.36 | 66 | 0.00 | 1.00x10+00 | 124,014,240 |
| rs1467737 | 0.47 | 60 | 0.24 | 8.14x10^-01^ | 124,022,321 |
| rs3793621 | 0.30 | 55 | 0.94 | 3.45x10^-01^ | 124,023,946 |
| rs10818652 | 0.58 | 62 | 0.44 | 6.60x10^-01^ | 124,025,606 |
| rs3808901 | 0.30 | 60 | 0.03 | 9.77x10^-01^ | 124,032,492 |
| rs3808900 | 0.21 | 52 | 0.59 | 5.56x10^-01^ | 124,032,585 |
| rs1571585 | 0.43 | 62 | 0.05 | 9.59x10^-01^ | 124,038,046 |
| rs1060586 | 0.50 | 56 | 0.14 | 8.92x10^-01^ | 124,043,500 |
| rs1888218 | 0.77 | 45 | 0.29 | 7.76x10^-01^ | 124,054,296 |
| rs10985579 | 0.42 | 57 | 0.37 | 7.08x10^-01^ | 124,062,113 |
| rs2297177 | 0.53 | 51 | 0.08 | 9.35x10^-01^ | 124,063,795 |
| rs10818675 | 0.12 | 35 | 0.22 | 8.23x10^-01^ | 124,090,024 |
| rs7036419 | 0.10 | 33 | 0.94 | 3.46x10^-01^ | 124,142,415 |
| rs13283472 | 0.12 | 38 | 0.90 | 3.71x10^-01^ | 124,145,611 |
| rs7022025 | 0.08 | 24 | 1.07 | 2.85x10^-01^ | 124,151,774 |
| rs10513401 | 0.25 | 48 | 0.25 | 8.02x10^-01^ | 124,160,170 |
| rs4837960 | 0.86 | 39 | 0.74 | 4.61x10^-01^ | 124,163,792 |
| rs10306108 | 0.95 | 18 | 0.25 | 7.99x10^-01^ | 124,171,301 |
| rs1236913 | 0.93 | 21 | 1.36 | 1.75x10^-01^ | 124,173,300 |
| rs1213266 | 0.92 | 23 | 0.52 | 6.00x10^-01^ | 124,176,705 |
| rs2282169 | 0.21 | 53 | 0.91 | 3.61x10^-01^ | 124,180,517 |
| rs5788 | 0.15 | 39 | 1.04 | 2.97x10^-01^ | 124,183,613 |
| rs5789 | 0.04 | 12 | 1.23 | 2.21x10^-01^ | 124,183,794 |
| rs10306202 | 0.92 | 27 | 0.89 | 3.74x10^-01^ | 124,199,342 |
| rs10513402 | 0.12 | 34 | 0.00 | 1.00x10+00 | 124,204,757 |
| rs950104 | 0.17 | 49 | 0.54 | 5.93x10^-01^ | 124,205,046 |
| rs10818684 | 0.75 | 63 | 1.31 | 1.90x10^-01^ | 124,209,751 |
| rs1234909 | 0.19 | 48 | 0.24 | 8.12x10^-01^ | 124,225,832 |
| rs4557776 | 0.71 | 58 | 0.10 | 9.18x10^-01^ | 124,242,011 |
| rs2778635 | 0.72 | 57 | 0.03 | 9.80x10^-01^ | 124,269,620 |
| rs1332406 | 0.17 | 36 | 0.90 | 3.71x10^-01^ | 124,270,506 |
| rs1888941 | 0.08 | 22 | 0.14 | 8.93x10^-01^ | 124,275,969 |
| rs10818700 | 0.10 | 27 | 0.00 | 1.00x10+00 | 124,280,447 |
| rs7027407 | 0.21 | 49 | 0.31 | 7.61x10^-01^ | 124,289,178 |
| rs2416863 | 0.92 | 27 | 1.05 | 2.94x10^-01^ | 124,292,798 |
| rs2416864 | 0.17 | 48 | 0.30 | 7.67x10^-01^ | 124,293,834 |
| rs10739609 | 0.44 | 70 | 0.33 | 7.42x10^-01^ | 124,305,138 |
| rs10513404 | 0.65 | 51 | 1.66 | 9.71x10^-02^ | 124,307,054 |
| rs1556190 | 0.50 | 61 | 0.92 | 3.56x10^-01^ | 124,308,705 |
| rs7873246 | 0.86 | 34 | 1.28 | 2.01x10^-01^ | 124,310,843 |
| rs7019219 | 0.48 | 53 | 0.56 | 5.77x10^-01^ | 124,311,660 |
| rs7863861 | 0.04 | 14 | 0.78 | 4.33x10^-01^ | 124,319,083 |
| rs4837973 | 0.71 | 62 | 1.05 | 2.95x10^-01^ | 124,324,594 |
| rs4837974 | 0.52 | 60 | 0.24 | 8.12x10^-01^ | 124,332,485 |
| rs1041356 | 0.66 | 57 | 1.44 | 1.51x10^-01^ | 124,344,571 |
| rs1590879 | 0.61 | 59 | 0.31 | 7.53x10^-01^ | 124,352,515 |
| rs1831370 | 0.51 | 62 | 2.19 | 2.88x10^-02^ | 124,355,378 |
| rs1341044 | 0.25 | 51 | 1.97 | 4.92x10^-02^ | 124,356,000 |
| rs1041355 | 0.69 | 61 | 0.91 | 3.63x10^-01^ | 124,357,482 |
| rs10739614 | 0.38 | 57 | 2.77 | 5.61x10^-03^ | 124,369,946 |
| rs1999182 | 0.45 | 55 | 2.15 | 3.13x10^-02^ | 124,370,560 |
| rs7861129 | 0.76 | 49 | 1.57 | 1.16x10^-01^ | 124,373,146 |
| rs2416887 | 0.78 | 59 | 0.10 | 9.20x10^-01^ | 124,375,437 |
| rs10818726 | 0.48 | 65 | 0.39 | 6.97x10^-01^ | 124,402,580 |
| rs960577 | 0.12 | 40 | 0.66 | 5.06x10^-01^ | 124,405,430 |
| rs10513408 | 0.45 | 66 | 0.00 | 1.00x10+00 | 124,407,515 |
| rs10513409 | 0.42 | 67 | 0.36 | 7.20x10^-01^ | 124,409,753 |
| rs2277195 | 0.34 | 58 | 0.35 | 7.25x10^-01^ | 124,411,922 |
| rs1000510 | 0.90 | 30 | 1.13 | 2.57x10^-01^ | 124,412,699 |
| rs4836901 | 0.82 | 33 | 1.17 | 2.44x10^-01^ | 124,415,249 |
| rs972925 | 0.24 | 51 | 0.75 | 4.56x10^-01^ | 124,416,908 |
| rs10818732 | 0.22 | 43 | 0.21 | 8.36x10^-01^ | 124,424,018 |
| rs723258 | 0.27 | 56 | 0.33 | 7.40x10^-01^ | 124,427,004 |
| rs10491515 | 0.37 | 63 | 0.05 | 9.64x10^-01^ | 124,427,674 |
| rs10491516 | 0.71 | 47 | 0.24 | 8.10x10^-01^ | 124,428,147 |
| rs1536929 | 0.33 | 60 | 0.08 | 9.34x10^-01^ | 124,431,190 |
| rs7854941 | 0.94 | 19 | 0.31 | 7.58x10^-01^ | 124,432,427 |
| rs7859683 | 0.55 | 64 | 0.22 | 8.27x10^-01^ | 124,433,984 |
| rs2416891 | 0.20 | 60 | 1.72 | 8.63x10^-02^ | 124,462,245 |
| rs237617 | 0.30 | 60 | 1.14 | 2.55x10^-01^ | 124,462,686 |
| rs70156 | 0.20 | 53 | 0.65 | 5.16x10^-01^ | 124,464,328 |
| rs237622 | 0.87 | 34 | 1.84 | 6.57x10^-02^ | 124,465,381 |
| rs10985750 | 0.32 | 60 | 0.47 | 6.40x10^-01^ | 124,500,774 |
| rs4838006 | 0.96 | 12 | 0.94 | 3.46x10^-01^ | 124,520,577 |
| rs2535741 | 0.16 | 40 | 1.15 | 2.50x10^-01^ | 124,596,539 |
| rs2535742 | 0.14 | 35 | 1.24 | 2.16x10^-01^ | 124,599,681 |
| rs4997232 | 0.22 | 51 | 1.34 | 1.81x10^-01^ | 124,621,994 |
| rs10513413 | 0.74 | 56 | 1.33 | 1.83x10^-01^ | 124,645,399 |
| rs10818754 | 0.78 | 44 | 0.94 | 3.49x10^-01^ | 124,648,448 |
| rs2596703 | 0.89 | 27 | 0.53 | 6.00x10^-01^ | 124,681,671 |
| rs2792999 | 0.19 | 46 | 0.24 | 8.10x10^-01^ | 124,684,843 |
| rs2239540 | 0.90 | 25 | 0.40 | 6.87x10^-01^ | 124,714,816 |
| rs2241064 | 0.13 | 32 | 0.17 | 8.64x10^-01^ | 124,720,368 |
| rs3739835 | 0.95 | 16 | 0.97 | 3.32x10^-01^ | 124,723,469 |
| rs10818768 | 0.46 | 67 | 0.50 | 6.18x10^-01^ | 124,749,144 |
| rs2430398 | 0.91 | 23 | 0.25 | 8.03x10^-01^ | 124,858,277 |
| rs2791437 | 0.20 | 48 | 0.03 | 9.73x10^-01^ | 124,884,403 |
| rs700081 | 0.11 | 28 | 0.44 | 6.57x10^-01^ | 124,901,709 |
| rs803717 | 0.35 | 61 | 0.06 | 9.55x10^-01^ | 124,908,431 |
| rs700085 | 0.08 | 18 | 1.83 | 6.79x10^-02^ | 124,910,287 |
| rs803732 | 0.18 | 40 | 1.11 | 2.67x10^-01^ | 124,921,362 |
| rs7044347 | 0.13 | 30 | 0.17 | 8.62x10^-01^ | 124,928,107 |
| rs4836913 | 0.96 | 14 | 0.65 | 5.18x10^-01^ | 124,937,721 |
| rs7870519 | 0.05 | 11 | 0.23 | 8.19x10^-01^ | 124,957,537 |
| rs7026688 | 0.87 | 35 | 0.82 | 4.13x10^-01^ | 125,015,218 |
| rs3739839 | 0.05 | 17 | 0.00 | 1.00x10+00 | 125,071,671 |
| rs10760271 | 0.11 | 31 | 0.50 | 6.19x10^-01^ | 125,083,831 |
| rs10739624 | 0.60 | 62 | 0.23 | 8.19x10^-01^ | 125,085,845 |
| rs868693 | 0.52 | 65 | 0.89 | 3.73x10^-01^ | 125,089,796 |
| rs10818803 | 0.61 | 65 | 0.40 | 6.90x10^-01^ | 125,096,285 |
| rs10985947 | 0.82 | 44 | 0.51 | 6.10x10^-01^ | 125,098,841 |
| rs10985950 | 0.42 | 65 | 1.12 | 2.62x10^-01^ | 125,102,886 |
| rs4838043 | 0.85 | 35 | 1.19 | 2.34x10^-01^ | 125,117,783 |
| rs10985963 | 0.63 | 54 | 0.19 | 8.50x10^-01^ | 125,121,559 |
| rs7024188 | 0.34 | 59 | 1.60 | 1.09x10^-01^ | 125,122,164 |
| rs876173 | 0.83 | 41 | 0.40 | 6.89x10^-01^ | 125,123,053 |
| rs1971605 | 0.11 | 31 | 1.12 | 2.63x10^-01^ | 125,131,608 |
| rs3932701 | 0.30 | 59 | 0.53 | 5.94x10^-01^ | 125,138,195 |
| rs10114139 | 0.52 | 51 | 0.05 | 9.58x10^-01^ | 125,140,829 |
| rs10760280 | 0.59 | 56 | 1.11 | 2.65x10^-01^ | 125,152,633 |
| rs4838051 | 0.10 | 29 | 2.18 | 2.89x10^-02^ | 125,156,686 |
| rs1891632 | 0.09 | 29 | 0.05 | 9.60x10^-01^ | 125,157,379 |
| rs2808416 | 0.66 | 54 | 0.03 | 9.76x10^-01^ | 125,159,980 |
| rs1105224 | 0.62 | 52 | 0.88 | 3.80x10^-01^ | 125,167,819 |
| rs7034379 | 0.35 | 54 | 0.72 | 4.74x10^-01^ | 125,172,150 |
| rs4836919 | 0.09 | 29 | 0.30 | 7.63x10^-01^ | 125,184,823 |
| rs2797945 | 0.36 | 66 | 1.82 | 6.93x10^-02^ | 125,190,651 |
| rs4466467 | 0.09 | 31 | 0.99 | 3.22x10^-01^ | 125,202,042 |
| rs10818818 | 0.26 | 63 | 1.74 | 8.22x10^-02^ | 125,211,015 |
| rs12347955 | 0.15 | 39 | 1.98 | 4.82x10^-02^ | 125,218,749 |
| rs7851052 | 0.20 | 43 | 1.83 | 6.78x10^-02^ | 125,219,025 |
| rs10124048 | 0.13 | 38 | 1.47 | 1.41x10^-01^ | 125,220,918 |
| rs10733661 | 0.55 | 60 | 0.18 | 8.59x10^-01^ | 125,223,125 |
| rs10818820 | 0.65 | 70 | 0.16 | 8.73x10^-01^ | 125,236,025 |
| rs4240478 | 0.30 | 64 | 0.25 | 7.99x10^-01^ | 125,240,290 |
| rs1110320 | 0.41 | 63 | 0.04 | 9.65x10^-01^ | 125,243,481 |
| rs7859831 | 0.88 | 30 | 0.26 | 7.92x10^-01^ | 125,245,575 |
| rs3829852 | 0.96 | 12 | 0.65 | 5.16x10^-01^ | 125,249,827 |
| rs11999243 | 0.86 | 40 | 0.83 | 4.05x10^-01^ | 125,271,720 |
| rs10818826 | 0.08 | 30 | 0.43 | 6.69x10^-01^ | 125,311,801 |
| rs2772216 | 0.71 | 62 | 0.09 | 9.29x10^-01^ | 125,321,467 |
| rs2772215 | 0.17 | 49 | 0.36 | 7.17x10^-01^ | 125,322,820 |
| rs7026921 | 0.26 | 60 | 0.70 | 4.86x10^-01^ | 125,337,716 |
| rs4836929 | 0.14 | 38 | 0.89 | 3.76x10^-01^ | 125,344,040 |
| rs10513428 | 0.07 | 18 | 1.14 | 2.54x10^-01^ | 125,344,374 |
| rs748994 | 0.06 | 26 | 0.64 | 5.22x10^-01^ | 125,344,516 |
| rs4836931 | 0.13 | 37 | 0.78 | 4.35x10^-01^ | 125,349,693 |
| rs10119667 | 0.20 | 49 | 0.56 | 5.73x10^-01^ | 125,363,871 |
| rs10818846 | 0.89 | 37 | 0.59 | 5.52x10^-01^ | 125,385,926 |
| rs2039152 | 0.27 | 53 | 1.28 | 1.99x10^-01^ | 125,395,463 |
| rs755130 | 0.24 | 51 | 0.95 | 3.43x10^-01^ | 125,412,545 |
| rs2183262 | 0.31 | 58 | 0.96 | 3.36x10^-01^ | 125,419,356 |
| rs7037809 | 0.95 | 15 | 0.43 | 6.68x10^-01^ | 125,423,183 |
| rs1475528 | 0.32 | 62 | 0.99 | 3.22x10^-01^ | 125,439,913 |
| rs7027523 | 0.29 | 55 | 1.49 | 1.37x10^-01^ | 125,463,161 |
| rs4552982 | 0.27 | 54 | 1.25 | 2.11x10^-01^ | 125,467,701 |
| rs7857068 | 0.37 | 61 | 1.28 | 1.99x10^-01^ | 125,475,640 |
| rs10818854 | 0.06 | 23 | 0.15 | 8.84x10^-01^ | 125,486,599 |
| rs10760297 | 0.19 | 36 | 0.08 | 9.37x10^-01^ | 125,498,610 |
| rs4836940 | 0.87 | 22 | 0.17 | 8.67x10^-01^ | 125,507,130 |
| rs7851425 | 0.13 | 42 | 0.84 | 3.99x10^-01^ | 125,516,784 |
| rs7034451 | 0.15 | 29 | 0.28 | 7.83x10^-01^ | 125,518,389 |
| rs4388514 | 0.82 | 49 | 0.83 | 4.10x10^-01^ | 125,520,063 |
| rs10818865 | 0.21 | 43 | 0.66 | 5.09x10^-01^ | 125,549,420 |
| rs12339991 | 0.17 | 46 | 1.06 | 2.91x10^-01^ | 125,550,906 |
| rs2479106 | 0.29 | 63 | 0.78 | 4.37x10^-01^ | 125,565,033 |
| rs2209803 | 0.07 | 27 | 0.58 | 5.65x10^-01^ | 125,566,691 |
| rs1752156 | 0.19 | 52 | 0.24 | 8.12x10^-01^ | 125,574,333 |
| rs2148953 | 0.91 | 29 | 0.59 | 5.58x10^-01^ | 125,575,463 |
| rs1778896 | 0.96 | 10 | 0.96 | 3.36x10^-01^ | 125,596,074 |
| rs1752164 | 0.86 | 43 | 0.07 | 9.48x10^-01^ | 125,598,358 |
| rs1927239 | 0.89 | 39 | 0.28 | 7.78x10^-01^ | 125,635,704 |
| rs7863739 | 0.14 | 39 | 0.33 | 7.41x10^-01^ | 125,641,077 |
| rs591168 | 0.06 | 21 | 0.27 | 7.87x10^-01^ | 125,670,599 |
| rs597189 | 0.89 | 34 | 0.96 | 3.38x10^-01^ | 125,702,745 |
| rs2479102 | 0.29 | 62 | 0.31 | 7.56x10^-01^ | 125,723,776 |
| rs12115299 | 0.94 | 18 | 0.57 | 5.72x10^-01^ | 125,725,719 |
| rs3739827 | 0.80 | 50 | 0.27 | 7.90x10^-01^ | 125,729,978 |
| rs2058432 | 0.76 | 53 | 0.35 | 7.25x10^-01^ | 125,743,559 |
| rs12335840 | 0.23 | 51 | 0.06 | 9.56x10^-01^ | 125,753,232 |
| rs4838084 | 0.26 | 57 | 0.30 | 7.67x10^-01^ | 125,755,096 |
| rs4838085 | 0.30 | 65 | 0.78 | 4.33x10^-01^ | 125,759,931 |
| rs3983857 | 0.34 | 66 | 0.00 | 1.00x10+00 | 125,772,349 |
| rs741031 | 0.12 | 28 | 0.47 | 6.37x10^-01^ | 125,772,609 |
| rs4836950 | 0.52 | 61 | 0.72 | 4.73x10^-01^ | 125,789,452 |
| rs1887343 | 0.63 | 66 | 0.33 | 7.42x10^-01^ | 125,792,750 |
| rs10125297 | 0.47 | 62 | 0.12 | 9.03x10^-01^ | 125,799,570 |
| rs4314746 | 0.49 | 65 | 0.31 | 7.58x10^-01^ | 125,802,949 |
| rs4838092 | 0.51 | 59 | 0.73 | 4.64x10^-01^ | 125,807,248 |
| rs1327291 | 0.48 | 64 | 0.52 | 6.03x10^-01^ | 125,807,869 |
| rs1887341 | 0.55 | 60 | 1.59 | 1.12x10^-01^ | 125,809,112 |
| rs7867942 | 0.07 | 26 | 0.90 | 3.67x10^-01^ | 125,811,142 |
| rs917783 | 0.39 | 61 | 0.47 | 6.40x10^-01^ | 125,830,428 |
| rs1555972 | 0.62 | 58 | 0.49 | 6.22x10^-01^ | 125,836,225 |
| rs4836959 | 0.52 | 69 | 0.05 | 9.62x10^-01^ | 125,839,964 |
| rs6478638 | 0.86 | 34 | 1.72 | 8.60x10^-02^ | 125,849,557 |
| rs4838098 | 0.10 | 27 | 2.08 | 3.78x10^-02^ | 125,854,737 |
| rs10986189 | 0.15 | 43 | 1.00 | 3.15x10^-01^ | 125,858,318 |
| rs2025819 | 0.57 | 64 | 0.48 | 6.28x10^-01^ | 125,864,072 |
| rs3915912 | 0.89 | 31 | 1.42 | 1.56x10^-01^ | 125,871,911 |
| rs3861868 | 0.54 | 71 | 0.57 | 5.66x10^-01^ | 125,875,305 |
| rs1409897 | 0.69 | 48 | 1.53 | 1.27x10^-01^ | 125,886,555 |
| rs7858299 | 0.25 | 56 | 0.68 | 4.97x10^-01^ | 125,895,163 |
| rs7040016 | 0.24 | 53 | 1.76 | 7.90x10^-02^ | 125,898,372 |
| rs7030878 | 0.69 | 61 | 0.17 | 8.69x10^-01^ | 125,903,264 |
| rs2148742 | 0.72 | 51 | 1.33 | 1.83x10^-01^ | 125,910,873 |
| rs2182660 | 0.74 | 50 | 1.32 | 1.86x10^-01^ | 125,911,120 |
| rs4838114 | 0.93 | 19 | 1.26 | 2.07x10^-01^ | 125,915,714 |
| rs1463729 | 0.57 | 60 | 1.71 | 8.73x10^-02^ | 125,921,269 |
| rs2807602 | 0.20 | 41 | 0.84 | 4.02x10^-01^ | 125,928,278 |
| rs4836965 | 0.38 | 62 | 0.63 | 5.26x10^-01^ | 125,929,595 |
| rs2807595 | 0.27 | 56 | 0.27 | 7.84x10^-01^ | 125,931,428 |
| rs2807592 | 0.69 | 57 | 1.72 | 8.48x10^-02^ | 125,933,257 |
| rs2767761 | 0.60 | 51 | 0.74 | 4.58x10^-01^ | 125,935,232 |
| rs2767763 | 0.24 | 44 | 0.64 | 5.25x10^-01^ | 125,938,792 |
| rs10818924 | 0.37 | 62 | 0.42 | 6.72x10^-01^ | 125,953,683 |
| rs2767772 | 0.57 | 66 | 0.29 | 7.72x10^-01^ | 125,962,164 |
| rs7854799 | 0.24 | 52 | 0.58 | 5.65x10^-01^ | 125,969,224 |
| rs9299296 | 0.21 | 47 | 0.62 | 5.38x10^-01^ | 125,981,196 |
| rs7860461 | 0.92 | 22 | 1.27 | 2.06x10^-01^ | 125,986,643 |
| rs4311746 | 0.18 | 39 | 0.00 | 1.00x10+00 | 125,989,034 |
| rs2767777 | 0.74 | 44 | 1.65 | 9.92x10^-02^ | 125,998,894 |
| rs4838126 | 0.86 | 32 | 1.00 | 3.19x10^-01^ | 126,001,723 |
| rs1106489 | 0.08 | 23 | 0.29 | 7.76x10^-01^ | 126,005,217 |
| rs10986271 | 0.23 | 54 | 0.96 | 3.37x10^-01^ | 126,007,954 |
| rs7047184 | 0.90 | 29 | 0.86 | 3.91x10^-01^ | 126,009,400 |
| rs4838130 | 0.90 | 25 | 0.79 | 4.31x10^-01^ | 126,010,991 |
| rs10760332 | 0.80 | 38 | 0.83 | 4.06x10^-01^ | 126,014,843 |
| rs876452 | 0.34 | 58 | 0.42 | 6.77x10^-01^ | 126,018,779 |
| rs7851784 | 0.69 | 50 | 0.06 | 9.49x10^-01^ | 126,021,211 |
| rs953470 | 0.24 | 46 | 0.22 | 8.26x10^-01^ | 126,024,203 |
| rs2150238 | 0.13 | 39 | 0.88 | 3.78x10^-01^ | 126,028,924 |
| rs10818930 | 0.22 | 54 | 1.04 | 2.98x10^-01^ | 126,031,006 |
| rs3949752 | 0.32 | 53 | 0.13 | 8.97x10^-01^ | 126,039,917 |
| rs7861786 | 0.68 | 52 | 0.61 | 5.44x10^-01^ | 126,041,281 |
| rs4838141 | 0.61 | 52 | 0.03 | 9.77x10^-01^ | 126,050,083 |
| rs10986291 | 0.34 | 54 | 0.09 | 9.28x10^-01^ | 126,056,092 |
| rs4838143 | 0.85 | 35 | 0.56 | 5.73x10^-01^ | 126,057,457 |
| rs2065221 | 0.44 | 57 | 0.69 | 4.93x10^-01^ | 126,060,649 |
| rs944339 | 0.55 | 55 | 0.05 | 9.57x10^-01^ | 126,080,799 |
| rs2900220 | 0.40 | 52 | 0.49 | 6.26x10^-01^ | 126,081,991 |
| rs7861040 | 0.65 | 56 | 0.03 | 9.78x10^-01^ | 126,083,956 |
| rs944340 | 0.58 | 57 | 0.32 | 7.48x10^-01^ | 126,100,252 |
| rs2026191 | 0.43 | 55 | 0.48 | 6.33x10^-01^ | 126,102,309 |
| rs3780209 | 0.93 | 23 | 0.12 | 9.03x10^-01^ | 126,106,748 |
| rs4838157 | 0.46 | 55 | 0.74 | 4.58x10^-01^ | 126,107,961 |
| rs4838161 | 0.57 | 57 | 0.83 | 4.08x10^-01^ | 126,119,074 |
| rs912355 | 0.67 | 51 | 0.87 | 3.86x10^-01^ | 126,130,787 |
| rs912353 | 0.62 | 46 | 0.00 | 1.00x10+00 | 126,159,177 |
| rs11789637 | 0.57 | 53 | 0.45 | 6.56x10^-01^ | 126,163,122 |
| rs3758207 | 0.56 | 55 | 0.64 | 5.25x10^-01^ | 126,171,841 |
| rs3780197 | 0.59 | 47 | 0.32 | 7.49x10^-01^ | 126,215,542 |
| rs12343206 | 0.59 | 52 | 0.18 | 8.60x10^-01^ | 126,223,888 |
| rs4838173 | 0.70 | 50 | 0.49 | 6.26x10^-01^ | 126,229,507 |
| rs749147 | 0.02 | 6 | *** | *** | 126,243,203 |
| rs11794849 | 0.15 | 40 | 0.84 | 4.02x10^-01^ | 126,244,533 |
| rs4838177 | 0.59 | 61 | 0.83 | 4.04x10^-01^ | 126,258,079 |
| rs10818973 | 0.63 | 63 | 0.44 | 6.62x10^-01^ | 126,265,340 |
| rs1886124 | 0.51 | 56 | 0.80 | 4.25x10^-01^ | 126,273,028 |
| rs915034 | 0.50 | 53 | 0.41 | 6.83x10^-01^ | 126,284,776 |
| rs7847540 | 0.48 | 54 | 0.45 | 6.53x10^-01^ | 126,294,171 |
| rs10986358 | 0.71 | 55 | 0.06 | 9.51x10^-01^ | 126,334,006 |
| rs4240486 | 0.54 | 52 | 0.52 | 6.04x10^-01^ | 126,380,931 |
| rs10818986 | 0.85 | 43 | 1.34 | 1.81x10^-01^ | 126,510,715 |
| rs7874348 | 0.67 | 53 | 0.44 | 6.63x10^-01^ | 126,606,199 |
| rs10986432 | 0.83 | 37 | 1.73 | 8.36x10^-02^ | 126,610,461 |
| rs1041205 | 0.10 | 21 | 0.41 | 6.86x10^-01^ | 126,620,825 |
| rs12006475 | 0.76 | 53 | 0.61 | 5.44x10^-01^ | 126,643,879 |
| rs10818998 | 0.39 | 69 | 1.19 | 2.36x10^-01^ | 126,650,850 |
| rs10986463 | 0.44 | 68 | 1.26 | 2.06x10^-01^ | 126,652,369 |
| rs876663 | 0.48 | 68 | 1.01 | 3.15x10^-01^ | 126,659,962 |
| rs1051358 | 0.84 | 33 | 0.11 | 9.15x10^-01^ | 126,679,516 |
| rs2241923 | 0.65 | 60 | 0.11 | 9.16x10^-01^ | 126,682,902 |
| rs2304957 | 0.78 | 57 | 0.99 | 3.24x10^-01^ | 126,683,767 |
| rs7040520 | 0.38 | 58 | 1.40 | 1.61x10^-01^ | 126,751,142 |
| rs10986507 | 0.96 | 14 | 0.56 | 5.77x10^-01^ | 126,789,622 |
| rs4837005 | 0.64 | 63 | 1.31 | 1.90x10^-01^ | 126,805,799 |
| rs7869347 | 0.85 | 38 | 0.35 | 7.25x10^-01^ | 126,825,168 |
| rs12352758 | 0.86 | 36 | 0.78 | 4.34x10^-01^ | 126,829,264 |
| rs1328160 | 0.72 | 62 | 0.11 | 9.15x10^-01^ | 126,832,706 |
| rs394750 | 0.57 | 64 | 0.56 | 5.77x10^-01^ | 126,840,886 |
| rs600310 | 0.35 | 65 | 0.03 | 9.80x10^-01^ | 126,855,968 |
| rs633533 | 0.69 | 56 | 0.63 | 5.31x10^-01^ | 126,859,702 |
| rs662621 | 0.66 | 67 | 0.77 | 4.42x10^-01^ | 126,861,599 |
| rs827660 | 0.83 | 43 | 1.35 | 1.78x10^-01^ | 126,865,212 |
| rs656322 | 0.64 | 59 | 1.16 | 2.47x10^-01^ | 126,874,098 |
| rs12377973 | 0.52 | 57 | 0.58 | 5.60x10^-01^ | 126,896,516 |
| rs10986614 | 0.84 | 46 | 0.46 | 6.49x10^-01^ | 126,982,061 |
| rs10986626 | 0.84 | 45 | 0.61 | 5.44x10^-01^ | 126,988,142 |
| rs599063 | 0.08 | 26 | 0.79 | 4.29x10^-01^ | 127,035,867 |
| rs391957 | 0.60 | 54 | 1.27 | 2.05x10^-01^ | 127,043,845 |
| rs432757 | 0.96 | 14 | 0.07 | 9.47x10^-01^ | 127,114,628 |
| rs13297440 | 0.45 | 66 | 0.93 | 3.51x10^-01^ | 127,127,021 |
| rs1463837 | 0.30 | 54 | 0.12 | 9.06x10^-01^ | 127,175,036 |
| rs4837016 | 0.42 | 60 | 0.56 | 5.74x10^-01^ | 127,181,630 |
| rs2648315 | 0.65 | 60 | 0.15 | 8.81x10^-01^ | 127,190,605 |
| rs3104554 | 0.60 | 68 | 0.23 | 8.16x10^-01^ | 127,192,436 |
| rs3122934 | 0.57 | 66 | 0.34 | 7.37x10^-01^ | 127,193,921 |
| rs2819624 | 0.64 | 66 | 0.80 | 4.24x10^-01^ | 127,196,340 |
| rs1250505 | 0.58 | 65 | 0.34 | 7.33x10^-01^ | 127,233,277 |
| rs6478704 | 0.37 | 59 | 0.70 | 4.84x10^-01^ | 127,238,243 |
| rs925473 | 0.62 | 63 | 0.34 | 7.33x10^-01^ | 127,267,847 |
| rs7018948 | 0.33 | 60 | 0.10 | 9.19x10^-01^ | 127,273,327 |
| rs10819050 | 0.40 | 62 | 0.00 | 1.00x10+00 | 127,276,999 |
| rs7046471 | 0.33 | 57 | 0.30 | 7.65x10^-01^ | 127,290,230 |
| rs13293034 | 0.65 | 66 | 0.40 | 6.87x10^-01^ | 127,296,772 |
| rs10986778 | 0.74 | 53 | 0.21 | 8.34x10^-01^ | 127,303,238 |
| rs534214 | 0.56 | 65 | 0.12 | 9.05x10^-01^ | 127,409,045 |
| rs484821 | 0.20 | 43 | 0.42 | 6.76x10^-01^ | 127,420,884 |
| rs605437 | 0.11 | 28 | 0.95 | 3.43x10^-01^ | 127,451,322 |
| rs871391 | 0.70 | 60 | 0.58 | 5.65x10^-01^ | 127,460,202 |
| rs551938 | 0.74 | 56 | 0.64 | 5.21x10^-01^ | 127,462,849 |
| rs7862834 | 0.14 | 40 | 0.93 | 3.51x10^-01^ | 127,488,612 |
| rs11793676 | 0.03 | 11 | 1.07 | 2.85x10^-01^ | 127,493,426 |
| rs10986848 | 0.63 | 62 | 0.12 | 9.07x10^-01^ | 127,501,710 |
| rs4837022 | 0.66 | 62 | 0.75 | 4.51x10^-01^ | 127,515,200 |
| rs11788358 | 0.26 | 50 | 0.83 | 4.05x10^-01^ | 127,519,910 |
| rs10986857 | 0.22 | 40 | 0.81 | 4.17x10^-01^ | 127,528,615 |
| rs12345049 | 0.26 | 47 | 1.02 | 3.07x10^-01^ | 127,539,435 |
| rs13295318 | 0.37 | 55 | 0.21 | 8.37x10^-01^ | 127,650,090 |
| rs10115938 | 0.66 | 62 | 0.78 | 4.34x10^-01^ | 127,668,392 |
| rs1952669 | 0.68 | 61 | 1.12 | 2.61x10^-01^ | 127,700,497 |
| rs1411351 | 0.40 | 57 | 0.64 | 5.20x10^-01^ | 127,704,231 |
| rs888230 | 0.70 | 58 | 1.31 | 1.89x10^-01^ | 127,764,125 |
| rs3829098 | 0.70 | 58 | 1.38 | 1.68x10^-01^ | 127,766,856 |
| rs1571570 | 0.09 | 24 | 1.09 | 2.74x10^-01^ | 127,788,070 |
| rs7854636 | 0.60 | 60 | 0.49 | 6.25x10^-01^ | 127,794,477 |
| rs4838305 | 0.50 | 64 | 0.51 | 6.07x10^-01^ | 127,802,003 |
| rs10819100 | 0.61 | 59 | 0.26 | 7.96x10^-01^ | 127,806,874 |
| rs1477149 | 0.51 | 62 | 1.00 | 3.19x10^-01^ | 127,811,466 |
| rs13291454 | 0.55 | 58 | 0.36 | 7.21x10^-01^ | 127,813,209 |
| rs867932 | 0.57 | 57 | 0.82 | 4.10x10^-01^ | 127,824,008 |
| rs2416993 | 0.17 | 37 | 1.54 | 1.25x10^-01^ | 127,837,372 |
| rs4838315 | 0.64 | 61 | 0.89 | 3.71x10^-01^ | 127,844,090 |
| rs2416995 | 0.87 | 31 | 0.12 | 9.08x10^-01^ | 127,847,170 |
| rs4838317 | 0.55 | 70 | 0.35 | 7.26x10^-01^ | 127,850,920 |
| rs888237 | 0.29 | 50 | 0.08 | 9.33x10^-01^ | 127,856,459 |
| rs888236 | 0.34 | 52 | 1.00 | 3.19x10^-01^ | 127,857,337 |
| rs888231 | 0.37 | 63 | 0.49 | 6.26x10^-01^ | 127,866,612 |
| rs10987122 | 0.46 | 58 | 0.77 | 4.44x10^-01^ | 127,868,537 |
| rs999566 | 0.37 | 58 | 0.06 | 9.56x10^-01^ | 127,875,623 |
| rs4838322 | 0.54 | 60 | 0.75 | 4.52x10^-01^ | 127,876,380 |
| rs2416996 | 0.60 | 61 | 0.10 | 9.21x10^-01^ | 127,883,987 |
| rs1420771 | 0.65 | 58 | 0.56 | 5.77x10^-01^ | 127,889,309 |
| rs868261 | 0.55 | 66 | 0.15 | 8.82x10^-01^ | 127,894,014 |
| rs2080558 | 0.12 | 31 | 1.50 | 1.35x10^-01^ | 127,900,525 |
| rs1861715 | 0.54 | 70 | 1.62 | 1.06x10^-01^ | 127,908,055 |
| rs888233 | 0.24 | 43 | 0.97 | 3.35x10^-01^ | 127,912,503 |
| rs6478724 | 0.27 | 55 | 1.05 | 2.94x10^-01^ | 127,916,337 |
| rs4595229 | 0.94 | 20 | 0.62 | 5.34x10^-01^ | 127,919,755 |
| rs4838341 | 0.67 | 60 | 1.07 | 2.85x10^-01^ | 127,920,121 |
| rs1861716 | 0.80 | 42 | 1.02 | 3.06x10^-01^ | 127,925,369 |
| rs2111366 | 0.48 | 52 | 0.75 | 4.51x10^-01^ | 127,943,701 |
| rs4838349 | 0.35 | 53 | 1.40 | 1.61x10^-01^ | 127,949,216 |
| rs1055353 | 0.08 | 29 | 1.34 | 1.81x10^-01^ | 127,950,044 |
| rs1055352 | 0.56 | 55 | 0.39 | 6.94x10^-01^ | 127,950,240 |
| rs888229 | 0.81 | 43 | 0.77 | 4.43x10^-01^ | 127,958,271 |
| rs4837043 | 0.61 | 62 | 0.56 | 5.73x10^-01^ | 127,962,503 |
| rs888228 | 0.38 | 56 | 1.72 | 8.63x10^-02^ | 127,965,578 |
| rs888225 | 0.92 | 24 | 0.53 | 5.97x10^-01^ | 127,965,912 |
| rs888221 | 0.17 | 42 | 0.11 | 9.09x10^-01^ | 127,968,352 |
| rs4838357 | 0.19 | 47 | 0.00 | 1.00x10+00 | 127,969,671 |
| rs1536961 | 0.65 | 68 | 0.48 | 6.30x10^-01^ | 127,977,343 |
| rs2809443 | 0.54 | 71 | 0.76 | 4.45x10^-01^ | 127,987,296 |
| rs1360288 | 0.66 | 61 | 0.68 | 4.99x10^-01^ | 128,007,884 |
| rs2417008 | 0.13 | 33 | 0.00 | 1.00x10+00 | 128,010,040 |
| rs2065183 | 0.35 | 67 | 0.46 | 6.45x10^-01^ | 128,018,289 |
| rs1571576 | 0.21 | 53 | 0.29 | 7.76x10^-01^ | 128,021,960 |
| rs1106229 | 0.39 | 60 | 0.18 | 8.56x10^-01^ | 128,027,655 |
| rs944221 | 0.84 | 44 | 0.61 | 5.39x10^-01^ | 128,028,320 |
| rs2773390 | 0.25 | 51 | 0.19 | 8.48x10^-01^ | 128,030,581 |
| rs2809428 | 0.63 | 62 | 0.40 | 6.91x10^-01^ | 128,040,850 |
| rs2809430 | 0.61 | 65 | 1.35 | 1.76x10^-01^ | 128,047,790 |
| rs10760423 | 0.27 | 57 | 1.92 | 5.53x10^-02^ | 128,059,069 |
| rs2773386 | 0.52 | 63 | 0.42 | 6.77x10^-01^ | 128,066,217 |
| rs2491650 | 0.61 | 62 | 0.55 | 5.83x10^-01^ | 128,068,960 |
| rs2809435 | 0.60 | 65 | 0.93 | 3.55x10^-01^ | 128,074,056 |
| rs951214 | 0.73 | 58 | 0.63 | 5.32x10^-01^ | 128,082,520 |
| rs2026808 | 0.38 | 68 | 1.02 | 3.08x10^-01^ | 128,089,513 |
| rs1317309 | 0.73 | 55 | 2.67 | 7.69x10^-03^ | 128,096,418 |
| rs944212 | 0.27 | 55 | 0.71 | 4.81x10^-01^ | 128,099,896 |
| rs539215 | 0.81 | 48 | 1.82 | 6.94x10^-02^ | 128,101,333 |
| rs1230760 | 0.60 | 59 | 0.12 | 9.05x10^-01^ | 128,102,657 |
| rs2015843 | 0.27 | 53 | 1.50 | 1.35x10^-01^ | 128,104,419 |
| rs550135 | 0.86 | 41 | 1.24 | 2.16x10^-01^ | 128,106,154 |
| rs1360258 | 0.49 | 62 | 1.75 | 8.08x10^-02^ | 128,111,569 |
| rs474359 | 0.79 | 49 | 0.39 | 6.93x10^-01^ | 128,113,072 |
| rs504184 | 0.72 | 63 | 0.11 | 9.16x10^-01^ | 128,114,805 |
| rs561769 | 0.26 | 59 | 0.33 | 7.44x10^-01^ | 128,115,735 |
| rs548645 | 0.45 | 68 | 0.92 | 3.57x10^-01^ | 128,120,224 |
| rs10819146 | 0.45 | 67 | 0.98 | 3.28x10^-01^ | 128,124,753 |
| rs10491514 | 0.73 | 61 | 0.79 | 4.31x10^-01^ | 128,141,839 |
| rs554480 | 0.78 | 51 | 0.33 | 7.40x10^-01^ | 128,147,964 |
| rs568565 | 0.22 | 55 | 1.05 | 2.94x10^-01^ | 128,155,202 |
| rs1360289 | 0.32 | 59 | 0.31 | 7.56x10^-01^ | 128,155,538 |
| rs505442 | 0.46 | 70 | 0.89 | 3.76x10^-01^ | 128,157,483 |
| rs10819148 | 0.83 | 49 | 0.24 | 8.14x10^-01^ | 128,166,315 |
| rs735290 | 0.23 | 62 | 1.18 | 2.37x10^-01^ | 128,168,089 |
| rs917777 | 0.45 | 67 | 0.82 | 4.11x10^-01^ | 128,183,136 |
| rs2286889 | 0.90 | 29 | 0.15 | 8.84x10^-01^ | 128,183,256 |
| rs917776 | 0.56 | 66 | 0.76 | 4.46x10^-01^ | 128,186,165 |
| rs10819155 | 0.68 | 52 | 0.56 | 5.77x10^-01^ | 128,190,707 |
| rs10987268 | 0.86 | 44 | 0.42 | 6.73x10^-01^ | 128,209,802 |
| rs13298216 | 0.11 | 35 | 0.31 | 7.60x10^-01^ | 128,210,726 |
| rs758971 | 0.81 | 47 | 0.38 | 7.05x10^-01^ | 128,215,611 |
| rs10760432 | 0.52 | 62 | 0.97 | 3.32x10^-01^ | 128,216,551 |
| rs10760433 | 0.32 | 64 | 1.03 | 3.01x10^-01^ | 128,218,334 |
| rs2286888 | 0.21 | 55 | 0.86 | 3.88x10^-01^ | 128,223,912 |
| rs758970 | 0.61 | 50 | 1.36 | 1.75x10^-01^ | 128,234,594 |
| rs2041543 | 0.15 | 42 | 0.68 | 4.96x10^-01^ | 128,237,400 |
| rs2417017 | 0.12 | 25 | 0.09 | 9.26x10^-01^ | 128,243,257 |
| rs7043602 | 0.26 | 57 | 0.13 | 8.97x10^-01^ | 128,245,321 |
| rs10122788 | 0.41 | 63 | 0.94 | 3.50x10^-01^ | 128,246,653 |
| rs12345602 | 0.47 | 63 | 1.20 | 2.31x10^-01^ | 128,256,287 |
| rs12683683 | 0.35 | 54 | 1.23 | 2.21x10^-01^ | 128,264,508 |
| rs1468675 | 0.74 | 48 | 1.24 | 2.14x10^-01^ | 128,271,778 |
| rs10114851 | 0.15 | 42 | 0.64 | 5.25x10^-01^ | 128,274,192 |
| rs7047946 | 0.37 | 63 | 0.03 | 9.80x10^-01^ | 128,276,445 |
| rs4837090 | 0.27 | 49 | 0.64 | 5.19x10^-01^ | 128,277,752 |
| rs2286886 | 0.53 | 60 | 0.57 | 5.66x10^-01^ | 128,285,998 |
| rs887651 | 0.25 | 54 | 0.61 | 5.43x10^-01^ | 128,292,498 |
| rs7031327 | 0.06 | 19 | 0.71 | 4.75x10^-01^ | 128,294,129 |
| rs10819172 | 0.10 | 25 | 0.81 | 4.17x10^-01^ | 128,295,667 |
| rs3814127 | 0.73 | 53 | 0.99 | 3.23x10^-01^ | 128,305,563 |
| rs7024765 | 0.62 | 59 | 0.87 | 3.82x10^-01^ | 128,313,912 |
| rs4837096 | 0.45 | 66 | 0.43 | 6.69x10^-01^ | 128,315,873 |
| rs2417024 | 0.59 | 64 | 0.87 | 3.83x10^-01^ | 128,320,931 |
| rs868764 | 0.05 | 17 | 0.73 | 4.66x10^-01^ | 128,321,469 |
| rs885618 | 0.19 | 42 | 1.25 | 2.11x10^-01^ | 128,322,178 |
| rs12001841 | 0.78 | 45 | 0.17 | 8.64x10^-01^ | 128,324,316 |
| rs10987324 | 0.93 | 25 | 0.46 | 6.47x10^-01^ | 128,325,906 |
| rs10739679 | 0.63 | 54 | 0.31 | 7.60x10^-01^ | 128,334,292 |
| rs2152749 | 0.22 | 44 | 0.52 | 6.02x10^-01^ | 128,339,357 |
| rs4448386 | 0.73 | 60 | 0.98 | 3.26x10^-01^ | 128,345,835 |
| rs7874085 | 0.71 | 55 | 1.84 | 6.65x10^-02^ | 128,347,004 |
| rs3916192 | 0.46 | 58 | 0.99 | 3.22x10^-01^ | 128,353,778 |
| rs10819182 | 0.25 | 47 | 2.39 | 1.70x10^-02^ | 128,360,387 |
| rs3861870 | 0.87 | 41 | 1.79 | 7.38x10^-02^ | 128,363,842 |
| rs3904195 | 0.65 | 57 | 1.49 | 1.35x10^-01^ | 128,374,521 |
| rs4144234 | 0.39 | 65 | 2.19 | 2.86x10^-02^ | 128,379,656 |
| rs4086192 | 0.22 | 45 | 0.48 | 6.33x10^-01^ | 128,396,663 |
| rs10733680 | 0.13 | 39 | 1.68 | 9.29x10^-02^ | 128,401,776 |
| rs2235055 | 0.06 | 25 | 0.28 | 7.82x10^-01^ | 128,416,897 |
| rs4455975 | 0.57 | 73 | 0.33 | 7.41x10^-01^ | 128,423,020 |
| rs3829849 | 0.63 | 69 | 0.63 | 5.31x10^-01^ | 128,430,621 |
| rs10760443 | 0.07 | 19 | 0.87 | 3.84x10^-01^ | 128,435,167 |
| rs6478750 | 0.58 | 64 | 0.50 | 6.15x10^-01^ | 128,449,019 |
| rs944103 | 0.47 | 46 | 0.49 | 6.26x10^-01^ | 128,453,311 |
| rs3902840 | 0.07 | 26 | 0.00 | 1.00x10+00 | 128,458,846 |
| rs7857133 | 0.51 | 56 | 0.73 | 4.69x10^-01^ | 128,460,457 |
| rs12343574 | 0.88 | 34 | 0.38 | 7.01x10^-01^ | 128,480,661 |
| rs10987411 | 0.56 | 64 | 0.82 | 4.15x10^-01^ | 128,490,260 |
| rs10987414 | 0.63 | 61 | 0.86 | 3.89x10^-01^ | 128,499,299 |
| rs10733682 | 0.51 | 70 | 0.47 | 6.35x10^-01^ | 128,500,735 |
| rs3906146 | 0.44 | 71 | 0.58 | 5.62x10^-01^ | 128,503,434 |
| rs867559 | 0.84 | 49 | 0.23 | 8.19x10^-01^ | 128,505,146 |
| rs869499 | 0.07 | 19 | 0.03 | 9.74x10^-01^ | 128,506,006 |
| rs7870976 | 0.35 | 59 | 0.37 | 7.15x10^-01^ | 128,511,718 |

OT: overtransmitted to affected children.

^a^ Frequency of the OT allele. In the case of balanced transmission (Z = 0) or insufficient allelic frequency to perform the association test (*** in Z column), the minor allele frequency is indicated.

^b^ Absolute value of the FBAT statistic.

^c^ Association *P*-value.
