# Supplementary material for: Comprehensive Linkage and Association Analyses Identify Haplotype, Near to the TNFSF15 Gene, Significantly Associated with Spondyloarthritis
Source: PLoS Genet. 2009 Jun 19;5(6):e1000528. doi: 10.1371/journal.pgen.1000528 (PMC2689651; doi:10.1371/journal.pgen.1000528)
Supplement: Table S2 — Complete results of the family-based association extension study (TaqMan genotyping - 287 families). (0.08 MB DOC) [file pgen.1000528.s002.doc]

**Table S2.** Complete results of the family-based association extension study (TaqMan genotyping - 287 families).

| Marker | OT  allele name | OT  allele frequency | UT  allele name | Number of informative families | Z^a^ | nominal  *P-*value^b^ | Position  (bp) |
| --- | --- | --- | --- | --- | --- | --- | --- |
| rs11788229 | C | 0.90 | T | 72 | 0.44 | 6.60x10^-01^ | 116,491,717 |
| rs4979455 | G | 0.42 | A | 124 | 1.71 | 8.69x10^-02^ | 116,496,031 |
| rs10982373 | A | 0.42 | G | 137 | 0.83 | 4.08x10^-01^ | 116,503,083 |
| rs11793039 | C | 0.11 | A | 68 | 0.44 | 6.61x10^-01^ | 116,504,063 |
| rs17816047 | C | 0.12 | A | 73 | 0.03 | 9.77x10^-01^ | 116,504,195 |
| rs4979459 | T | 0.54 | G | 140 | 2.81 | 4.96x10^-03^ | 116,521,487 |
| **rs7849556** | **A** | **0.77** | **C** | **112** | **3.06** | **2.24x10^-03^** | **116,522,493** |
| **rs10817669** | **A** | **0.75** | **G** | **131** | **3.68** | **2.37x10^-04^** | **116,522,836** |
| **rs10739427** | **G** | **0.77** | **T** | **130** | **3.56** | **3.72x10^-04^** | **116,536,156** |
| **rs10759734** | **A** | **0.78** | **G** | **128** | **3.16** | **1.60x10^-03^** | **116,536,471** |
| rs6478105 | A | 0.87 | G | 88 | 1.85 | 6.40x10^-02^ | 116,557,006 |
| rs10982396 | C | 0.87 | G | 86 | 1.40 | 1.62x10^-01^ | 116,558,750 |
| rs10982399 | C | 0.51 | T | 145 | 2.28 | 2.24x10^-02^ | 116,560,850 |
| **rs10733612** | **C** | **0.78** | **T** | **123** | **3.14** | **1.67x10^-03^** | **116,562,871** |
| rs10982402 | G | 0.73 | A | 116 | 0.63 | 5.30x10^-01^ | 116,565,300 |
| rs12335468 | T | 0.91 | C | 73 | 2.73 | 6.35x10^-03^ | 116,571,195 |
| rs12238270 | T | 0.77 | G | 117 | 0.97 | 3.32x10^-01^ | 116,574,875 |
| rs12237465 | G | 0.78 | C | 109 | 1.63 | 1.04x10^-01^ | 116,575,086 |
| rs4246905 | C | 0.71 | T | 129 | 1.90 | 5.78x10^-02^ | 116,593,070 |
| rs6478108 | T | 0.67 | C | 130 | 2.11 | 3.46x10^-02^ | 116,598,524 |
| rs7030574 | A | 0.50 | C | 148 | 1.62 | 1.06x10^-01^ | 116,607,870 |
| rs6478109 | G | 0.68 | A | 130 | 1.71 | 8.65x10^-02^ | 116,608,587 |
| rs7848647 | C | 0.67 | T | 126 | 1.87 | 6.16x10^-02^ | 116,608,867 |
| rs10982412 | A | 0.13 | G | 63 | 0.83 | 4.06x10^-01^ | 116,610,677 |
| rs10982414 | C | 0.85 | G | 72 | 0.10 | 9.21x10^-01^ | 116,614,045 |
| rs10817677 | T | 0.16 | C | 85 | 0.03 | 9.80x10^-01^ | 116,615,746 |
| rs12237626 | G | 0.86 | A | 79 | 0.01 | 9.96x10^-01^ | 116,616,121 |
| rs7865494 | C | 0.84 | T | 84 | 0.17 | 8.67x10^-01^ | 116,616,300 |
| rs7866379 | T | 0.17 | C | 88 | 0.13 | 8.99x10^-01^ | 116,617,058 |
| rs12340243 | G | 0.16 | T | 85 | 0.37 | 7.14x10^-01^ | 116,620,999 |
| rs17292046 | T | 0.87 | G | 66 | 0.27 | 7.84x10^-01^ | 116,626,913 |

OT: allele overtransmitted to affected children; UT: allele undertransmitted to affected children. Markers that yielded a significant association *P*-value appear in bold.

^a^ Absolute value of the FBAT statistic.

^b^ Association *P*-value. The nominal *P*-values to achieve global type I errors of 5 % and 7.5 % significance, using Bonferroni correction for 31 tests were 1.61 x 10^-3^ and 2.42 x 10^-3^ respectively. Markers that have achieved at least one of these thresholds are in bold.
