# Supplementary material for: Comprehensive Linkage and Association Analyses Identify Haplotype, Near to the TNFSF15 Gene, Significantly Associated with Spondyloarthritis
Source: PLoS Genet. 2009 Jun 19;5(6):e1000528. doi: 10.1371/journal.pgen.1000528 (PMC2689651; doi:10.1371/journal.pgen.1000528)
Supplement: Table S3 — Complete results of case/control association extension study (TaqMan genotyping - 139 SpA cases/163 healthy controls). (0.07 MB DOC) [file pgen.1000528.s003.doc]

**Table S3.** Complete results of case/control association extension study (TaqMan genotyping - 139 SpA cases/163 healthy controls).

| SNP | Position | Minor allele | Minor allele frequencies | | Allele counts | | Major allele | Nominal  *P*-value^a^ | OR | HW^b^ |
| --- | --- | --- | --- | --- | --- | --- | --- | --- | --- | --- |
|  |  |  | Cases | Controls | Cases | Controls |  |  |  |  |
| rs11788229 | 116,491,717 | T | 0.139 | 0.100 | 38/236 | 32/288 | C | 0.145 | 1.45 | 0.3 |
| rs4979455 | 116,496,031 | G | 0.456 | 0.452 | 123/147 | 141/171 | A | 0.930 | 1.02 | 1.0 |
| rs10982373 | 116,503,083 | A | 0.416 | 0.433 | 114/160 | 136/178 | G | 0.676 | 0.93 | 0.6 |
| rs11793039 | 116,504,063 | C | 0.088 | 0.099 | 24/248 | 31/281 | A | 0.646 | 0.88 | 0.1 |
| rs17816047 | 116,504,195 | C | 0.094 | 0.101 | 25/241 | 32/286 | A | 0.788 | 0.93 | 0.1 |
| rs4979459 | 116,521,487 | G | 0.454 | 0.500 | 119/143 | 156/156 | T | 0.274 | 0.83 | 0.1 |
| rs7849556 | 116,522,493 | C | 0.201 | 0.266 | 55/219 | 58/160 | A | 0.087 | 0.69 | 0.6 |
| rs10817669 | 116,522,836 | G | 0.271 | 0.326 | 72/194 | 101/209 | A | 0.150 | 0.77 | 0.5 |
| rs10739427 | 116,536,156 | T | 0.195 | 0.258 | 53/219 | 81/233 | G | 0.070 | 0.70 | 0.5 |
| rs10759734 | 116,536,471 | G | 0.195 | 0.272 | 53/219 | 87/233 | A | 0.028 | 0.65 | 0.6 |
| **rs6478105** | **116,557,006** | **G** | **0.063** | **0.171** | **17/253** | **54/262** | **A** | **6.6x10^-05^** | **0.33** | 0.6 |
| **rs10982396** | **116,558,750** | **G** | **0.064** | **0.173** | **17/249** | **53/253** | **C** | **6.9x10^-05^** | **0.33** | 0.6 |
| rs10982399 | 116,560,850 | T | 0.474 | 0.516 | 129/143 | 162/152 | C | 0.315 | 0.85 | 0.2 |
| rs10733612 | 116,562,871 | T | 0.195 | 0.270 | 51/211 | 83/225 | C | 0.036 | 0.66 | 0.9 |
| rs10982402 | 116,565,300 | A | 0.270 | 0.258 | 73/197 | 81/233 | G | 0.734 | 1.07 | 1.0 |
| rs12335468 | 116,571,195 | C | 0.107 | 0.091 | 29/243 | 29/289 | T | 0.531 | 1.19 | 0.5 |
| rs12238270 | 116,574,875 | G | 0.197 | 0.204 | 54/220 | 65/253 | T | 0.825 | 0.96 | 0.6 |
| rs12237465 | 116,575,086 | C | 0.178 | 0.185 | 46/212 | 58/256 | G | 0.843 | 0.96 | 1.0 |
| rs4246905 | 116,593,070 | T | 0.254 | 0.341 | 68/200 | 107/207 | C | 0.022 | 0.66 | 0.8 |
| rs6478108 | 116,598,524 | C | 0.288 | 0.369 | 79/195 | 116/198 | T | 0.037 | 0.69 | 0.9 |
| rs7030574 | 116,607,870 | A | 0.482 | 0.487 | 131/141 | 155/163 | C | 0.888 | 0.98 | 0.8 |
| rs6478109 | 116,608,587 | A | 0.288 | 0.354 | 79/195 | 114/208 | G | 0.088 | 0.74 | 0.7 |
| rs7848647 | 116,608,867 | T | 0.284 | 0.359 | 76/192 | 112/200 | C | 0.053 | 0.71 | 0.7 |
| rs10982412 | 116,610,677 | A | 0.119 | 0.111 | 32/238 | 35/281 | G | 0.769 | 1.08 | 0.6 |
| rs10982414 | 116,614,045 | G | 0.147 | 0.134 | 39/227 | 42/272 | C | 0.656 | 1.11 | 1.0 |
| rs10817677 | 116,615,746 | T | 0.169 | 0.129 | 46/226 | 41/277 | C | 0.170 | 1.38 | 1.0 |
| rs12237626 | 116,616,121 | A | 0.139 | 0.122 | 38/236 | 39/281 | G | 0.543 | 1.16 | 1.0 |
| rs7865494 | 116,616,300 | T | 0.180 | 0.131 | 49/223 | 42/278 | C | 0.100 | 1.45 | 1.0 |
| rs7866379 | 116,617,058 | T | 0.200 | 0.147 | 54/216 | 47/273 | C | 0.088 | 1.45 | 0.7 |
| rs12340243 | 116,620,999 | G | 0.185 | 0.132 | 50/220 | 42/276 | T | 0.077 | 1.49 | 1.0 |
| rs17292046 | 116,626,913 | G | 0.096 | 0.114 | 25/235 | 36/280 | T | 0.490 | 0.83 | 0.5 |

SNP: single-nucleotide polymorphism; OR: odds-ratio; HW: Hardy-Weinberg.

^a^ Asymptotic *P*-values for chi square comparisons of allele counts between cases and controls. The nominal P-value to achieve global type I error of 5 % significance, using Bonferroni correction for 31 tests was 1.61 x 10^-3^. Markers that have achieved this threshold are represented in bold.

^b^ Hardy-Weinberg test for all individuals exact *P*-value.
