# Supplementary material for: Comprehensive Linkage and Association Analyses Identify Haplotype, Near to the TNFSF15 Gene, Significantly Associated with Spondyloarthritis
Source: PLoS Genet. 2009 Jun 19;5(6):e1000528. doi: 10.1371/journal.pgen.1000528 (PMC2689651; doi:10.1371/journal.pgen.1000528)
Supplement: Table S5 — Single SNP (1), and haplotype-based (2) association results in HLA-B27 negative case/control sample (73 SpA cases/255 healthy controls). (0.05 MB DOC) [file pgen.1000528.s005.doc]

**Table S5.** Single SNP (1), and haplotype-based (2) association results in HLA-B27 negative case/control sample (73 SpA cases/255 healthy controls).

1- Single SNP association results

| SNP | Minor allele | Minor allele freq. | | *P* | OR |
| --- | --- | --- | --- | --- | --- |
|  |  | Cases | Controls |  |  |
| rs4979459 | G | 0.38 | 0.48 | 0.030 | 0.7 (0.4-1.0) |
| rs7849556 | C | 0.18 | 0.24 | 0.171 | 0.7 (0.4-1.2) |
| rs10817669 | G | 0.20 | 0.30 | 0.030 | 0.6 (0.4-1.0) |
| rs10759734 | G | 0.18 | 0.26 | 0.052 | 0.6 (0.4-1.0) |
| rs6478105 | G | 0.05 | 0.17 | 0.000 | 0.3 (0.1-0.6) |
| rs10982396 | G | 0.06 | 0.17 | 0.001 | 0.3 (0.1-0.6) |
| rs10733612 | T | 0.19 | 0.25 | 0.149 | 0.7 (0.4-1.1) |
| rs4246905 | T | 0.21 | 0.34 | 0.005 | 0.5 (0.3-0.8) |

2 - Haplotype association results

| SNP | Frequency | | DF | *P* |
| --- | --- | --- | --- | --- |
|  | Cases | Controls |  |  |
| OMNIBUS | NA | NA | 3 | 0.016 |
| AAAACC | 0.81 | 0.71 | 1 | 0.028 |
| CGGGGT | 0.05 | 0.16 | 1 | 0.002 |
| CGGACT | 0.10 | 0.09 | 1 | 0.542 |
| AGAACC | 0.03 | 0.04 | 1 | 0.663 |

SNP: single-nucleotide polymorphism; OR: odds-ratio; *P*: asymptotic association *P*-value; DF: degree of freedom.
