# Supplementary material for: Comprehensive Linkage and Association Analyses Identify Haplotype, Near to the TNFSF15 Gene, Significantly Associated with Spondyloarthritis
Source: PLoS Genet. 2009 Jun 19;5(6):e1000528. doi: 10.1371/journal.pgen.1000528 (PMC2689651; doi:10.1371/journal.pgen.1000528)
Supplement: Table S6 — Results of combined family-based and case/control association-extension studies for eight SPA2 single-nucleotide polymorphisms (SNPs). (0.04 MB DOC) [file pgen.1000528.s006.doc]

**Table S6. Results of combined family-based and case/control association-extension^a^ studies for eight SPA2 single-nucleotide polymorphisms (SNPs).**

| SNP name | Minor allele | Observed | Expected | Chi2 | P-value^b^ |
| --- | --- | --- | --- | --- | --- |
| rs4979459 | G | 485 | 522 | 9.9 | 0.0016 |
| rs7849556 | C | 218 | 250 | 11.1 | 8.53x10^-4^ |
| rs10817669 | G | 256 | 298 | 16.3 | 5.52x10^-5^ |
| rs10759734 | G | 225 | 264 | 14.6 | 1.33x10^-4^ |
| rs6478105 | G | 115 | 144 | 13.3 | 2.65x10^-4^ |
| rs10982396 | G | 117 | 143 | 11.1 | 8.88x10^-4^ |
| rs10733612 | T | 213 | 250 | 13.9 | 1.97x10^-4^ |
| rs4246905 | T | 311 | 341 | 7.8 | 0.0051 |

^a^ Refer to Figure 1 for the study design.

^b^ Asymptotic *P*-values computed with the Cochran-Mantel-Haenszel test.
